# A guided single session intervention to reduce intrusive memories of work-related trauma: a randomised controlled trial with healthcare workers in the COVID-19 pandemic

**DOI:** 10.1186/s12916-024-03569-8

**Published:** 2024-09-19

**Authors:** Marie Kanstrup, Laura Singh, Elisabeth Johanna Leehr, Katarina E. Göransson, Sara Ahmed Pihlgren, Lalitha Iyadurai, Oili Dahl, Ann-Charlotte Falk, Veronica Lindström, Nermin Hadziosmanovic, Katja Gabrysch, Michelle L. Moulds, Emily A. Holmes

**Affiliations:** 1 Division of Psychology, Department of Clinical Neuroscience, Karolinska Institutet, Stockholm, Sweden; 2 Department of Psychology, Uppsala University, Uppsala, Sweden; 3 Behavioral Medicine, Theme Women’s Health and Allied Health Professionals, Karolinska University Hospital, Stockholm, Sweden; 4 Swedish Collegium for Advanced Study, Uppsala, Sweden; 5 Institute for Translational Psychiatry, University of Münster, Münster, Germany; 6 Emergency and Reparative Medicine Theme, Karolinska University Hospital, Stockholm, Sweden; 7 Department of Medicine, Solna, Karolinska Institutet, Stockholm, Sweden; 8 School of Health and Welfare, Department of Caring Sciences, Dalarna University, Falun, Sweden; 9 P1vital Products Ltd, Wallingford, Oxfordshire UK; 10 Division of Nursing, Department of Neurobiology, Care Sciences and Society, Karolinska Institutet, Stockholm, Sweden; 11 Department of Perioperativ Medicin and Intensive Care, Karolinska University Hospital, Stockholm, Sweden; 12 Department for Health Promoting Science, Sophiahemmet University, Stockholm, Sweden; 13 Department of Nursing, Section of Ambulance Service Region of Västerbotten, Umeå University, Umeå, Sweden; 14 Uppsala Clinical Research Center, Uppsala University, Uppsala, Sweden; 15 School of Psychology, The University of New South Wales, UNSW Sydney, Sydney, Australia; 16 Department of Women’s and Children’s Health, Uppsala University, Akademiska Sjukhuset, 751 85 Uppsala, Sweden

**Keywords:** Intrusive memory, Psychological trauma, Digital intervention, Healthcare workers, Post-traumatic stress disorder, Mental health, Pandemic, Prevention-to-treating

## Abstract

**Background:**

Intrusive memories of psychologically traumatic events bring distress both sub-clinically and clinically. This parallel-group, two-arm randomised controlled trial evaluated the effect of a brief behavioural intervention on reducing intrusive memories in frontline healthcare workers exposed to traumatic events during the COVID-19 pandemic.

**Methods:**

Participants with at least two intrusive memories of work-related trauma in the week before recruitment were randomised 1:1 to an imagery-competing task intervention (*n* = 73) or attention-based control task (*n* = 71). The number of intrusive memories was assessed at baseline and 5 weeks after the guided session (primary endpoint).

**Results:**

The intervention significantly reduced intrusive memory frequency compared with control [intervention Mdn = 1.0 (IQR = 0–3), control Mdn = 5.0 (IQR = 1–17); *p* < 0.0001, IRR = 0.30; 95% CI = 0.17–0.53] and led to fewer post-traumatic stress-related symptoms at 1, 3 and 6 month follow-ups (secondary endpoints). Participants and statisticians were blinded to allocation. Adverse events data were acquired throughout the trial, demonstrating safety. There was high adherence and low attrition.

**Conclusions:**

This brief, single-symptom, repeatable digital intervention for subclinical-to-clinical samples after trauma allows scalability, taking a preventing-to-treating approach after trauma.

**Trial registration:**

2020–07-06, ClinicalTrials.gov identifier: NCT04460014.

**Supplementary Information:**

The online version contains supplementary material available at 10.1186/s12916-024-03569-8.

## Background

Trauma exposure can result in intrusive memories of the traumatic events with negative sequelae for mental health. Exposure to work-related trauma – e.g. witnessing severe injury, dying and death—is common for frontline healthcare workers. When the World Health Organization declared COVID-19 a pandemic, early warnings for global mental health highlighted risks for healthcare workers [1] due to their increased work with severely ill patients. High rates of post-traumatic stress reactions, depression and anxiety were documented in healthcare staff prior to the pandemic [2–4], and were further elevated as the pandemic took hold [5–7]. Accordingly, the development of effective, brief, scalable interventions for healthcare workers (and other trauma-exposed populations) that can help with reduction of symptoms, and are in a format that would facilitate global reach, is imperative [8, 9].

The first-line treatments recommended in international guidelines for posttraumatic stress disorder (including chronic PTSD) ahead of medications are psychological interventions: trauma-focused cognitive behavioural therapy (TFCBT) and eye movement desensitisation and reprocessing (EMDR) [10–13]. A recent network meta-analysis of trials on the treatment of PTSD concluded that both interventions with and without trauma-focus are effective and acceptable [14]. TFCBT yielded the highest efficacy, with slightly more patients discontinuing TFCBT than non-trauma-focused interventions. Even the best psychological treatments leave room for further improvement [15, 16]. Further challenges of current recommended treatments after trauma include stigma and barriers to care alongside high rates of patient dropout [17], as well as the need for scheduling relatively lengthy sessions with a psychotherapist. Overall, this has led to a call for a more substantive evolution in treatments after psychological trauma [17].

We argue that the marked heterogeneity in PTSD with 636,120 possible symptom combinations [18] hampers scalable treatment development. An alternative approach is to focus on one core symptom [19, 20]. Intrusive memories are a candidate as they occur in the majority of clinical presentations post-trauma [21], are linked to other symptoms [22–24] and may be a precursor for later disorder. They also occur pre-clinically and can be distressing and unwanted in their own right [25]. Taken together, such evidence has prompted the idea of developing a *single-symptom focused intervention* targeting intrusive memories [25, 26] both in prevention and treatment.

Intrusive memories are the core clinical feature of posttraumatic stress disorder (PTSD) [27]. They comprise emotionally-laden mental images that intrude *involuntarily* into consciousness, rather than being deliberately recalled [28]. As well as being distressing, intrusive memories can have a debilitating impact on functioning [25] and disrupt concentration [29]. For a diagnosis of PTSD, the gold standard assessment tool, the Clinician-Administered PTSD Scale for DSM–5 (CAPS-5) [30], requires at least two intrusive memories over the past month. The CAPS-5 maximum score is ‘daily’ (i.e. 7 per week) with the mild-minimum score as ‘once-or-twice a week’/ ‘never’. In clinical reality, the number varies between individuals and samples and can be much higher than 7 per week. For example, the frequency of intrusive memories reported in patients with PTSD varies, e.g., 1.5 per week in a sample of PTSD outpatients [31] to 74 per week in PTSD related to childhood sexual abuse [32].

One potential way of mitigating the abovementioned challenges to accessing treatment for PTSD is to develop complementary approaches to existing interventions. To do so, we seek to tackle psychological treatment after trauma from various new angles; specifically, via i) a single symptom approach (i.e., targeting a single symptom rather than the full diagnosis of PTSD), ii) a subclinical-to-clinical sample (i.e., developing an intervention to target individuals with both sub-clinical and clinical symptoms); iii) preventing-to-treating approach (i.e., developing an intervention that could be used from the day of trauma (prevention) to months later (treatment) with scope for prevention and treatment; iv) a brief repeatable approach (given many face ongoing trauma exposure); v) mechanistically-informed intervention development inspired by neuroscientific models of memory updating; vi) targeting perceptual processing (imagery rather than words) avoiding the need to discuss trauma in detail; vii) a digital approach which requires only one guided session, with potential for scalability. We note that this proposed approach is not a treatment for PTSD—a disorder that can only be diagnosed 1-month post-trauma. Rather, it is an intervention for intrusive memories after trauma (here based on a minimum of 2 intrusive memories per week), which could be used from soon after trauma to months later, taking a preventing-to-treating approach for subclinical-to-clinical samples.

A recently developed imagery-competing task intervention (ICTI) is a brief behavioural approach involving a cognitive task, aimed at preventing and reducing the occurrence of intrusive memories [33, 34] by targeting their perceptual nature [35]. Informed by cognitive neuroscience [36], the intervention involves a memory reminder cue to the specific visual part of a trauma memory associated with an intrusion (hotspot [28]), followed by an imagery-based visuospatial task such as playing the computer game Tetris® for approximately 20 min, utilizing mental rotation (i.e., imagining rotating upcoming blocks for placement as part of gameplay [37–39]). Tetris is just one example of a visuospatial task that is theorised to compete with the same cognitive resources as the mental imagery [40] underlying intrusive memories. The brief ICTI intervention was derived as an alternative yet complementary approach to trauma-focused psychological interventions requiring the patient to repeatedly talk about the trauma in greater detail or the application of pharmacological approaches [13].

In the laboratory (i.e., using trauma analogues [39]) and following real trauma exposure [41–44], individuals who received an imagery-competing task intervention within hours after trauma have reported fewer intrusive memories relative to control tasks. In small-scale early stage clinical studies, the intervention showed promise when delivered at longer time intervals after trauma; e.g., to in-patients with chronic PTSD [45], refugees [46], and women with a chronic trauma history [47].

In a proof-of-concept randomised controlled trial (RCT) delivered in the emergency department on the same day as the trauma occurred, one session of the imagery-competing task intervention prevented development of intrusive memories in survivors of road traffic accidents by 62% [43]. In a similar emergency department-study with patients exposed to a range of traumas, those who received the intervention reported 48% fewer intrusive memories at one-week post-intervention than controls, and 90% fewer intrusions at week 5 post-intervention [44]. A subsequent RCT was commenced but terminated due to restrictions during the COVID-19 pandemic [48]. Throughout this period, our healthcare collaborators reported significant psychological distress associated with frontline work including intrusive memories of work-related trauma. An illustrative example of an intrusive memory after witnessing a traumatic event such as a child’s death in intensive care is “*I see the young patient’s eyes while being connected to the ventilator*”—a mental image intruding several times a week, bringing great distress and potentially impairing the staff member’s ability to use the ventilator in the future.

Informed by lived experience input from registered nurse collaborators about how to best adapt this new approach to their needs, we developed a tailored digital version of the intervention for the current study, which addressed the practical constraints and accessibility needs of frontline healthcare workers at the peak of the pandemic [49]. Critically, rather than only being delivered on the first day on which a traumatic event occurred, this version of the intervention could be delivered days, weeks or months later. In a Bayesian optimisation trial with National Health Service staff who worked in intensive care units in the United Kingdom during the pandemic (ClinicalTrials.gov identifier: NCT04992390) [37, 38]**,** participants either received immediate or 4-week-delayed access to the tailored intervention (i.e., a waitlist comparator arm). The study was designed to develop and optimise the intervention within a digital platform. The Bayesian analysis found strong evidence for a positive effect of the intervention as participants in the immediate arm (*n* = 36) reported 78% fewer intrusive memories compared to the delayed arm (*n* = 39). Key limitations of this early phase study were the use of a waitlist control with usual care (as opposed to an active control as the comparator), the recruitment of ICU staff only and the failure to examine if effects persisted beyond 4 weeks.

The current trial investigated the effectiveness of the brief imagery-competing task intervention in reducing intrusive memories in healthcare workers in Sweden exposed to work-related trauma during the pandemic, relative to an active control (an attention-based placebo control, involving listening to a podcast on philosophy [44]). To maximise practicality, reduce the possibility of virus transmission and facilitate access for healthcare workers continuing to work throughout the pandemic, both the intervention and control arm were delivered in a digital, remotely-delivered format. The intervention was tailored with collaborators with expertise through lived experience of working in the pandemic [49].

Participants in both arms were told they would receive a cognitive task. In the intervention arm, the cognitive task included a game. In the control arm, the task was a podcast – another type of widely-used smartphone application (for cultural relevance, we selected a podcast from national public radio). The podcast was intended to control for delivery device (smartphone), researcher contact time (one guided session), expectation effects (measured through credibility ratings), attention demands and remote delivery requirements to navigate blended digital materials (electronic platform and links to external websites) [50]. This task was developed in our earlier work in a hospital setting [44]. Given that the theory on which the intervention was developed posits that the mental imagery-competing nature of the task is important [51], we opted to employ a control task that used an auditory modality (rather than utilising another visuospatial task).

We hypothesised that participants who received the intervention would report fewer intrusive memories at week 5 post-intervention (primary outcome) relative to the active control arm. For secondary outcomes, we hypothesised that participants in the intervention arm would also report fewer intrusive memories (week 1 post-intervention), less severe related clinical symptoms of post-traumatic stress and associated distress, and better functioning. Information about adverse events (AEs) was acquired throughout the study via scheduled questions and free reporting.

## Methods

### Trial design

This was a randomised controlled trial with two parallel arms designed to compare the efficacy of a remotely-delivered cognitive task intervention (a memory cue followed by playing the computer game "Tetris" with mental rotation instructions) to an active attention placebo control (a cognitive task also involving a digital activity and of the same duration) in reducing intrusive memories of trauma in healthcare workers working during the COVID-19 pandemic. Both brief behavioural intervention arms involved one initial researcher-guided session, and participants could use their own smartphone for digital delivery. The trial was registered prior to study start (July 7, 2020; clinicaltrials.gov identifier: NCT04460014) and a protocol paper published [52]. The study was monitored by an independent clinical trials unit (Karolinska Trial Alliance) and statistical analyses as defined in the Statistical Analysis Plan (see Open Science Framework (OSF): https://osf.io/mb54w/) were performed by independent statistical services at Uppsala Clinical Research Center.

### Participants

Participants (*n* = 164) were healthcare workers working during the COVID-19 pandemic recruited via posters and information materials posted in health care facilities in Sweden, digital hospital resources, social media, a study webpage, research recruitment websites, recommendations from clinical colleagues and management, and newspapers.

Inclusion criteria were: aged 18 or over; engaged in clinical work during the COVID-19 pandemic in hospital and/or care facility; experienced at least one traumatic event according to The Diagnostic and Statistical Manual of Mental Disorders-PTSD Criterion A (e.g., exposure to actual or threatened death) [27] as a healthcare worker during the COVID-19 pandemic; had intrusive memories of the traumatic event(s); and had experienced at least two intrusive memories in the week prior to enrolment. Participants also needed to be able and willing to write down these memories in brief; have access to an internet- enabled smartphone and sufficient physical mobility to use their smartphone; be alert and oriented; and be fluent in spoken and written Swedish. Exclusion criteria were: loss of consciousness for > 5 min in relation to the traumatic event; and intoxication during the traumatic event or at time of study enrolment.

### Randomisation and blinding

Randomisation took place following completion of baseline assessments and prior to commencement of the intervention/control session. This was automated to promote allocation concealment. Participants were randomised in a 1:1 ratio to the assigned group using a computerised randomisation tool implemented in the electronic data collection platform SMART-TRIAL (web-based computer-generated allocation). Block randomisation with random block sizes of 2–10 was performed using the pseudorandom number generator (version 3.0.5) within SMART-TRIAL [53].

Participants were blinded to group allocation and the nature of the two conditions – e.g., both conditions were referred to as “a simple cognitive task intervention” in the informed consent materials. Expectancy/credibility ratings were administered in both conditions. The independent statisticians from Uppsala Clinical Research Center (NH and KG) were blinded to group allocation, scored all measures and performed the analyses. Outcome assessments were self-reported by participants directly into the digital platform (SMART-TRIAL) without interference from researchers (thus researchers were blind to data collection) in most cases, and were occasionally received by post/SMS, based on participant preferences. Owing to the nature of the intervention, the researcher administering the guided session was not blind. The unblinded researchers did not score assessment measures nor analyse results, with the exception of open-ended questions which required coding.

### Treatment conditions

Both arms were administered remotely using a blend of digitalized materials that participants navigated in the secure electronic data collection platform SMART-TRIAL® [54] via their own smartphone. The platform included written instructions and assessments, links to instructional videos (either animated cartoons or of a researcher providing verbal instructions [55]) and external websites (to play Tetris® / listen to the podcast). The initial session (day 1) for both groups was guided by a researcher who was present on a telephone/video call for the full duration to promote adherence, answer questions and provide guidance as necessary. In both arms, the task component of the session took approximately 25 min.

#### Intervention arm

Participants watched video instructions about their task, namely that the simple cognitive task procedure involved three key components: a brief reminder cue to one specific intrusive memory selected from their list of hotspots (hotspot; a short description of the image in just a few words, see below); playing the computer game Tetris® on their smartphone for 20 min; and using ‘mental rotation’ during gameplay, i.e., to imagine how the upcoming differently shaped blocks could be rotated to best fit and create complete lines, one block at a time. They watched an instructional video about the brief memory reminder and were then guided by the researcher to swiftly make a list of their intrusive memories (resulting in a number of hotspots, Mdn = 5, IQR = 3–6) by briefly describing ‘what they see’ in each image (e.g., 'I see the young patient being connected to the ventilator'), but not going into detail about their memory. They then from the list chose one of these hotspot/intrusive memories to do the intervention on in the current session. They also watched an instructional video about mental rotation and gameplay. There were three short task comprehension quizzes (after introduction to intervention, after description of mental rotation, and one after gameplay instructions). After receiving all video instructions, participants were asked to access the computer game by coming out of SMART-TRIAL and using their phone to access www.tetris.com, skip the advertisements, adjust game settings to ‘ghost piece off’, and play uninterrupted for at least 20 min on their own smartphone. A final video gave a neuroscientific rationale of the task, and summarised how to do the intervention steps. They were told they could repeat the intervention to target additional intrusive memories at a later time on their own.

#### Control arm

Participants watched video instructions about their task, which explained that the simple cognitive task procedure involved listening to a radio program on their own smartphone, a podcast about philosophy in Sweden from a popular and well-respected public radio show (Sveriges Radio) called Filosofiska Rummet (The Philosophy Room) [56]. They were instructed to listen uninterrupted and focus on the ideas and meaning in the podcast. Participants were guided by the researcher and given an overview of the various steps in the task**.** There were two short task comprehension quizzes (after introduction to task and after the listening task). After receiving all video instructions about the task, participants were asked to access the podcast by coming out of SMART-TRIAL and using their phone to access it. Participants received a link and were asked to click on it to access the podcast via www.sverigesradio.se/avsnitt/1073596. They were told to listen uninterrupted for at least 20 min on their own smartphone and to focus on what was being said. They rated their compliance with the task and were told that they could continue listening to the remainder of the podcast at a later time on their own.

In both conditions, participants used their own smartphone, and had options to engage in self-administered/guided booster sessions, and completed distress ratings. They continued to have access to the computer game or podcast on their own smartphone. However, after the guided session they did not have the option to go back to the digital platform SMART-TRIAL [54], nor to watch the instruction videos again.

### Training in treatment arms

Research staff training included how to administer the intervention and control procedures, and how to explain the daily intrusive memory diary. Clinical supervision was provided by the principal investigator (EH) and/or a clinician researcher experienced in delivering the protocol (MK). Training included a PowerPoint session, a four-session online course, role-play with peers/trainers, and in vivo observation during participant sessions with corrective/ reflective feedback. For the intervention, competency assessments were conducted using ratings about the intervention in a structure adapted from the Cognitive Therapy Scale-revised [57]. During data collection, individual supervision in vivo or soon after a session was provided by EH and/or MK as needed. A fortnightly group supervision was led by EH and LS.

### Assessments

All assessments were completed by participants directly and remotely (via SMART-TRIAL, or occasionally by post/SMS, based on participant preferences).

Participants completed questions on demographics, work and employment details, work related and non-work related traumatic events, and clinical background. Prior psychological trauma was assessed using the Life Events Checklist for DSM-5 (LEC-5 [58]).

### Primary outcome

The primary outcome measure was the number of intrusive memories of traumatic event(s) reported in the diary daily during week 5 (day 29 to 35 post-intervention). Each diary-week had 26 data collection points (two on day 1, then four per day for days 2–7). Therefore, during each week, participants received four links per day (via SMS and/or email) to record the number of intrusions they experienced in the morning, afternoon, evening and night, respectively, directly into SMART-TRIAL (platform for the digital diary). Each time, participants were asked ‘how many intrusive memories did you have during the morning/afternoon/evening/night’ with a list of 9 possible responses (i.e., ‘0’, ‘1’, ‘2’, ‘3’, ‘4’, ‘5’, ‘6’, ‘7’, ‘more than 7’). If ‘more than 7’, the participant could enter the number manually. Each link also included a brief definition of intrusive memories, and instructions about how to monitor them as follows: ‘Intrusive memories are images from a traumatic event that pop suddenly into your mind, when you do not want them to (they are not the same as deliberately choosing to think about the event or thinking about it in words). Please record every intrusive memory you have had—even if it is the same one popping up several times. If you did not have any, please choose 0.’ The vast majority of participants recorded their intrusions daily, while some provided only their total number of intrusive memories at the end of the week *N* = 7/144.

### Secondary outcomes

The number of intrusive memories was also assessed in daily diaries during week 0 and week 1. At week 1, some participants only provided their total number of intrusive memories for the whole week (*N* = 4/144).

At Week 0, 1 and 5, participants also retrospectively estimated the frequency of their intrusive memories in the previous week using a single item from the Intrusion Questionnaire (IQ) [59], in order to explore convergence with the diary (this was high, see Results). The remaining five items on the IQ assessed characteristics of intrusive memories (i.e., intrusion-related distress, nowness, reliving, disconnectedness, and whether triggers were associated with intrusions (see Additional file 1, Table S1 and Table S2).

PTSD symptoms during the past month were assessed with the 8-item version of the Post-traumatic Stress Disorder Checklist 5 (PCL-5) [60], with a scale ranging from 0–32.

Posttraumatic distress was indexed by the intrusion and avoidance subscales of the Impact of Event Scale–Revised (IES-R), with the sum score of each scale ranging from 0–32 [61].

In addition to the IQ [59], self-reported characteristics of intrusive memories included two diary items rating the vividness and associated distress (weeks 1 and 5) (see Additional file 1, Table S1 and Table S2).

### Other pre-specified outcomes and additional assessments including functioning

The burnout subscale (9 items) of the Scale of Work Engagement and Burnout (SWEBO) [62, 63] assessed symptoms of exhaustion, disengagement and inattentiveness.

The Swedish version of the World Health Organization Disability Assessment Schedule (WHODAS 2.0, 12 items) [64] measured functioning in six life domains: 1) cognition, 2) mobility, 3) personal care, 4) relations, 5) daily activities, and 6) participation in society.

Stress was assessed using the stress subscale (3 items) of the Stress Energy Questionnaire (SEQ) [65, 66].

Perceived health was determined using the Self-Rated Health rating (SRHR) [67].

Sleep was assessed using the Sleep Condition Indicator (SCI-02, 2 items) [68, 69].

Concentration disruption associated with intrusive memories was measured using ratings of level and duration of disruption [29].

Concentration and memory difficulties more broadly were assessed with 11 items [70, 71], with higher ratings indicating lower difficulties.

For measures of sick leave, self-rated functioning, difficulties in letting go of work-related thoughts, social support ratings, moral stress, work situation, coping, see Additional file 1: Other pre-specified outcomes and additional assessments – Functioning [25, 72, 73].

### Other cognitive assessments

For measures of appraisals of intrusions, time perspective questionnaire, future self-identity, see Additional file 1: Other pre-specified outcomes and additional assessments- Other cognitive assessments [74–78].

### Assessments related to procedures

Adverse events (AEs) were assessed throughout the trial by a free response item in which participants indicated whether they had experienced any health issues since their last contact with the study team. Information about AEs was acquired throughout the study via scheduled questions and free report. In total, 183 AEs were reported with 168 (91.8%) reported via the scheduled questions in SMART-TRIAL and 15 (8.2%) to the research team spontaneously (i.e., free report) (Additional file 1, Table S3 and Table S4).

Treatment credibility/expectancy about the assigned intervention/control task was assessed using the Credibility/Expectancy Questionnaire [79] with five items on an 11-point scale (from 0 to 10) (Table 1).

Current level of distress was assessed three times during the task procedure in the guided session (both arms) using Subjective Units of Distress (SUDS) ratings (from 0 “no distress at all” to 10 “worst imaginable distress”) (Additional file 1, Table S5).

A feedback questionnaire (8 bespoke items) about participation was used to assess acceptability and feasibility (e.g., whether participants would recommend the intervention they received to a colleague or friend who had a similar experience, and whether they had done the task on their own).

For additional details see Additional file 1: Other pre-specified outcomes and additional assessments—Assessments related to procedures.

### Procedure

All study procedures were completed remotely by the participants using a smartphone/computer and an electronic data collection platform (SMART-TRIAL [54]). Potential participants expressed interest in the study by providing their contact details to the research team via a study-specific email address. A researcher then contacted them by telephone and provided information about the study, determined eligibility for inclusion, gave the opportunity for discussion of the informed consent sheet, answered any questions, and obtained each participant’s digital written or recorded verbal and informed consent.

Participants were next asked to monitor their intrusive memories in a daily diary (baseline, week 0) after receiving information via video about what intrusive memories are and instructions about how to monitor and report them. After completing the week 0 diary for 7 days, and having baseline measures sent to them on the final day of that week, participants were then randomised to one of the two conditions. According to arm assignment, they completed a single session of the intervention or control procedure guided by a researcher who supported them remotely via phone as they accessed the digital platform (day 1). After the researcher-guided session, participants in both arms could use their assigned task in a self-guided manner, although they could no longer access the instructional videos (so this needed to be done from their memory of what they had learned in the session). Participants in the intervention arm were informed that they could contact the researcher in the event that they wanted a booster session. All participants monitored their intrusive memories in a daily diary during week 1 and completed one-week follow-up assessment measures by the end of that week. At week 5, participants completed follow-up questionnaires and again the daily diary (primary outcome). Outcome measures were collected at 1, 3 and 6 month timepoints.

### Power analysis

The total number of enrolled participants needed was estimated at 164 (c. 82 per group) based on a pre-pandemic pilot study in which we obtained diaries at week 5 from 36 out of 42 participants [44]. These diaries were used to calculate the sample needed for the planned randomised controlled trial, mean intervention = 0.278 (SD = 0.575) versus mean control = 2.889 (SD = 6.434). Based on this 2-group between-group difference (equivalent of ~ 0.5 standard deviation units), a power of 90% with two-sided testing and an alpha of 0.05, we required a sample size of 65 randomised participants per group (130 in total). In this COVID-19 adjusted study we took into account the possibility that the pandemic could affect study participation and thus chose a more conservative level for estimating attrition than in the pilot study (i.e., 20% versus 12.2%).

### Statistical analyses

As indicated in the Statistical Analysis Plan, primary analyses were conducted on an intention-to-treat (ITT) basis, defined as all randomised participants. Analyses were also conducted on the per-protocol (PP) analysis set. The intention-to-treat sample comprised 144 participants, using imputed data (118 participants had data for all diary days and 12 partially completed the diary, and 14 participants were missing). The PP sample comprised 127 participants (115 participants had data for all diary days and 12 partially completed the diary, while no participants were missing). The primary endpoint was analysed using Quasi-Poisson models with multiplicative overdispersion parameter to the variance function. Observed medians, means and standard deviations as well as the incidence rate ratio (IRR) (indicating the likelihood of having different (< 1 lower and > 1 higher) number of intrusive memories in the intervention arm compared to the control arm), and 95% confidence intervals (CIs) for IRR were reported. In addition, Pearson correlations were conducted to explore convergence between diary data at weeks 0, 1 and 5, and corresponding IQ ratings.

Multiple imputation (MI) method [80] was used to deal with missing data in the variable number of intrusive memories (see Additional file 1, Table S6). MI was performed for all individual days at Week 0, Week 1 and Week 5 and then added together to get imputed variables, number of intrusive memories recorded at Week 0, Week 1 and Week 5. SAS function PROC MI was used. We created 30 imputed datasets (to ensure that our effect estimates will not overlay inaccurately due to Monte Carlo variability) [80]. Imputation was performed based on MAR (missing at random) assuming that missingness was conditional on demography variables age, gender, occupation, marital status, work type, education and number of prior psychological traumas (LEC-5) and for week 1 and week 5 even conditional on number of intrusive memories at week 0. Note that imputation is not conditional on treatment arms. The results for each imputation were combined using SAS function PROC MIANALYZE.

The PP population was used for primary outcome and sensitivity analyses focused specifically on missing data patterns. The PP analysis set consists of subjects who have undergone the guided intervention session and for whom there are no significant adherence and protocol deviations. Protocol deviations were classified prior to unblinding of treatment arm and were defined as follows:

1. Non-completion of the primary outcome measure (no data at all).
2. Non-completion of guided intervention session/non-adherence to the intervention.

Descriptive data for the primary outcome in text and Table 2 are based on complete diary data ITT *n* = 118 (Control arm = 58 and Intervention arm = 60), PP *n* = 115 (Control arm = 56 and Intervention arm = 59) (not imputed data, which can be found in Additional file 1, Table S7a).

A number of sensitivity analyses were conducted to check the robustness of the treatment effect. Primary outcome was analysed using Negative-Binomial regression models as well as a completer case analysis for participants who reported data for either total number of intrusive memories per day (i.e., for every day of the week) or only total number of intrusive memories at Week 5. Also, number of intrusive memories recorded in Week 5 were compared between treatment arms using a non-parametric Wilcoxon’s rank sum test and models without outliers. A sensitivity analysis in multiple imputation under the missing not at random (MNAR) assumption was also performed.

The secondary outcome—Number of intrusive memories at week 1—was analysed in the same way as the primary outcome, using a Quasi-Poisson regression model adjusted for Number of intrusive memories at baseline. The main result was based on imputed data under MAR, but sensitivity analyses on complete data and imputed data under MNAR were also performed.

The analyses of all other secondary outcomes were done on available data from the ITT population, without covariate adjustment. The secondary outcomes with answers on a scale or sum of scales were considered to be ordinal variables and analysed using proportional odds logistic regression. The proportional odds assumption was inspected visually for the 5-week outcomes by plotting the empirical cumulative distributions on the logit scale stratified by intervention/control group and examining whether the curves seemed parallel. Moreover, Brant tests were performed. No notable deviances from the assumption were observed. The dichotomous outcomes were analysed using binomial logistic regression. Count outcomes were analysed using Quasi-Poisson regression. Nominal outcomes with more than two levels were compared using Chi-squared tests. For ordinal and count outcomes, Cohen’s *d* effect size with 95% confidence interval were also presented. *P*-values were not adjusted for multiplicity. Where coding of freetext responses was done by two raters, Cohen’s kappa was calculated as measure of agreement.

In summary, there are *n* = 144 patients included in ITT population. In the primary outcome variable “Number of intrusive memories at week 5”, there are four groups of patients: (i) Patients who reported data for all days (complete) *n* = 111, (ii) Patients who only reported total number of IM at week 5 (total reported) *n* = 7; (iii) Patients who did not reported all days of the week (not complete) *n* = 12; (iv) Patients with missing data for all days and total number not given (all missing) *n* = 14. Note that for the completer diary analysis (i) and (ii) are combined, thus *n* = 118.

For additional information see the Statistical Analysis Plan.

## Results

**Fig. 1:**
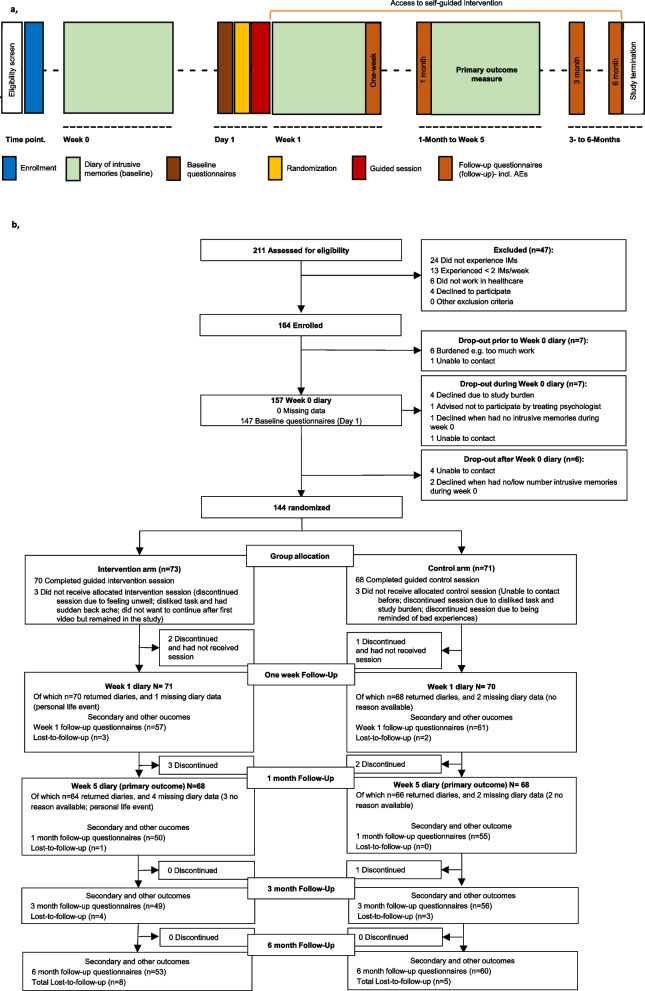
Procedure timeline and participant flow diagram. **a***, Procedure timeline.* After enrolment, participants completed a baseline diary recording number of intrusive memories (week 0) then completed baseline questionnaires. They were randomised to condition prior to the researcher-guided session (control or intervention) on day 1. During the following 7 days, participants completed a second diary (week 1), and completed week 1 follow-up questionnaires on day 8. During the study, participants could use the intervention self-guided (orange horizontal line). After completing the 1-month follow-up questionnaires, participants again completed the 7-day diary (week 5, primary outcome). Follow-up questionnaires were administered at 3 and 6 months. Total study duration was 176 days. AEs, adverse events. **b**, *Participant flow (CONSORT diagram)* indicating participant numbers throughout the course of the trial. Missing data refers to instances when a participant had no reported entries for that timepoint. IMs, intrusive memories. Lost-to-follow-up refers to participants who did not complete follow-up questionnaires up to 6 months

Two hundred and eleven participants were assessed for eligibility; of these, 164 were enrolled from 30 September 2020 to 27 April 2022 (final data were obtained on 31 October 2022). A total of 144 participants were randomised to the intervention (*n* = 73) and control (*n* = 71) arms (Fig. 1). The full analysis set consisted of all randomised participants as the ITT population (*n* = 144). During the guided session, 70 participants completed all components in the intervention arm, and 68 completed all components in the control arm. In total, 130 participants completed the whole study [control arm, *n* = 66 (93.0%); intervention arm, *n* = 64 (87.7%)], with 8 discontinuing before the primary endpoint. The primary outcome of the number of intrusive memories of traumatic event(s) was recorded in a daily diary for 7 days, at week 5. Of these, 118 participants completed data for all seven diary days and 12 completed partial diary data. The PP sample comprised 127 participants. Overall, 13 participants were lost to follow-up (control arm, *n* = 5; intervention arm, *n* = 8) and 9 discontinued (control arm, *n* = 4; intervention arm, *n* = 5). Thus overall (*n* = 144) there was an attrition rate of 6.3%.

### Baseline characteristics, traumatic events, post-traumatic stress and expectancy

Baseline characteristics were comparable between arms (Table 1). The mean (s.d.) age of the total sample was 41.41 ± 10.89 years. The majority of participants identified as female (81.9%), were full-time employees (70.8%) and worked as a nurse (58.3%).

**Table 1:** Baseline characteristics including demographics, traumatic events and expectancy ratings

	**Statistics**	Control *n* = 71	Intervention *n* = 73	Total *n* = 144
**Demographics**				
Age (years)	n	71	73	144
	Mean (s.d.)	42.03 (10.18)	40.81 (11.57)	41.41 (10.89)
	Median (Q1-Q3)	42.0 (35.0–49.0)	41.0 (30.0–51.0)	41.0 (32.0–50.0)
	Min–Max	23.0–64.0	24.0–65.0	23.0–65.0
Gender				
Female	n(%)	59 (83.1)	59 (80.8)	118 (81.9)
Male	n(%)	11 (15.5)	14 (19.2)	25 (17.4)
Other	n(%)	1 (1.4)		1 (0.7)
Ethnicity				
Nordic	n(%)	58 (81.7)	57 (78.1)	115 (79.9)
Nordic/South American	n(%)		1 (1.4)	1 (0.7)
Nordic/Asian	n(%)		2 (2.7)	2 (1.4)
European	n(%)	8 (11.3)	2 (2.7)	10 (6.9)
Middle Eastern	n(%)	1 (1.4)	1 (1.4)	2 (1.4)
South American	n(%)		3 (4.1)	3 (2.1)
Asian	n(%)		1 (1.4)	1 (0.7)
Not reported	n(%)	4 (5.6)	6 (8.2)	10 (6.9)
**Work and Employment**				
Occupation in healthcare				
Nurse	n(%)	40 (56.3)	44 (60.3)	84 (58.3)
Doctor	n(%)	6 (8.5)	3 (4.1)	9 (6.3)
Ambulance	n(%)	1 (1.4)	3 (4.1)	4 (2.8)
Physical therapist	n(%)	2 (2.8)		2 (1.4)
Assistant nurse	n(%)	19 (26.8)	22 (30.1)	41 (28.5)
Other	n(%)	3 (4.2)	1 (1.4)	4 (2.8)
Questions related to work situation				
Type of healthcare place currently working in				
Emergency care (e.g., ICU, Anaesthetics, Ambulance)	n(%)	37 (52.1)	36 (49.3)	73 (50.7)
Other care (e.g., outpatients care, home nursing, geriatric ward)	n(%)	23 (32.4)	18 (24.7)	41 (28.5)
Other	n(%)	11 (15.5)	19 (26.0)	30 (20.8)
Other comments about workplace				
Time working in healthcare (years)	n	71	73	144
	Mean (s.d.)	16.44 (10.77)	15.27 (10.95)	15.85 (10.84)
	Median (Q1-Q3)	15.0 (8.0–24.0)	13.0 (6.0–25.0)	14.0 (7.0–24.5)
	Min–Max	0.0–44.0	0.0–40.0	0.0–44.0
Employment status				
Full-time employed	n(%)	49 (69.0)	53 (72.6)	102 (70.8)
Part-time employed	n(%)	15 (21.1)	9 (12.3)	24 (16.7)
Unemployed	n(%)		1 (1.4)	1 (0.7)
Student	n(%)	2 (2.8)	4 (5.5)	6 (4.2)
On sick leave	n(%)	5 (7.0)	3 (4.1)	8 (5.6)
Other	n(%)		3 (4.1)	3 (2.1)
**Traumatic events**				
Number of work-related traumatic events during the COVID-19 pandemic	n	59	60	119
	Mean (s.d.)	15.92 (19.58)	18.10 (23.12)	17.02 (21.38)
	Median (Q1-Q3)	10.0 (5.0–20.0)	10.0 (5.0–20.0)	10.0 (5.0–20.0)
	Min–Max	1.0–100.0	0.0–100.0	0.0–100.0
Type of trauma(s) leading to intrusive memories				
A traumatic or tragic death of a patient	n(%)	57 (80.3)	56 (76.7)	113 (78.5)
A severe or unsuccessful resuscitation	n(%)	15 (21.1)	18 (24.7)	33 (22.9)
Witnessing events surrounding colleague who has fallen ill or died of COVID-19	n(%)	11 (15.5)	15 (20.5)	26 (18.1)
Situation where the care of a patient failed or did not go as planned	n(%)	46 (64.8)	51 (69.9)	97 (67.4)
Threats or violence against healthcare professionals	n(%)	12 (16.9)	14 (19.2)	26 (18.1)
Event involving sudden increased risk of COVID-19 infection	n(%)	36 (50.7)	32 (43.8)	68 (47.2)
A traumatic or tragic event where a patient reminded you of yourself, a family member or friend	n(%)	26 (36.6)	33 (45.2)	59 (41.0)
Event involving extremely distressed/grieving relatives of patients, a family member or friend	n(%)	41 (57.7)	46 (63.0)	87 (60.4)
Being faced with suicide/suicide attempt of a family member or friend	n(%)	5 (7.0)	7 (9.6)	12 (8.3)
Other (yes/no)^a^	n(%)	12 (16.9)	16 (21.9)	28 (19.4)
Total	n(%)	71 (100.0)	73 (100.0)	144 (100.0)
Time since traumatic event(s) leading to intrusive memories				
Within 24 h	n(%)	9 (12.7)	4 (5.5)	13 (9.0)
Within a month	n(%)	8 (11.3)	6 (8.2)	14 (9.7)
More than 1 month ago	n(%)	12 (16.9)	14 (19.2)	26 (18.1)
Between 1–3 months ago	n(%)	48 (67.6)	56 (76.7)	104 (72.2)
More than 3 months ago	n(%)	30 (42.3)	33 (45.2)	63 (43.8)
Number of non-work-related traumatic events during the COVID-19 pandemic	n	59	60	119
	Mean (s.d.)	6.03 (32.51)	12.27 (43.71)	9.18 (38.53)
	Median (Q1-Q3)	0.0 (0.0–2.0)	0.5 (0.0–3.0)	0.0 (0.0–3.0)
	Min–Max	0.0–250.0	0.0–300.0	0.0–300.0
Number of prior psychological traumas (LEC-5)^b^	n	71	73	144
	Mean (s.d.)	6.13 (4.05)	7.63 (5.78)	6.89 (5.04)
	Median (Q1-Q3)	6.0 (3.0–9.0)	6.0 (4.0–10.0)	6.0 (3.0–9.5)
	Min–Max	0.0–20.0	1.0–30.0	0.0–30.0
**Number of intrusive memories Week 0**	n	71	73	144
	Mean (s.d.)	19.48 (16.50)	21.79 (21.32)	20.65 (19.06)
	Median (Q1-Q3)	14.0 (7.0–28.0)	16.0 (6.0–29.0)	15.0 (6.25–28.75)
	Min–Max	0.0–73.0	2.0–137.0	0.0–137.0
**Clinical background**				
Sleep ratings (SCI-02) (0–8)	n	71	73	144
	Mean (s.d.)	4.14 (2.63)	3.97 (2.53)	4.06 (2.57)
	Median (Q1-Q3)	4.0 (2.0–7.0)	4.0 (2.0–6.0)	4.0 (2.0–6.0)
	Min–Max	0.0–8.0	0.0–8.0	0.0–8.0
**Expectancy of intervention effect (Day 1)**				
Credibility/expectancy questionnaire (**total score**)	n	69	73	142
	Mean (s.d.)	24.67 (8.91)	20.21 (11.57)	22.37 (10.56)
	Median (Q1-Q3)	25.0 (19.0–30.0)	19.0 (11.0–28.0)	23.0 (15.0–29.0)
	Min–Max	5.0–49.0	0.0–48.0	0.0–49.0
**Individual items** of credibility/expectancy^c^				
Right now, how logical do you think the administered task seems?	n	69	73	142
	Mean (s.d.)	5.41 (2.44)	4.71 (2.88)	5.05 (2.69)
	Median (Q1-Q3)	5.0 (4.0–7.0)	5.0 (3.0–6.0)	5.0 (3.0–7.0)
	Min–Max	0.0–10.0	0.0–10.0	0.0–10.0
Right now, how successful do you think that this task will be in preventing/reducing your intrusive memories?	n	69	73	142
	Mean (s.d.)	4.83 (1.92)	3.88 (2.41)	4.34 (2.23)
	Median (Q1-Q3)	5.0 (4.0–6.0)	4.0 (2.0–5.0)	5.0 (3.0–5.0)
	Min–Max	0.0–10.0	0.0–10.0	0.0–10.0
With what degree of trust would you recommend this task to a friend experiencing similar problems?	n	69	73	142
	Mean (s.d.)	5.33 (2.44)	4.45 (2.92)	4.88 (2.72)
	Median (Q1-Q3)	5.0 (4.0–7.0)	5.0 (2.0–6.0)	5.0 (3.0–6.0)
	Min–Max	0.0–10.0	0.0–10.0	0.0–10.0
Right now, how much improvement regarding your intrusive memories do you think you will experience after completing the task?	n	69	73	142
	Mean (s.d.)	4.94 (1.92)	3.77 (2.50)	4.34 (2.31)
	Median (Q1-Q3)	5.0 (4.0–6.0)	4.0 (2.0–5.0)	5.0 (3.0–6.0)
	Min–Max	0.0–9.0	0.0–10.0	0.0–10.0
Right now, how much does it feel like this task is going to reduce the number of intrusive memories for you?	n	69	73	142
	Mean (s.d.)	4.16 (2.22)	3.40 (2.52)	3.77 (2.40)
	Median (Q1-Q3)	5.0 (2.0–5.0)	3.0 (1.0–5.0)	4.0 (2.0–5.0)
	Min–Max	0.0–10.0	0.0–10.0	0.0–10.0

Data are displayed for the ITT sample. Day 1 questionnaires at baseline are pre-randomisation

^a^Content of the trauma categories ‘other’ is presented in Additional file 1, Table S8

^b^Prior psychological trauma per category (LEC-5) is presented in Additional file 1, Table S9

^c^The five items on the Credibility/Expectancy Questionnaire were rated on an 11-point scale (from 0 to 10)

The mean number of work-related traumatic events during the pandemic at baseline was 17.02 (± 21.38), and the majority (72.2%) were experienced in the previous 1–3 months. In addition, participants reported an average of 9.18 ± 28.53 non-work-related traumatic events during the pandemic. The number of prior psychological traumas was indexed using LEC-5 [58] (Table 1).

The mean (s.d.) post-traumatic stress symptoms score at baseline was 12.10 ± 6.63 (scale ranging from 0–32, with cut-off for clinical importance typically set at 19; (PTSD Checklist for DSM-5, 8 item version, PCL-5) [60]. The post-traumatic distress score was 15.29 ± 6.07 and 13.72 ± 6.85 for intrusion and avoidance subscales, respectively (sum score of each scale ranging from 0–32 using the IES-R) [61]. Eighty participants (55.6%) reported a current or past mental health condition (Additional file 1, Table S1).

At baseline (week 0), the median number of intrusive memories recorded in a daily diary for one week was similar between arms (combined median = 15.00; IQR = 7.5–28.5, *n* = 118) (Additional file 1, Table S7a (complete diary data) and S7b (incomplete diary), day-by-day Additional file 1, Fig. S1A).

At baseline (day 1, after randomisation and prior to intervention), participants’ expectations that their assigned task would help reduce intrusive memories were low (credibility scale maximum = 54) and differed between arms, with lower credibility/expectancy ratings in the intervention group (mean ± s.d.: control: 24.67 ± 8.91, *n* = 69; intervention: 20.21 ± 11.57, *n* = 73; OR = 0.45, (95% CI = 0.25–0.80),* P* = 0.0072], (Table 1**)**. See Table 1 and Additional file 1, Table S1 and Tables S7 to S10 for further baseline data.

### Primary outcome

#### Number of intrusive memories of traumatic events (week 5)

The primary outcome was the Number of intrusive memories recorded in a daily diary during week 5 after the intervention/control task. The pre-specified primary analysis was ITT (i.e., *n* = 71 control arm; *n* = 73 intervention arm). Sixty-six participants in the control arm and 64 in the intervention arm returned the daily diary at week 5 (Fig. 1). Six participants did not adhere to the task (control arm: *n* = 3; intervention arm: *n* = 3) (Table 2)

**Table 2:** Primary outcome by condition: Number of intrusive memories of traumatic events at week 5

	Observed Median (IQR)		Observed Mean (s.d.)			
Population	**Control**	**Intervention**	**Control**	**Intervention**	Estimated IRR (95% CI)^a, d^	*p*-value
ITT^b^						
*N* = 144	5.0 (1–17)	1.0 (0–3)	12.4 (17.5)	3.4 (8.6)	0.30 (0.17–0.53)	< 0.0001
PP^c^						
*N* = 127	6.0 (1–18)	1.0 (0–3)	12.8 (17.7)	3.5 (8.7)	0.29 (0.16–0.53)	< 0.0001

The baseline number of intrusive memories (at Days -7 to -1) has been included as a covariate

^a^The reference group is the Control Group. *ITT* intention-to-treat sample, *PP* per protocol sample, *IQR* inter quartile range, *s.d.* standard deviation, *IRR* Incidence Rate Ratio

^b^ITT Control arm (*n* = 71), ITT Intervention arm (*n* = 73)

^c^PP Control arm (*n* = 64), PP Intervention arm (*n* = 63)

^d^ITT analysis use imputed data. In this table descriptive data are based on complete diary data ITT *n* = 118 (Control arm = 58 and Intervention arm = 60), PP *n* = 115 (Control arm = 56 and Intervention arm = 59) (not imputed data, which can be found in Additional file 1, Table S7a)

**Fig. 2:**
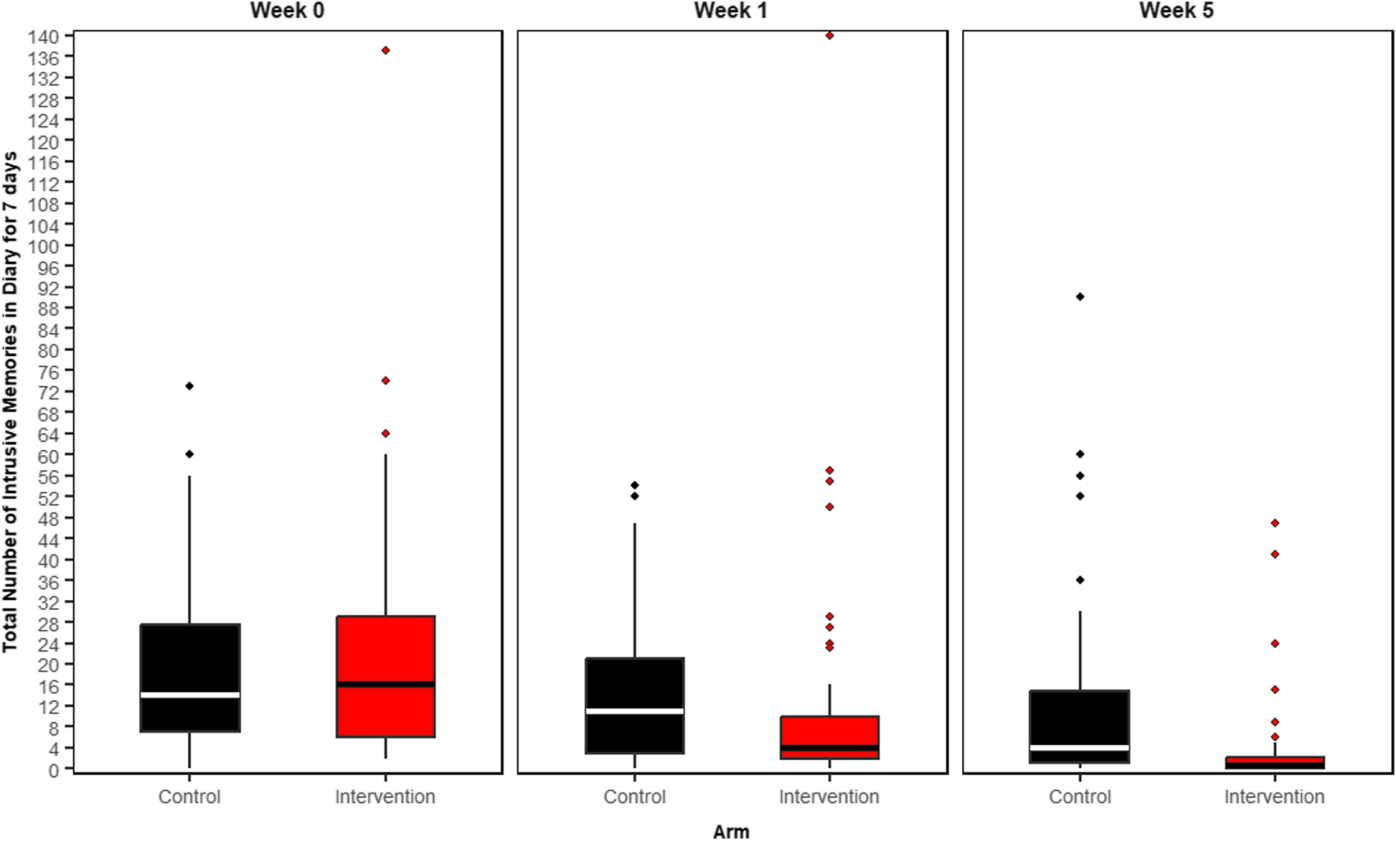
Number of intrusive memories of work-related traumatic events per condition at each of three time points: week 0 (pre-intervention baseline), week 1 (immediately post-intervention) and over week 5 (primary outcome) Boxplots show number of intrusive memories of traumatic events, whereby the midline is the median value. Upper and lower box limits are the third and first quartile (75th and 25th percentile), with the whiskers covering 1.5 times the interquartile range (IQR). All outliers are included in this figure and shown as dots (each dot represents one participant that departed by more than 1.5 times the IQR above the third quartile and below the first quartile). Number of intrusive memories of traumatic events are recorded by participants in a brief daily online intrusive memory diary for 7 days (daily diary) The figure is based on diary data available in the ITT sample, including incomplete diary data, for week 0: *n* = 144, week 1: *n* = 136, and week 5: *n* = 130 The imagery-competing task intervention consisted of a cognitive task involving a brief memory cue plus Tetris® computer gameplay with mental rotation instructions, with a first guided session with the researcher The active control (attention-based placebo comparator) consisted of a cognitive task that also involved a digital activity and, for the same amount of time, listening to a podcast on philosophy, with a first guided session with the researcher ** Week 0: Baseline measure.** Number of intrusive memories in the daily diary during the baseline week for both arms (black = control arm, *n* = 71: attention-based placebo control; red = intervention arm, *n* = 73: remotely-delivered, imagery-competing task intervention) showing that the two arms did not differ at baseline (i.e., before the intervention was provided to either arm) ** Week 1: Secondary outcome measure.** Number of intrusive memories in the daily diary during week 1 for each arm (black = control arm, *n* = 67: attention-based placebo control; red = intervention arm, *n* = 69: remotely-delivered, imagery-competing task intervention). The intervention arm had fewer intrusive memories at week 1 compared to the control arm ** Week 5: Primary outcome measure.** Number of intrusive memories of traumatic events recorded by participants in a brief daily online intrusive memory diary for 7 days during week 5 for both arms (black = control arm, *n* = 66: attention-based placebo control; red = intervention arm, *n* = 64: remotely-delivered, imagery-competing task intervention). The intervention arm had fewer intrusive memories at week 5 compared to the control arm

Participants in the intervention arm reported significantly fewer intrusive memories at week 5 than did those in the control arm using the ITT sample (summary of the outcome per arm: control arm Mdn = 5.0 (IQR = 1–17), *n* = 58; intervention arm Mdn = 1.0 (IQR = 0–3), *n* = 60; IRR based on imputed data = 0.30 (95% CI = 0.17–0.53); *p* < 0.0001 (Table 2, Fig. 2 and for day-by-day Additional file 1, Fig. S1C). The baseline number of intrusive memories (at days -7 to -1) has been included as a covariate.

Sensitivity analyses evaluating the robustness of treatment effect showed this difference remained significant using a pre-specified PP population (Table 2). Additional sensitivity analyses showed that using a non-parametric Wilcoxon’s test (Additional file 1, Table S11) and with imputed data using MAR (Additional file 1, Table S12) and using missing not at random (MNAR) assumption (Additional file 1, Table S13), and exclusion of outliers (Additional file 1, Table S14) as well as excluding diary missing data (completers only), the pattern of results remained (Additional file 1, Table S15).

Gender and age-distributed descriptive data regarding the primary outcome (Additional file 1, Tables S16 and S17) appeared comparable for women and men and across age levels.

### Secondary outcomes

#### Number of intrusive memories of traumatic events (week 1)

Participants in the intervention arm reported significantly fewer intrusive memories of traumatic events in the daily diary during the week after the guided session (week 1) than did those in the control arm [control arm Mdn = 11 (IQR = 3–21), *n* = 65; intervention arm Mdn = 4.5 (IQR = 2–10.5), *n* = 64; IRR based on imputed data = 0.53 (95% CI = 0.41–0.70); *p* < 0.0001] (Fig. 2, Additional file 1, Table S12, day-by-day Fig. S1B).

At the end of each week 0 and 1 and start of week 5, participants completed an intrusion diary. They also provided a retrospective rating of their intrusive memory frequency during the previous week (intrusion questionnaire; IQ [59]) (Additional file 1, Table S2). Correlations were calculated to explore convergence between diary data at weeks 0, 1 and 5, and corresponding IQ ratings. This showed that the total number of intrusive memories reported in the diary and IQ ratings were significantly correlated at all timepoints (baseline: *r* = 0.745, *p* < 0.0001, week 1: *r* = 0.793, *p* < 0.0001, week 5: *r* = 0.651, *p* < 0.0001).

### Clinical symptoms

#### Post-traumatic stress symptoms and post-traumatic distress

Relative to the control arm, participants in the intervention arm reported significantly less post-traumatic stress symptoms (PCL-5 short version 8-item scale) at each timepoint from week 1 through to the 6 month follow-up [week 1 mean ± s.d: control: 10.57 ± 6.91, *n* = 61; intervention: 6.91 ± 6.15, *n* = 54; OR = 0.37 (95% CI = 0.19–0.71), *p* < 0.0031, 1 month mean ± s.d.: control: 10.38 ± 7.30, *n* = 55; intervention: 4.90 ± 5.29, *n* = 51; OR = 0.21 (95% CI = 0.10–0.42), *p* < 0.0001, 3 months mean ± s.d.: control: 8.98 ± 6.92, *n* = 55; intervention: 3.81 ± 5.17, *n* = 47; OR = 0.19 (95% CI = 0.09–0.40), *p* < 0.0001, 6 months mean ± s.d.: control: 8.36 ± 6.47, *n* = 58; intervention: 3.46 ± 4.83, *n* = 52; OR = 0.19 (95% CI = 0.09–0.39), *p* < 0.0001] (Additional file 1, Table S2).

Participants in the intervention arm reported lower post-traumatic distress related to intrusions than those in the control arm at each timepoint from week 1 post-intervention to the 6 month follow-up (IES-R intrusions subscale sum score) [week 1 mean ± s.d.: control: 13.44 ± 6.59, *n* = 61; intervention: 8.36 ± 6.01, *n* = 56; OR = 0.24 (95% CI = 0.12–0.49), *p* < 0.0001, 1 month mean ± s.d.: control: 11.63 ± 7.44, *n* = 56; intervention: 5.25 ± 5.51, *n* = 51; OR = 0.16 (95% CI = 0.08–0.34), *p* < 0.0001, 3 months mean ± s.d.: control: 9.52 ± 6.55, *n* = 56; intervention: 4.18 ± 5.16, *n* = 49; OR = 0.17 (95% CI = 0.08–0.34), *p* < 0.0001, 6 months mean ± s.d.: control: 8.87 ± 6.74, *n* = 60; intervention: 4.36 ± 5.00, *n* = 53; OR = 0.25 (95% CI = 0.12–0.49), *p* < 0.0001]. Avoidance scores in the intervention arm were lower at each timepoint from 1 to 6 months (IES-R avoidance subscale) [week 1 mean ± s.d.: control: 11.48 ± 6.83, *n* = 61; intervention: 10.43 ± 7.15, *n* = 56; OR = 0.73 (95% CI = 0.39–1.38), *p* = 0.34, 1 month mean ± s.d.: control: 10.79 ± 7.42, *n* = 56; intervention: 6.06 ± 7.06, *n* = 51; OR = 0.25 (95% CI = 0.12–0.51), *p* = 0.0002, 3 months mean ± s.d.: control: 9.14 ± 6.75, *n* = 56; intervention: 4.96 ± 6.48, *n* = 49; OR = 0.26 (95% CI = 0.12–0.52), *p* = 0.0002, 6 months mean ± s.d.: control: 7.75 ± 6.80, *n* = 60; intervention: 4.77 ± 5.74, *n* = 53; OR = 0.41 (95% CI = 0.21–0.79), *p* = 0.0082] (Additional file 1, Table S2).

Self-reported characteristics of intrusive memories can be found in Additional file 1, Table S2 and Table S1 for baseline.

#### Other outcome measures including functioning

Work engagement and burnout (SWEBO at 6 months) differed between arms, whereby participants in the intervention arm reported a lower total burnout score, with lower scores on two subscales [SWEBO: total mean ± s.d.: control: 1.80 ± 1.56, *n* = 58; intervention: 1.56 ± 0.58, *n* = 50; OR = 0.46 (95% CI = 0.23–0.90), *p* = 0.0240; disengagement subscale mean ± s.d.: control: 1.66 ± 0.66, *n* = 58; intervention: 1.42 ± 0.60, *n* = 50; OR = 0.45 (95% CI = 0.22–0.90), *p* = 0.0245; inattentiveness subscale mean ± s.d.: control: 1.78 ± 0.65, *n* = 58; intervention: 1.49 ± 0.40, *n* = 50; OR = 0.40 (0.20–0.80), *p* = 0.0102] (Additional file 1, Table S2). There was no difference in the amount of sick leave between arms (Additional file 1, Table S2).

Participants in the intervention arm reported lower stress, pressure and tenseness at work (SEQ – stress subscale) at 1 month [mean ± s.d.: control: 6.42 ± 3.53, *n* = 55; intervention: 4.25 ± 2.69, *n* = 48; OR = 0.33 (95% CI = 0.16–0.66), *p* = 0.0018] with no difference at other timepoints (Additional file 1, Table S2**)**. Moral stress at work was significantly lower (i.e., higher ratings) in the intervention relative to control arm at 1 month only [mean ± s.d.: control: 13.44 ± 3.81, *n* = 55; intervention: 15.21 ± 3.11, *n* = 48; OR = 2.28 (95% CI = 1.15–4.57), *p* = 0.0189] (Additional file 1, Table S2).

General functioning (WHODAS at 6 months) was better in the intervention compared to the control arm in the domains of cognition [mean ± s.d.: control: 3.59 ± 1.55, *n* = 58; intervention: 3.16 ± 1.71, *n* = 50; OR = 0.48 (95% CI = 0.23–0.96), *p* = 0.0400] and personal care [mean ± s.d.: control: 2.55 ± 1.16, *n* = 58; intervention: 2.20 ± 0.81, *n* = 50; OR = 0.30 (95% CI = 0.08–0.91), *p* = 0.0469]. There was no significant difference between arms for the domains of mobility, relations, daily activities, participation in society or disability score. Frequency of difficulties within a week were lower in the intervention arm compared to the control arm [mean ± s.d.: control: 1.97 ± 2.41, *n* = 58; intervention: 0.88 ± 1.85, *n* = 50; OR = 0.31 (95% CI = 0.14–0.66), *p* = 0.0027], with no difference regarding the impact of difficulties (Additional file 1, Table S2).

Self-rated health (SRHR) was better (i.e., higher ratings) in the intervention arm at all timepoints [week 1 mean ± s.d.: control: 3.59 ± 1.05, *n* = 60; intervention: 4.44 ± 1.18, *n* = 54; OR = 2.61 (95% CI = 1.32–5.26), *p* = 0.0066; 1 month mean ± s.d.: control: 3.89 ± 1.52, *n* = 55; intervention: 4.68 ± 1.19, *n* = 50; OR = 2.84 (95% CI = 1.41–5.87), *p* = 0.0041; 3 months mean ± s.d.: control: 4.11 ± 1.07, *n* = 53; intervention: 4.67 ± 1.17, *n* = 46; OR = 3.13 (95% CI = 1.48–6.80), *p* = 0.0033; 6 months mean ± s.d.: control: 4.10. ± 1.17, *n* = 58; intervention: 4.67 ± 1.22, *n* = 52; OR = 2.82 (95% CI = 1.40–5.82), *p* = 0.0041] (Additional file 1, Table S2).

Self-rated sleep ratings (SCI–02) were higher (indicating better sleep) in the intervention arm relative to the control arm from week 1 to 3 months [week 1 mean ± s.d.: control: 4.92 ± 2.30, *n* = 60; intervention: 5.89 ± 2.45, *n* = 54; OR = 2.61 (95% CI = 1.34–5.17), *p* = 0.0053; 1 month mean ± s.d.: control: 4.65 ± 2.82, *n* = 55; intervention: 6.02 ± 2.10, *n* = 50; OR = 2.16 (95% CI = 1.09–4.33), *p* = 0.0293; 3 months mean ± s.d.: control: 4.77 ± 2.33, *n* = 53; intervention: 6.28 ± 2.05, *n* = 46; OR = 3.92 (95% CI = 1.88–3.38), *p* = 0.0003] Though at 6 months, there was no significant difference [6 months mean ± s.d.: control: 5.40 ± 2.41, *n* = 58; intervention: 6.23 ± 1.83, *n* = 52; OR = 1.85 (95% CI = 0.95–3.64), *p* = 0.0742] (Additional file 1, Table S2).

On the intrusive memory ratings, participants in the intervention condition reported less concentration disruption due to intrusive memories (assessed by a single rating on a scale from 0–10 [29] in the intrusion diary at weeks 1 and 5) compared to the control arm at both timepoints [week 1 mean ± s.d.: control: 2.77 ± 2.06, *n* = 57; intervention: 1.92 ± 2.23, *n* = 51; OR = 0.40 (95% CI = 0.20–0.79), *p* = 0.0092; week 5 mean ± s.d.: control: 2.67 ± 2.31, *n* = 46; intervention: 1.78 ± 2.26, *n* = 27; OR = 0.39 (95% CI = 0.16–0.91), *p* = 0.0321] (Additional file 1, Table S2).

At 6 months, participants in the intervention arm reported less concentration and memory difficulties (i.e., higher ratings on an 11-item scale [70, 71]) than the control arm [mean ± s.d.: control: 38.17 ± 10.84, *n* = 52; intervention: 44.55 ± 7.93, *n* = 42; OR = 3.36 (95% CI = 1.62–7.14), *p* = 0.0013] (Additional file 1, Table S2**)**.

Participants in the intervention arm rated their intrusive memories as having less impact on occupational functioning [25] at each timepoint from 1 month to the 6 month follow-up relative to the control arm [week 1 mean ± s.d.: control: 2.69 ± 2.74, *n* = 61; intervention: 1.72 ± 1.96, *n* = 54; OR = 0.56 (95% CI = 0.29–1.07), *p* = 0.0816;1 month mean ± s.d.: control: 2.96 ± 2.66, *n* = 55; intervention: 1.20 ± 1.97, *n* = 42; OR = 0.22 (95% CI = 0.10–0.45), *p* < 0.0001; 3 months mean ± s.d.: control: 2.20 ± 2.36, *n* = 55; intervention: 0.87 ± 1.60, *n* = 47; OR = 0.30 (95% CI = 0.14–0.63), *p* = 0.0016; 6 months mean ± s.d.: control: 2.10 ± 2.11, *n* = 58; intervention: 1.06 ± 1.87, *n* = 51; OR = 0.28 (95% CI = 0.13–0.56), *p* = 0.0005] (Additional file 1, Table S2). Similarly at each timepoint, participants in the intervention arm reported that intrusive memories had less impact on daily functioning in other areas than the control arm [week 1 mean ± s.d.: control: 4.03 ± 2.71, *n* = 61; intervention: 2.85 ± 2.10, *n* = 54; OR = 0.47 (95% CI = 0.24–0.90), *p* = 0.0243; 1 month mean ± s.d.: control: 3.91 ± 2.41, *n* = 55; intervention: 2.42 ± 2.22, *n* = 50; OR = 0.24 (95% CI = 0.11–0.49), *p* = 0.0001; 3 months mean ± s.d.: control: 3.51 ± 2.46, *n* = 55; intervention: 1.89 ± 1.66, *n* = 47; OR = 0.23 (95% CI = 0.11–0.49), *p* = 0.0002; 6 months mean ± s.d.: control: 3.17 ± 2.19, *n* = 58; intervention: 2.02 ± 1.87, *n* = 51; OR = 0.26 (95% CI = 0.12–0.53), *p* = 0.0003] (Additional file 1, Table S2**)**.

Other measures (including letting go of work-related thoughts, social support, coping, appraisal of intrusions, future self-identity, time perspective questionnaire, work situation) can be found in Additional file 1, Secondary and Other Pre-specified Outcomes Descriptions 2–4 and Additional file 1, Table S2.

### Assessments related to procedures

Feedback on acceptability and feasibility, how upsetting it was to do the task, and subsequent use of task on their own is in Additional file 1, Table S18. Number of hotspots (i.e. different intrusive memories in intervention arm only), days/nights worked during diary completion weeks, booster sessions, and additional traumatic events during the study are in Additional file 1, Table S19.

### Safety

Number of adverse events (AEs) are reported in Additional file 1, Table S3 and types of AEs in Additional file 1, Table S4. In total, 183 AEs were reported during the 6 months. Overall, 74 (51.4%) participants reported at least one AE [36 (49.3%) in intervention arm and 38 (53.5%) in control arm, *p* = 0.6136]. There were no serious adverse events (SAEs). Three AEs were assessed as severe (cancer treatment; burnout; PTSD), of which none were study-related, 49 were assessed as moderate and 131 as mild (Additional file 1, Table S3**)**. Control participants reported more AEs (*n* = 119) than those in the intervention arm (*n* = 64), *p* = 0.0052.

## Discussion

In this parallel-group, two-arm randomised controlled trial, healthcare workers exposed to work-related traumatic events during the COVID-19 pandemic who used a brief behavioural intervention had significantly fewer intrusive memories of trauma at week 5 post-intervention (primary outcome) relative to those in an active control condition. The treatment effect appeared robust given sensitivity analyses across various statistical scenarios. Participants in the intervention arm also had fewer intrusive memories in the first week after the guided intervention session, compared to controls. Further, they had less severe related symptoms of post-traumatic stress over the next 6 months (PCL-5 all time points) with less associated distress (IES-R intrusions all time points, avoidance from 1 month). At six months, they showed better functioning at work (burnout score on SWEBO, less concentration and memory difficulties) and better general functioning (WHODAS). This is the first time such wide-ranging positive effects of this intervention have been demonstrated to occur at this extended timepoint of six months, and while the intervention targeted just one symptom, effects were also evident on related symptoms and functioning.

Notably, the imagery competing task intervention (ICTI) involved only one guided session with a researcher (day 1) within which the intervention took approximately half an hour, 20 min of which involved computer gameplay. The attention-based placebo active control likewise involved one researcher-guided session of similar duration, which included listening to a podcast and was delivered via the same digital platform, and using participants’ own smartphones. We included a subclinical-to-clinical sample (with at least 2 intrusive memories of trauma per week), taking a preventing-to-treating approach in that the intervention could be used from the day of trauma to months later (see Additional file 1, Table S20 for information on time since traumatic event).

The primary endpoint was met as 5 weeks after the guided session there were around one quarter of intrusive memories in the intervention compared to control arm. We note that these results were obtained despite healthcare workers facing ongoing exposure to trauma in the continuing pandemic. In comparison, treatment trials are typically conducted after the trauma is over, or exclude participants with ongoing trauma exposure [13]. Further, given the high number of traumatic events at baseline, the findings are in the context of multiple rather than single event trauma exposure at trial entry. Together, these features of our sample highlight the effectiveness of the intervention.

Whilst exposure to work-related trauma was a key inclusion criterion, approximately one third of the sample also reported work-unrelated traumatic events during the study (Table 1, Additional file 1, Table S8 and Table S19), as well as a high level of prior psychological trauma such as physical assault (LEC-5). For those who received the intervention (*n*=60), the number of intrusive memories at the primary endpoint (week 5) were reduced by 85.9% (mean difference = 16.8) in frequency compared to baseline and with 50% of participants achieving zero intrusive memories (cases with complete data). That intrusions reduced to a median of one per week at primary endpoint is consistent with the earlier (albeit less well controlled) study with UK intensive care staff during the pandemic [37, 38] (UK study intervention arm; Mdn = 1.0; IQR = 0–3), a population which similarly had multiple traumatic event exposure and a high number of intrusions at baseline. Critically, here we have addressed key limitations of the previous trial through the use of a more stringent control condition and longer follow-up (up to 6 months rather than 4 weeks), which strengthened our findings. Symptom rebound over time is a key concern in psychological treatment development, and future work should examine replication of the sustained effects of this brief intervention approach.

The intervention approach appeared safe. There were no SAEs nor any study-related AEs in the intervention arm, indicating an absence of harms. While approximately half the sample in both arms experienced AEs over the 6 months, control participants reported more AEs than intervention participants. The indication of safety is consistent with previous studies but limited by self-report. Feedback regarding acceptability was positive, with both arms rating their task as easy and stating that they would recommend it to a friend. Notably, these ratings were more favourable overall in the intervention than control arm. Acceptance measures at one month indicated the intervention task was not found to be upsetting, consistent with distress ratings taken during the guided session on day 1. Positive appraisals of the interventions have also been reflected in qualitative interviews with the participants [81].

Compared to existing treatments offered after psychological trauma [13], the application of a widely used computer game in combination with only a brief trauma reminder of just a few words [82] (rather than a requirement to talk about the trauma in more detail) may for some be a more tolerable approach to help reduce the frequency of aversive intrusive memories. The focus on only a single symptom, alongside the requirement of just one guided session (approximately 30 min duration), together make this approach relatively simple and substantially briefer than multiple-session guided digital therapy with a therapist (e.g. [83]), and thus more amenable to scaling.

There are several limitations to this study with implications for the generalisability of our results. Recruitment was purely based on self-report and self-referral data, which may underlie several biases. Further, in line with the scope of the study (targeting only one specific symptom), no objective clinical diagnostic interview was conducted. Accordingly, we do not know how many participants met criteria for psychological disorders (e.g., PTSD, depression), and thus cannot generalize effects to specific groups. Intrusive memories occur in several disorders and more broad effects should be studied in future work. Whilst we sought to minimize the questionnaire battery in order to reduce participant burden, including additional measures would have enabled us to examine potential moderators of efficacy. The study was conducted with healthcare workers during the COVID-19 pandemic in Sweden; future tests of generalizability of the findings in samples from other professions and countries will be informative. Finally, we note that the initial digitization of the intervention occurred rapidly in the context of the escalating pandemic. Future refinements (e.g., having the intervention housed within one digital platform) will potentially aid usability.

The uptake and compliance with the single dose guided intervention session was high and similar between arms (intervention 96%, control 96% see Fig. 1 CONSORT diagram). Critically, this session is the main focus of the trial. However, after the guided session was complete (i.e., during the follow-up period), data suggest greater engagement in the intervention than control arm. Specifically, at one month follow-up, 80.9% of participants in the intervention arm continued using the imagery-competing task on their own, whereas 29.6% of participants in the control arm reported listening to the podcast. As all participants received one single guided session but reported different engagement on their own afterward, we can only speculate about the potential effect-to-dose-ratio after the prescribed session. Of note, the guided session for the intervention condition ended with a summary rationale, whereas the control condition did not. While neither group had ongoing access to the digital platform after the guided session, such summary knowledge will have aided intervention participants to self-treat other intrusive memories, in addition to the memory targeted in the guided session. There were also differences in researcher contact time as the intervention arm received optional booster sessions whereas the control arm did not (nor did they request any).

Interestingly, the treatment credibility/expectancy ratings taken after randomisation were low overall, with a significant effect in favour of the control arm. This reverse placebo pattern suggests that the better outcome in the intervention arm is unlikely to be attributed to participant expectations alone, although future trials should attempt to match for expectancy and understand how this may change after an intervention. There was a decline over time of post-traumatic stress symptoms in the control arm too, consistent with a natural decline in symptoms after trauma exposure. For example, assessed over 15 months, patients admitted to the emergency department revealed three types of symptom trajectories– rapid declining, slow declining and non-remitting [84, 85]. Further, simply tracking and monitoring a mental health symptom can lead to a decline in that specific symptom [86], and it is possible our control group benefitted from monitoring their intrusive memories in the daily diary.

This study was not designed to disentangle the underlying mechanisms of the intervention. Potential candidates include mental imagery [35, 87], given the sensori-perceptual nature of intrusive memories, which are theorized to hijack attention [88], disrupting perceptual processing [89]. We hypothesize that after bringing the memory hotspot (briefly) to mind [82], an imagery-competing task such as Tetris gameplay [40, 90], can weaken the representation and render it less intrusive [91]. Timing parameters for this memory updating procedure have been inspired by work in memory reconsolidation [34, 36, 82]. In future work, advancing mechanistic understanding of treating intrusive memories after trauma will be critical [92, 93]. Existing psychological treatments for trauma and stress-related disorders have focussed on fear extinction learning and cognitive verbal reappraisal models. An alternative means to alter traumatic memories to stop them from intruding may provide a complementary approach, useful for individuals who do not wish to confront details of their traumatic experience as part of treatment. Finally, the digital set up of the current intervention was from an academic content and rapidly developed, such that much further digital refinement is needed for real-world use. For example, it would be advantageous to have an integrated digital platform for the study and measures, rather an having to “hop” between applications for gameplay. In the future, harnessing additional digital technologies could be explored. For example, virtual reality (VR) may hold promise, with accumulating positive evidence for immersive PTSD treatments using VR to increase the patient’s sense of presence [94, 95].

Together, these outcomes support this novel and scalable imagery-competing task intervention as a safe means to reduce the reoccurrence of intrusive memories for frontline healthcare workers who have experienced work-related trauma. It may seem surprising that such a brief approach involving computer gameplay and no in-depth discussion of trauma with a therapist, can be helpful in reducing intrusions, and that symptom improvement persisted over six months. This may be explained by the fact that this simpler approach was developed from a mental health science perspective [1] via behavioural laboratory experiments [33, 39] drawing on the perceptual nature of intrusive memories [35] and memory’s inherent malleability [34]. As a single-symptom focussed intervention approach it targets the central symptom of intrusive memories and was predicted to also help change other related symptoms post-trauma, as demonstrated by the improvements on secondary outcomes found here. Further, this finding does not stand alone but adds to an emerging clinical evidence base for this innovative treatment approach for healthcare workers after work-related trauma [37, 38], refugees and war trauma [46], women after trauma [47] and other trauma-exposed populations including traumatic childbirth [41, 42], road traffic accidents [43, 44] and childhood trauma [45]. International clinical guidelines typically do not make recommendations based on differences in the type of traumatic event [96], and we hypothesise that other groups exposed to repeated and/or ongoing trauma, whether in an occupational (e.g., emergency service personnel, military personnel) or civilian (e.g., conflict situations) context, may benefit from this type of intervention approach, and future research is warranted. Indeed, its brevity, simplicity and accessibility circumvent many of the barriers to receiving psychological support typically encountered in such populations. As previously noted, adaptations of this intervention to specific contexts may be needed [49].

## Conclusions

In summary, healthcare workers were exposed to work-related trauma during the pandemic. We observed the need for brief, flexible, remotely-delivered and repeatable interventions as an urgent public health priority, including a subclinical-to-clinical sample for a preventing-to-treating approach. This study provided controlled evidence that this population benefitted from the single guided session, digitally delivered imagery-competing task intervention to reduce intrusive memories after trauma. Future research is required to evaluate the feasibility and efficacy of full digital delivery without a researcher, and to investigate the mechanisms underlying the persistence of sensory imagery-based memories that can haunt people after traumatic events.

## Supplementary Information

Additional file 1: Other pre-specified outcomes and additional assessments. Secondary and other pre-specified outcome description: results. Table S1. Additional baseline characteristics. Table S2. Secondary and other pre-specified outcomes across all time points. Table S3. Number of Adverse Events. Table S4. Types of Adverse Events. Table S5. Subjective Units of Distress. Table S6. Diagnostics for the multiple imputation. Table S7a. Number of intrusive memories for complete and Table 7 b for incomplete diary data. Figure S1. Time course of the number of intrusive memories day-by-day. Table S8. Breakdown of the category’other’ in the type of trauma. Table S9. Number of prior psychological traumas per category. Table S10. Coping categories based on free text responses assessed at baseline. Table S11-S15. Sensitivity analyses. Table S16. Primary endpoint by gender. Table S17. Primary endpoint by age. Table S18. Acceptance and feasibility measure. Table S19. Assessments related to procedure. Table S20. Time since traumatic events leading to intrusive memories. Procedure-related changes during the study.

## Data Availability

Data (pseudonymized) that support the findings of the primary and secondary outcomes of this study are available via Open Science Framework (OSF) at https://osf.io/mb54w/. Protocols for study procedures can be made available upon reasonable request and are subject to a Material Transfer Agreement with the corresponding author and require training in their use (Anemone™). Statistical analyses were performed at UCR (Uppsala Clinical Research Center) using SAS (SAS V.9.4 (SAS Institute, Cary, North Carolina)) and R (4.0.2 or later). Code in SAS for primary and R for secondary outcomes and other outcomes is available on OSF, https://osf.io/mb54w.

## Abbreviations

AEs: Adverse Events
CAPS-5: Clinician-Administered PTSD Scale for DSM–5
CI: Confidence interval
DSM-5: Diagnostic and Statistical Manual of mental disorders, 5th edition
IES-R: Impact of Event Scale–Revised
IRR: Incidence rate ratio
ITT: Intention-to-treat
LEC-5: Life Events Checklist for DSM-5
MAR: Missing at random
MI: Multiple imputation
MNAR: Missing not at random
PCL-5: Post-traumatic Stress Disorder Checklist 5
PP: Per protocol
PTSD: Posttraumatic stress disorder
RCT: Randomised controlled trial
SAEs: Serious Adverse Events
SCI-02: Sleep Condition Indicator (2 items)
SEQ: Stress Energy Questionnaire
SRHR: Self-Rated Health rating
SWEBO: Scale of Work Engagement and Burnout
TFCBT: Trauma-focused cognitive behavioural therapy
WHODAS: World Health Organization Disability Assessment Schedule
