# Supplementary material for: A guided single session intervention to reduce intrusive memories of work-related trauma: a randomised controlled trial with healthcare workers in the COVID-19 pandemic
Source: BMC Med. 2024 Sep 19;22:403. doi: 10.1186/s12916-024-03569-8 (PMC11414261; doi:10.1186/s12916-024-03569-8)
Supplement: Supplementary file 1 — Additional file 1: Other pre-specified outcomes and additional assessments. Secondary and other pre-specified outcome description: results. Table S1. Additional baseline characteristics. Table S2. Secondary and other pre-specified outcomes across all time points. Table S3. Number of Adverse Events. Table S4. Types of Adverse Events. Table S5. Subjective Units of Distress. Table S6. Diagnostics for the multiple imputation. Table S7a. Number of intrusive memories for complete and Table 7 b for incomplete diary data. Figure S1. Time course of the number of intrusive memories day-by-day. Table S8. Breakdown of the category’other’ in the type of trauma. Table S9. Number of prior psychological traumas per category. Table S10. Coping categories based on free text responses assessed at baseline. Table S11-S15. Sensitivity analyses. Table S16. Primary endpoint by gender. Table S17. Primary endpoint by age. Table S18. Acceptance and feasibility measure. Table S19. Assessments related to procedure. Table S20. Time since traumatic events leading to intrusive memories. Procedure-related changes during the study. [file 12916_2024_3569_MOESM1_ESM.docx]

**Supplementary Material**

# A guided single session intervention to reduce intrusive memories of work-related trauma: A randomised controlled trial with healthcare workers in the COVID-19 pandemic

Marie Kanstrup, Laura Singh, Elisabeth Johanna Leehr, Katarina E. Göransson, Sara Ahmed Pihlgren, Lalitha Iyadurai, Oili Dahl, Ann-Charlotte Falk, Veronica Lindström, Nermin Hadziosmanovic, Katja Gabrysch, Michelle L. Moulds and Emily A. Holmes

Content

[Other pre-specified outcomes and additional assessments 3](#_Toc174352797)

[Secondary and other pre-specified outcome description: results 5](#_Toc174352798)

[Table S1. Additional baseline characteristics 7](#_Toc174352799)

[Table S2. Secondary and other pre-specified outcomes across all time points. 10](#_Toc174352800)

[Table S3. Number of Adverse Events by Treatment arm 35](#_Toc174352801)

[Table S4. Types of Adverse Events by Treatment arm at each time point. 36](#_Toc174352802)

[Table S5. Subjective Units of Distress (SUDS) 38](#_Toc174352803)

[Table S6. Diagnostics for the multiple imputation 39](#_Toc174352804)

[Table S7a. Number of intrusive memories for complete diary data. 41](#_Toc174352805)

[Table S7b. Number of intrusive memories including incomplete diary data. 41](#_Toc174352806)

[Figure S1. Time course of the number of intrusive memories day-by-day. 42](#_Toc174352807)

[Table S8. Breakdown of the category ’other’ in the Type of trauma(s) leading to intrusive memories questionnaire in Table 1 of the main paper. 43](#_Toc174352808)

[Table S9. Number of prior psychological traumas (LEC-5) per category (baseline) 44](#_Toc174352809)

[Table S10. Coping categories based on free text responses assessed at baseline. 45](#_Toc174352810)

[Table S11. Sensitivity analysis Primary endpoint analysed with non-parametric Wilcoxon’s rank sum test. 47](#_Toc174352811)

[Table S12. Primary outcome missing at random (MAR). 48](#_Toc174352812)

[Table S13. Sensitivity analysis primary outcome missing not at random (MNAR). 49](#_Toc174352813)

[Table S14. Sensitivity analysis primary outcome without outliers. 52](#_Toc174352814)

[Table S15. Sensitivity analysis primary outcome excluding diary missing data (complete diary data only). 53](#_Toc174352815)

[Table S16. Primary endpoint by gender. 54](#_Toc174352816)

[Table S17. Primary endpoint by age. 55](#_Toc174352817)

[Table S18. Acceptance and feasibility measure (1 month): feedback about study participation and subsequent use of task by participant on their own. 56](#_Toc174352818)

[Table S19. Assessments related to procedure. 57](#_Toc174352819)

[Table S20. Time since traumatic events leading to intrusive memories. 59](#_Toc174352820)

[Procedure-related changes during the study 60](#_Toc174352821)

Other pre-specified outcomes and additional assessments

**Functionning**

**Sick leave** [72] was assessed with two bespoke items measuring the total number of days and the number of full work days on sick leave owing to seeking health care.

I**mpact of intrusive memories on functioning** was assessed using a rating scale derived from Iyadurai et al. 2019 [25], and using two free text response items which asked participants to provide an example of how having a specific intrusive memory had a negative impact on their functioning and, if a specific intrusive memory had reduced in frequency, how this had a positive impact on functioning.

**Difficulties in letting go of work-related thoughts** during leisure time was rated using one item [73] and a bespoke question assessed whether the above mentioned difficulties were due to intrusive memories.

**Social support** after the traumatic event was assessed using a single bespoke item asking participants to indicate how much social support they received after the traumatic event(s). Three free response items assessed **work situation** [73] and 5 items assessed **moral stress** at work.

**Coping** was assessed (baseline only) with two bespoke free response items measuring participant perceived coping during the COVID-19 pandemic (i.e., “Based on your experiences and from your perspective, are there any specific situations or factors during the COVID-19 pandemic, that you believe made it particularly difficult for you to cope?” and “Based on your experiences and from your perspective, are there any specific factors which you believe have made it easier for you to cope with the COVID-19 pandemic and its consequences?”). Across both arms, categorized answers revealed a range of situations or factors during the COVID-19 pandemic which made it either difficult to cope (e.g. lack of knowledge about COVID-19, additional work load and witnessing death and mental and/or physical suffering; or easier to cope e.g. Support from loved ones and colleagues; see **Supplementary Tables S2 and S10**).

**Other cognitive assessments**

**Appraisals of intrusions** were assessed using six items from the Psychological Problems and Negative Self-evaluations subscales of the Appraisals of Intrusive Memories scale [75,76].

The **Time Perspective Questionnaire** (TPQ; 8 items) [74] was used to assess past, present and future perspective.

A short version of the **Future Self Questionnaire** (FSQ; 3 items)assessed participants’ images of their future self-identity [77,78].

**Assessments related to procedures**

**Hotspots list.** A list of intrusive memories was collected in the intervention arm only to establish the content and number of different types of intrusive memories as part of the intervention procedure (**hotspots**. **Supplementary Table S19**).

**Number of days/nights** at work during the week of intrusion monitoring was assessed using two separate items which asked how many days/nights participants had worked during the previous week (**Supplementary Table S19**).

The number of **booster sessions** delivered by the researcher was recorded in the data collection platform, along with the date of each booster session (**Supplementary Table S19**).

**Feedback to assess acceptability and feasibility**

In addition to the feedback questionnaire (8 bespoke items) about participation used to assess participant acceptability of the task (e.g., whether they would recommend it to a colleague or friend who had a similar experience, and whether they had done the task on their own), open-ended feedback questions were used to assess feasibility of the task, the instructions, and experience of doing the task. (**Supplementary Table S18**).

As part of the procedure, **Adherence** to the intervention/control task instructions was assessed using three multiple choice items which asked participants to indicate the most important thing they did just before completing the task, what they mainly focused on during the task, and the approximate amount of time they spent completing it. An additional single item assessed the extent to which participants agreed with the statement ‘During the past 20 minutes, I followed the instructions’ on an 11-point scale (from 0 “not at all” to 10 “extremely”). Responses showed that the majority in the control arm prepared their headphones (92.6%) and 95.7% in the intervention arm indicated that they briefly saw the image of the trauma in their mind’s eye. The focus of participants in the control arm during the task was on general ideas and verbal thoughts (57.4%), daydreaming/ thinking in images (30.9%), getting as many points as possible (8.8%) and mental rotation of shapes (2.9%). In the intervention arm 94.2% focused on mental rotation of shapes, and 5.8% on getting as many points as possible. In the control arm 92.6% indicated that they spent at least 20 minutes completing the task and 7.4% indicated that they spent about ten minutes. In the intervention arm 97.1% stated that they spent at least 20 minutes with the task and 2.9% stated that they spend about ten minutes.

In both arms the extent to which participants stated, that they had followed the instructions was high (Mean ± s.d.: control: 8.17 ± 2.33, n=68; intervention: 8.55 ± 1.81, n=69).

As part of the procedure, within the intervention group, participants rated **vividness** of the trauma memory before playing Tetris (1=’no image at all’ to 5=’ exactly as clear and vivid as normal seeing’), and indicated whether **“ghost piece”** was turned off during game play. Mean vividness was mean ± s.d.: intervention: 3.78 ± 1.04, n=69. Of 69 participants with data in the intervention arm, 97.1% indicated, that ghost piece was turned off during game play.

Secondary and other pre-specified outcome description: results

**1. Characteristics of intrusive memories**

Note. the self-estimated intrusion frequency question of the **Intrusion questionnaire** **IQ** [59] (to explore convergence with diary) is in **Supplementary Table S2** and reported under secondary outcomes of the main paper.

At week 1 and 1 month, participants in the intervention arm reported a lower sense of disconnectedness from their surroundings when they experienced intrusive memories [week 1: OR=2.06 (95% CI= 1.06-4.03), P=0.0332, 1 month: OR=2.82 (95% CI= 1.03-7.94), P=0.0451] measured by item in the **Intrusion questionnaire** **IQ** [59]. Levels of intrusion-related distress, nowness, and reliving items on the IQ did not differ between the two arms at any timepoint (**Supplementary Table S2**). At week 5 and 6 month follow up, participants in the intervention arm reported that fewer triggers were associated with intrusive memories [week 5: OR=0.37 (95% CI= 0.15-0.9), P=.0299, 6-month: OR=0.33 (95% CI= 0.11-0.96), P=0.0440] (**Supplementary Table S2**).

There was no between-arm difference at week 1 or week 5 in participant ratings of vividness or intrusion-related distress as recorded in the daily diary (**Supplementary Table S2**).

**2.** **Functioning**

Participants in the intervention arm reported finding it easier to let go of work-related thoughts. Participants rated their feelings of stress and difficulties in letting go of work-related thoughts during leisure time as less likely due to intrusive memories in the intervention compared to control arm at each timepoint up to 3 months [week 1: OR=0.39 (95% CI= 0.18-0.84), P=0.0178; 1 month: OR=0.28 (95% CI= 0.10-0.70), P=0.0093; 3 months: OR=0.25 (95% CI= 0.08-0.67), P=0.0088] (**Supplementary Table S2**).

**3. Social support**

There was no between-arm difference in social support after experiencing traumatic events at any timepoint (**Supplementary Table S2**)**.**

**4 Other cognitive assessments**

Assessment of future self-identity (only assessed at 3-month follow-up) did not differ between arms, with one exception; i.e., more participants in the intervention arm reported seeing the image related to their future self-identity before the traumatic event through their own eyes, whereas more participants in the control arm reported seeing it from an outside perspective [OR=0.33 (95% CI= 0.13-0.80), P=0.0162].

Individuals in the intervention arm reported less of a sense of past time perspective than individuals in the control arm at both the 3-month [OR=0.36 (95% CI=0.17-0.72), P=0.0047] and 6-month follow-ups [OR=0.50 (95% CI= 0.25-0.97), P=0.0410]. At 6 months, participants who received the intervention reported a greater sense of future time perspective [OR=2.00 (95% CI= 1.03-3.94), P=0.0422]. Assessment of future self-identity (only assessed at the 3-month follow-up) did not differ between arms.

Regarding appraisals of intrusions (assessed with the Psychological Problems and Negative Self-evaluations subscales of the Appraisals of Intrusive Memories scale [75,76]), participants in the intervention arm had lower ratings of the appraisal that experiencing intrusions indicated psychological problems than participants in the control arm [1 month: OR=0.37 (95% CI=0.18-0.74), P=0.0059; 3 month: OR=0.35 (95% CI=0.16-0.74), P=0.0068; 6 month: OR=0.23 (95% CI=0.11-0.49), P=0.0002]. No differences were found regarding negative self-evaluation.

**Table S1.** Additional baseline characteristics including demographics, work situation, measures related to post traumatic stress, clinical background, characteristics of intrusive memories and perceived functioning and social support.

|  | **Statistics** | **Control**  **n=71** | **Intervention**  **n=73** | **Total**  **n=144** |
| --- | --- | --- | --- | --- |
| **Demographics** | | | | |
| Marital status |  |  |  |  |
| Single | n(%) | 13 (18.3) | 17 (23.3) | 30 (20.8) |
| Married or cohabiting | n(%) | 49 (69.0) | 41 (56.2) | 90 (62.5) |
| Divorced or separated | n(%) | 2 (2.8) | 4 (5.5) | 6 (4.2) |
| Living apart together (LAT) | n(%) | 7 (9.9) | 9 (12.3) | 16 (11.1) |
| Other | n(%) |  | 2 (2.7) | 2 (1.4) |
| Highest level of education |  |  |  |  |
| Elementary school | n(%) | 1 (1.4) | 1 (1.4) | 2 (1.4) |
| Upper secondary school | n(%) | 13 (18.3) | 12 (16.4) | 25 (17.4) |
| University | n(%) | 56 (78.9) | 56 (76.7) | 112 (77.8) |
| Other | n(%) | 1 (1.4) | 4 (5.5) | 5 (3.5) |
| **Work and Employment** | | | | |
| Whether the work situation changed related to COVID-19 pandemic |  |  |  |  |
| Yes | n(%) | 52 (74.3) | 60 (82.2) | 112 (78.3) |
| No | n(%) | 18 (25.7) | 13 (17.8) | 31 (21.7) |
| And if so how |  |  |  |  |
| Changed job | n(%) | 9 (17.6) | 4 (7.0) | 13 (12.0) |
| Left profession/stopped working | n(%) | 2 (3.9) | 5 (8.8) | 7 (6.5) |
| Other | n(%) | 40 (78.4) | 48 (84.2) | 88 (81.5) |
| Yearly income (SEK) |  |  |  |  |
| 0 –249 999 | n(%) | 2 (2.8) | 7 (9.6) | 9 (6.3) |
| 250 000-349 999 | n(%) | 16 (22.5) | 13 (17.8) | 29 (20.1) |
| 350 000-449 999 | n(%) | 23 (32.4) | 18 (24.7) | 41 (28.5) |
| 450 000-549 999 | n(%) | 16 (22.5) | 20 (27.4) | 36 (25.0) |
| 550 000 and above | n(%) | 14 (19.7) | 15 (20.5) | 29 (20.1) |
| **Measures related to post traumatic stress** | | | | |
| Posttraumatic Stress Disorder Checklist (PCL-5) short version (8-item scale) |  |  |  |  |
| Intrusion (0-8) | n | 71 | 73 | 144 |
|  | Mean (s.d.) | 3.63 (1.97) | 3.47 (1.69) | 3.55 (1.83) |
|  | Median (Q1-Q3) | 3.0 (2.0-5.0) | 4.0 (2.0-5.0) | 4.0 (2.0-5.0) |
|  | Min-Max | 0.0-8.0 | 0.0-7.0 | 0.0-8.0 |
| Avoidance (0-8) | n | 71 | 73 | 144 |
|  | Mean (s.d.) | 3.00 (2.31) | 2.66 (2.04) | 2.83 (2.18) |
|  | Median (Q1-Q3) | 3.0 (1.0-5.0) | 3.0 (1.0-4.0) | 3.0 (1.0-4.0) |
|  | Min-Max | 0.0-8.0 | 0.0-6.0 | 0.0-8.0 |
| Cognition/Mood (0-8) | n | 71 | 73 | 144 |
|  | Mean (s.d.) | 3.00 (2.33) | 2.55 (1.91) | 2.77 (2.13) |
|  | Median (Q1-Q3) | 3.0 (1.0-5.0) | 3.0 (1.0-4.0) | 3.0 (1.0-4.0) |
|  | Min-Max | 0.0-8.0 | 0.0-6.0 | 0.0-8.0 |
| Arousal/Reactivity (0-8) | n | 71 | 73 | 144 |
|  | Mean (s.d.) | 3.06 (2.20) | 2.86 (2.00) | 2.96 (2.10) |
|  | Median (Q1-Q3) | 3.0 (1.0-5.0) | 2.0 (2.0-4.0) | 3.0 (1.0-5.0) |
|  | Min-Max | 0.0-8.0 | 0.0-7.0 | 0.0-8.0 |
| Total (0-32) | n | 71 | 73 | 144 |
|  | Mean (s.d.) | 12.69 (7.14) | 11.53 (6.08) | 12.10 (6.63) |
|  | Median (Q1-Q3) | 12.0 (7.0-18.0) | 11.0 (7.0-16.0) | 12.0 (7.0-16.5) |
|  | Min-Max | 2.0-29.0 | 0.0-26.0 | 0.0-29.0 |
| Impact of Event Scale - Revised (IES-R) (0-32) |  |  |  |  |
| Intrusion subscale | n | 71 | 73 | 144 |
|  | Mean (s.d.) | 15.66 (6.54) | 14.93 (5.60) | 15.29 (6.07) |
|  | Median (Q1-Q3) | 16.0 (10.0-21.0) | 15.0 (11.0-19.0) | 16.0 (11.0-20.0) |
|  | Min-Max | 2.0-28.0 | 3.0-29.0 | 2.0-29.0 |
| Avoidance subscale | n | 71 | 73 | 144 |
|  | Mean (s.d.) | 13.93 (7.00) | 13.51 (6.75) | 13.72 (6.85) |
|  | Median (Q1-Q3) | 14.0 (9.0-19.0) | 14.0 (8.0-19.0) | 14.0 (8.5-19.0) |
|  | Min-Max | 0.0-27.0 | 0.0-27.0 | 0.0-27.0 |
| Self-estimated intrusion frequency to explore convergence with diary |  |  |  |  |
| Intrusion questionnaire: Frequency item (7-point scale) |  |  |  |  |
| Never | n(%) | 3 (4.2) | 1 (1.4) | 4 (2.8) |
| Once | n(%) | 7 (9.9) | 3 (4.1) | 10 (6.9) |
|  | **Statistics** | **Control**  **n=71** | **Intervention**  **n=73** | **Total**  **n=144** |
| Twice | n(%) | 10 (14.1) | 9 (12.3) | 19 (13.2) |
| Every other day | n(%) | 15 (21.1) | 16 (21.9) | 31 (21.5) |
| Once a day | n(%) | 11 (15.5) | 17 (23.3) | 28 (19.4) |
| Several times a day | n(%) | 21 (29.6) | 25 (34.2) | 46 (31.9) |
| Many times a day | n(%) | 4 (5.6) | 2 (2.7) | 6 (4.2) |
| Sum score (number of intrusions) | n | 70 | 73 | 143 |
| Sum score (number of intrusions) | Mean (s.d.) | 10.64 (12.67) | 13.42 (19.44) | 12.06 (16.48) |
|  | Median (Q1-Q3) | 5.3 (2.0-14.0) | 7.0 (3.5-21.0) | 7.0 (3.5-14.0) |
|  | Min-Max | 0.0-70.0 | 0.0-140.0 | 0.0-140.0 |
|  | Min-Max | 0.0-70.0 | 0.0-140.0 | 0.0-140.0 |
| **Clinical Background** | | | | |
| History of physical- and mental illness |  |  |  |  |
| Current physical illness |  |  |  |  |
| Yes | n(%) | 6 (8.5) | 8 (11.0) | 14 (9.7) |
| No | n(%) | 65 (91.5) | 65 (89.0) | 130 (90.3) |
| Treatment or medication for physical illness^a^ |  |  |  |  |
| Yes | n(%) | 5 (83.3) | 7 (87.5) | 12 (85.7) |
| No | n(%) | 1 (16.7) | 1 (12.5) | 2 (14.3) |
| Current/past mental illness (yes/no) |  |  |  |  |
| Yes | n(%) | 45 (63.4) | 35 (47.9) | 80 (55.6) |
| No | n(%) | 26 (36.6) | 38 (52.1) | 64 (44.4) |
| Current treatment or medication for mental illness^3^ |  |  |  |  |
| Yes | n(%) | 17 (43.6) | 19 (61.3) | 36 (51.4) |
| No | n(%) | 22 (56.4) | 12 (38.7) | 34 (48.6) |
| Family history of mental illness |  |  |  |  |
| Yes | n(%) | 23 (32.4) | 27 (37.0) | 50 (34.7) |
| No | n(%) | 48 (67.6) | 46 (63.0) | 94 (65.3) |
| **Characteristics of intrusive memories** | | | | |
| Intrusion questionnaire (IQ) (0-100) |  |  |  |  |
| Distress | n | 67 | 72 | 139 |
|  | Mean (s.d.) | 44.69 (20.04) | 46.15 (19.97) | 45.45 (19.95) |
|  | Median (Q1-Q3) | 44.0 (30.0-59.0) | 50.0 (35.0-59.5) | 48.0 (35.0-59.0) |
|  | Min-Max | 2.0-88.0 | 0.0-91.0 | 0.0-91.0 |
| Nowness | n | 67 | 72 | 139 |
|  | Mean (s.d.) | 36.90 (27.71) | 35.39 (28.08) | 36.12 (27.81) |
|  | Median (Q1-Q3) | 30.0 (15.0-54.0) | 29.0 (10.5-60.0) | 30.0 (12.0-54.0) |
|  | Min-Max | 0.0-100.0 | 0.0-100.0 | 0.0-100.0 |
| Reliving | n | 67 | 72 | 139 |
|  | Mean (s.d.) | 48.07 (24.90) | 50.63 (24.48) | 49.40 (24.63) |
|  | Median (Q1-Q3) | 49.0 (30.0-70.0) | 50.0 (31.5-70.5) | 50.0 (31.0-70.0) |
|  | Min-Max | 1.0-100.0 | 1.0-100.0 | 1.0-100.0 |
| Disconnectedness | n | 67 | 72 | 139 |
|  | Mean (s.d.) | 54.28 (31.57) | 54.76 (28.82) | 54.53 (30.07) |
|  | Median (Q1-Q3) | 57.0 (29.0-80.0) | 55.0 (37.0-78.5) | 55.0 (33.0-80.0) |
|  | Min-Max | 0.0-100.0 | 0.0-100.0 | 0.0-100.0 |
| Triggers | n | 67 | 72 | 139 |
|  | Mean (s.d.) | 59.15 (28.28) | 57.93 (25.72) | 58.52 (26.89) |
|  | Median (Q1-Q3) | 66.0 (36.0-80.0) | 57.0 (41.5-79.0) | 61.0 (39.0-80.0) |
|  | Min-Max | 0.0-100.0 | 5.0-100.0 | 0.0-100.0 |
| Diary ratings Week 0 (0-10) |  |  |  |  |
| Distress | n | 69 | 68 | 137 |
|  | Mean (s.d.) | 3.97 (2.07) | 4.25 (2.37) | 4.11 (2.22) |
|  | Median (Q1-Q3) | 4.0 (2.0-6.0) | 4.0 (3.0-5.5) | 4.0 (3.0-6.0) |
|  | Min-Max | 0.0-8.0 | 0.0-10.0 | 0.0-10.0 |
| Vividness | n | 68 | 68 | 136 |
|  | Mean (s.d.) | 4.56 (2.54) | 4.26 (2.37) | 4.41 (2.45) |
|  | Median (Q1-Q3) | 4.0 (3.0-6.0) | 4.0 (3.0-6.0) | 4.0 (3.0-6.0) |
|  | Min-Max | 0.0-10.0 | 0.0-10.0 | 0.0-10.0 |
| **Other pre-specified outcome measures at baseline** | | | | |
| **Perceived functioning and social support** | | | | |
| Social support after traumatic event (1-10)^b^ | n | 71 | 73 | 144 |
|  | Mean (s.d.) | 6.39 (2.73) | 6.01 (3.02) | 6.20 (2.87) |
|  | Median (Q1-Q3) | 7.0 (4.0-9.0) | 6.0 (3.0-8.0) | 6.0 (4.0-8.5) |
|  | Min-Max | 1.0-10.0 | 1.0-10.0 | 1.0-10.0 |
|  | **Statistics** | **Control**  **n=71** | **Intervention**  **n=73** | **Total**  **n=144** |
| Difficulties in letting go of work-related thoughts during leisure time (0-4) | n | 71 | 71 | 142 |
|  | Mean (s.d.) | 1.94 (1.19) | 1.90 (1.20) | 1.92 (1.19) |
|  | Median (Q1-Q3) | 2.0 (1.0-3.0) | 2.0 (1.0-3.0) | 2.0 (1.0-3.0) |
|  | Min-Max | 0.0-4.0 | 0.0-4.0 | 0.0-4.0 |
| Whether above mentioned difficulties are due to intrusive memories |  |  |  |  |
| Yes | n(%) | 40 (56.3) | 35 (49.3) | 75 (52.8) |
| No | n(%) | 31 (43.7) | 36 (50.7) | 67 (47.2) |
| Moral stress at work (5-20) | n | 70 | 73 | 143 |
|  | Mean (s.d.) | 13.47 (3.99) | 13.00 (3.65) | 13.23 (3.81) |
|  | Median (Q1-Q3) | 14.0 (11.0-16.0) | 13.0 (10.0-16.0) | 13.0 (11.0-16.0) |
|  | Min-Max | 5.0-20.0 | 5.0-20.0 | 5.0-20.0 |
| Coping^c^ |  |  |  |  |
| Other cognitive assessments |  |  |  |  |
| Appraisals of IMs (0-300) |  |  |  |  |
| Psychological problems | n | 71 | 73 | 144 |
|  | Mean (s.d.) | 50.56 (62.93) | 46.03 (53.69) | 48.26 (58.27) |
|  | Median (Q1-Q3) | 20.0 (0.0-90.0) | 20.0 (0.0-80.0) | 20.0 (0.0-80.0) |
|  | Min-Max | 0.0-230.0 | 0.0-240.0 | 0.0-240.0 |
| Negative self-evaluation | n | 71 | 73 | 144 |
|  | Mean (s.d.) | 47.32 (74.87) | 37.81 (54.24) | 42.50 (65.18) |
|  | Median (Q1-Q3) | 0.0 (0.0-80.0) | 20.0 (0.0-60.0) | 10.0 (0.0-60.0) |
|  | Min-Max | 0.0-270.0 | 0.0-270.0 | 0.0-270.0 |
| Time perspective questionnaire (TPQ) |  |  |  |  |
| Past (3-15) | n | 71 | 72 | 143 |
|  | Mean (s.d.) | 9.42 (3.71) | 9.38 (3.03) | 9.40 (3.37) |
|  | Median (Q1-Q3) | 9.0 (6.0-13.0) | 9.5 (7.0-11.5) | 9.0 (7.0-12.0) |
|  | Min-Max | 3.0-15.0 | 3.0-15.0 | 3.0-15.0 |
| Present (3-10) | n | 71 | 72 | 143 |
|  | Mean (s.d.) | 6.15 (2.46) | 6.50 (2.53) | 6.33 (2.50) |
|  | Median (Q1-Q3) | 6.0 (4.0-8.0) | 6.0 (4.5-9.0) | 6.0 (4.0-8.0) |
|  | Min-Max | 2.0-10.0 | 2.0-10.0 | 2.0-10.0 |
| Future (3-15) | n | 71 | 72 | 143 |
|  | Mean (s.d.) | 9.86 (2.79) | 9.64 (2.53) | 9.75 (2.65) |
|  | Median (Q1-Q3) | 10.0 (8.0-12.0) | 10.0 (8.0-11.0) | 10.0 (8.0-12.0) |
|  | Min-Max | 3.0-15.0 | 3.0-15.0 | 3.0-15.0 |

*Note*. ^a^ Only asked if previous item had been answered yes. ^b^ “Self-rated “social support after traumatic event” was coded on an erroneous scale (1-10 instead of 0-10); ^c^ Categories will be presented in **Supplementary Table S10**.

**Table S2.** Secondary and other pre-specified outcomes across all time points.

|  | **Statistics** | **Control** | **Inter-vention** | **Control** | **Inter-vention** | **Treat-ment effect estimate (95% CI)** | **Sig.** | **Control** | **Inter-vention** | **Treat-ment effect estimate (95% CI)** | **Sig.** | **Control** | **Inter-vention** | **Treat-ment effect estimate (95% CI)** | **Sig.** | **Control** | **Inter-vention** | **Treat-ment effect estimate (95% CI)** | **Sig.** |
| --- | --- | --- | --- | --- | --- | --- | --- | --- | --- | --- | --- | --- | --- | --- | --- | --- | --- | --- | --- |
|  |  | *Baseline* | | *Week 1* | | | | *1 Month/ Week 5* | | | | *3 Month* | | | | *6 Month* | | | |
| **Secondary Outcomes** | | | | | | | | | | | | | | | | | | | |
| **Clinical symptoms** | | | | | | | | | | | | | | | | | | | |
| **PTSD symptoms and post-traumatic distress** | | | | | | | | | | | | | | | | | | | |
| Posttraumatic Stress Disorder Checklist (PCL-5) short version (8-item scale) | | | | | | | | | | | | | | | | | | | |
| Intrusion (0-8) | n | 71 | 73 | 61 | 54 |  |  | 55 | 51 |  |  | 55 | 47 |  |  | 58 | 52 |  |  |
|  | Mean (s.d.) | 3.63 (1.97) | 3.47 (1.69) | 2.66 (1.81) | 1.85 (1.61) | 0.45 (0.23-0.86)^a^ | **0.0168** | 2.65 (1.91) | 1.39 (1.47) | 0.28 (0.14-0.56)^a^ | **0.0004** | 2.29 (1.87) | 1.09 (1.65) | 0.25 (0.12-0.52)^a^ | **0.0003** | 2.02 (1.83) | 1.02 (1.57) | 0.30 (0.15-0.61)^a^ | **0.0010** |
|  | Median (Q1-Q3) | 3.0 (2.0-5.0) | 4.0 (2.0-5.0) | 2.0 (1.0-4.0) | 2.0 (1.0-3.0) | 0.47 (0.09-0.84)^e^ |  | 2.0 (1.0-4.0) | 1.0 (0.0-2.0) | 0.74 (0.34-1.14)^e^ |  | 2.0 (1.0-4.0) | 0.0 (0.0-2.0) | 0.68 (0.27-1.08)^e^ |  | 2.0 (0.0-3.0) | 0.0 (0.0-2.0) | 0.58 (0.20-0.97)^e^ |  |
|  | Min-Max | 0.0-8.0 | 0.0-7.0 | 0.0-7.0 | 0.0-7.0 |  |  | 0.0-7.0 | 0.0-5.0 |  |  | 0.0-7.0 | 0.0-8.0 |  |  | 0.0-7.0 | 0.0-8.0 |  |  |
| Avoidance (0-4) | n | 71 | 73 | 61 | 54 |  |  | 55 | 51 |  |  | 55 | 47 |  |  | 58 | 52 |  |  |
|  | Mean (s.d.) | 3.00 (2.31) | 2.66 (2.04) | 2.46 (2.05) | 2.26 (2.10) | 0.80 (0.42-1.53)^a^ | 0.5046 | 2.29 (2.29) | 1.22 (1.69) | 0.41 (0.20-0.83)^a^ | **0.0143** | 2.09 (2.18) | 0.83 (1.70) | 0.26 (0.12-0.55)^a^ | **0.0006** | 1.83 (1.80) | 0.79 (1.36) | 0.28 (0.13-0.58)^a^ | **0.0007** |
|  | Median (Q1-Q3) | 3.0 (1.0-5.0) | 3.0 (1.0-4.0) | 2.0 (1.0-4.0) | 2.0 (0.0-4.0) | 0.10  (-0.27-0.47)^e^ |  | 2.0 (0.0-4.0) | 0.0 (0.0-2.0) | 0.53 (0.14-0.92)^e^ |  | 1.0 (0.0-4.0) | 0.0 (0.0-1.0) | 0.64 (0.24-1.04)^e^ |  | 2.0 (0.0-3.0) | 0.0 (0.0-1.0) | 0.65 (0.26-1.03)^e^ |  |
|  | Min-Max | 0.0-8.0 | 0.0-6.0 | 0.0-7.0 | 0.0-8.0 |  |  | 0.0-8.0 | 0.0-7.0 |  |  | 0.0-7.0 | 0.0-7.0 |  |  | 0.0-6.0 | 0.0-5.0 |  |  |

|  | **Statistics** | **Control** | **Inter-vention** | **Control** | **Inter-vention** | **Treat-ment effect estimate (95% CI)** | **Sig.** | **Control** | **Inter-vention** | **Treat-ment effect estimate (95% CI)** | **Sig.** | **Control** | **Inter-vention** | **Treat-ment effect estimate (95% CI)** | **Sig.** | **Control** | **Inter-vention** | **Treat-ment effect estimate (95% CI)** | **Sig.** |
| --- | --- | --- | --- | --- | --- | --- | --- | --- | --- | --- | --- | --- | --- | --- | --- | --- | --- | --- | --- |
|  |  | *Baseline* | | *Week 1* | | | | *1 Month/ Week 5* | | | | *3 Month* | | | | *6 Month* | | | |
| Cognition/  Mood (0-8) | n | 71 | 73 | 61 | 54 |  |  | 55 | 51 |  |  | 55 | 47 |  |  | 58 | 52 |  |  |
|  | Mean (s.d.) | 3.00 (2.33) | 2.55 (1.91) | 2.54 (2.31) | 1.35 (1.79) | 0.37 (0.19-0.72)^a^ | **0.0036** | 2.49 (2.24) | 1.04 (1.66) | 0.22 (0.10-0.46)^a^ | **<0.0001** | 2.18 (2.20) | 1.00 (1.77) | 0.27 (0.12-0.56)^a^ | **0.0006** | 2.33 (2.22) | 0.87 (1.60) | 0.22 (0.10-0.46)^a^ | **<0.0001** |
|  | Median (Q1-Q3) | 3.0 (1.0-5.0) | 3.0 (1.0-4.0) | 2.0 (1.0-4.0) | 1.0 (0.0-2.0) | 0.57 (0.19-0.95)^e^ |  | 2.0 (1.0-4.0) | 0.0 (0.0-1.0) | 0.73 (0.33-1.13)^e^ |  | 1.0 (0.0-4.0) | 0.0 (0.0-1.0) | 0.59 (0.19-0.99)^e^ |  | 2.0 (0.0-4.0) | 0.0 (0.0-1.0) | 0.75 (0.36-1.14)^e^ |  |
|  | Min-Max | 0.0-8.0 | 0.0-6.0 | 0.0-8.0 | 0.0-8.0 |  |  | 0.0-8.0 | 0.0-8.0 |  |  | 0.0-7.0 | 0.0-8.0 |  |  | 0.0-8.0 | 0.0-8.0 |  |  |
| Arousal/  Reactivity (0-8) | n | 71 | 73 | 61 | 54 |  |  | 55 | 51 |  |  | 55 | 47 |  |  | 58 | 52 |  |  |
|  | Mean (s.d.) | 3.06 (2.20) | 2.86 (2.00) | 2.92 (2.04) | 1.44 (1.66) | 0.24 (0.12-0.48)^a^ | **<0.0001** | 2.95 (2.16) | 1.25 (1.48) | 0.19 (0.09-0.39)^a^ | **<0.0001** | 2.42 (2.02) | 0.89 (1.32) | 0.19 (0.09-0.40)^a^ | **<0.0001** | 2.19 (1.83) | 0.79 (1.24) | 0.18 (0.08-0.37)^a^ | **<0.0001** |
|  | Median (Q1-Q3) | 3.0 (1.0-5.0) | 2.0 (2.0-4.0) | 3.0 (1.0-5.0) | 1.0 (0.0-2.0) | 0.79 (0.40-1.17)^e^ |  | 2.0 (1.0-4.0) | 1.0 (0.0-2.0) | 0.91 (0.50-1.31)^e^ |  | 2.0 (1.0-3.0) | 0.0 (0.0-1.0) | 0.88 (0.46-1.29)^e^ |  | 2.0 (1.0-4.0) | 0.0 (0.0-1.0) | 0.89 (0.49-1.28)^e^ |  |
|  | Min-Max | 0.0-8.0 | 0.0-7.0 | 0.0-6.0 | 0.0-6.0 |  |  | 0.0-8.0 | 0.0-5.0 |  |  | 0.0-7.0 | 0.0-5.0 |  |  | 0.0-7.0 | 0.0-5.0 |  |  |
| Total (0-32) | n | 71 | 73 | 61 | 54 |  |  | 55 | 51 |  |  | 55 | 47 |  |  | 58 | 52 |  |  |
|  | Mean (s.d.) | 12.69 (7.14) | 11.53 (6.08) | 10.57 (6.91) | 6.91 (6.15) | 0.37 (0.19-0.71)^a^ | **0.0031** | 10.38 (7.30) | 4.90 (5.29) | 0.21 (0.10-0.42)^a^ | **<0.0001** | 8.98 (6.92) | 3.81 (5.17) | 0.19 (0.09-0.40)^a^ | **<0.0001** | 8.36 (6.47) | 3.46 (4.83) | 0.19 (0.09-0.39)^a^ | **<0.0001** |
|  | Median (Q1-Q3) | 12.0 (7.0-18.0) | 11.0 (7.0-16.0) | 9.0 (5.0-17.0) | 5.5 (2.0-10.0) | 0.56 (0.18-0.94)^e^ |  | 10.0 (4.0-15.0) | 3.0 (1.0-6.0) | 0.85 (0.45-1.26)^e^ |  | 7.0 (3.0-13.0) | 2.0 (0.0-5.0) | 0.84 (0.43-1.25)^e^ |  | 7.0 (3.0-14.0) | 2.0 (0.0-4.0) | 0.85 (0.46-1.25)^e^ |  |
|  | Min-Max | 2.0-29.0 | 0.0-26.0 | 0.0-26.0 | 0.0-25.0 |  |  | 1.0-31.0 | 0.0-22.0 |  |  | 0.0-26.0 | 0.0-27.0 |  |  | 0.0-27.0 | 0.0-25.0 |  |  |

|  | **Statistics** | **Control** | **Inter-vention** | **Control** | **Inter-vention** | **Treat-ment effect estimate (95% CI)** | **Sig.** | **Control** | **Inter-vention** | **Treat-ment effect estimate (95% CI)** | **Sig.** | **Control** | **Inter-vention** | **Treat-ment effect estimate (95% CI)** | **Sig.** | **Control** | **Inter-vention** | **Treat-ment effect estimate (95% CI)** | **Sig.** |
| --- | --- | --- | --- | --- | --- | --- | --- | --- | --- | --- | --- | --- | --- | --- | --- | --- | --- | --- | --- |
|  |  | *Baseline* | | *Week 1* | | | | *1 Month/ Week 5* | | | | *3 Month* | | | | *6 Month* | | | |
| Impact of Event Scale - Revised (IES-R) (0-32) | | | | | | | | | | | | | | | | | | | |
| Intrusion subscale | n | 71 | 73 | 61 | 56 |  |  | 56 | 51 |  |  | 56 | 49 |  |  | 60 | 53 |  |  |
|  | Mean (s.d.) | 15.66 (6.54) | 14.93 (5.60) | 13.44 (6.59) | 8.36 (6.01) | 0.24 (0.12-0.46)^a^ | **<0.0001** | 11.63 (7.44) | 5.25 (5.51) | 0.16 (0.08-0.34)^a^ | **<0.0001** | 9.52 (6.55) | 4.18 (5.16) | 0.17 (0.08-0.34)^a^ | **<0.0001** | 8.87 (6.74) | 4.36 (5.00) | 0.25 (0.12-0.49)^a^ | **<0.0001** |
|  | Median (Q1-Q3) | 16.0 (10.0-21.0) | 15.0 (11.0-19.0) | 14.0 (8.0-18.0) | 7.0 (4.0-11.0) | 0.80 (0.42-1.19)^e^ |  | 11.0 (6.0-15.5) | 3.0 (1.0-7.0) | 0.97 (0.56-1.37)^e^ |  | 8.0 (4.0-13.5) | 2.0 (0.0-6.0) | 0.90 (0.49-1.30)^e^ |  | 7.0 (3.0-15.0) | 3.0 (1.0-5.0) | 0.75 (0.37-1.14)^e^ |  |
|  | Min-Max | 2.0-28.0 | 3.0-29.0 | 2.0-27.0 | 0.0-29.0 |  |  | 0.0-29.0 | 0.0-25.0 |  |  | 0.0-27.0 | 0.0-24.0 |  |  | 0.0-27.0 | 0.0-25.0 |  |  |
| Avoidance subscale | n | 71 | 73 | 61 | 56 |  |  | 56 | 51 |  |  | 56 | 49 |  |  | 60 | 53 |  |  |
|  | Mean (s.d.) | 13.93 (7.00) | 13.51 (6.75) | 11.48 (6.83) | 10.43 (7.15) | 0.73 (0.39-1.38)^a^ | 0.3355 | 10.79 (7.42) | 6.06 (7.06) | 0.25 (0.12-0.51)^a^ | **0.0002** | 9.14 (6.75) | 4.96 (6.48) | 0.26 (0.12-0.52)^a^ | **0.0002** | 7.75 (6.80) | 4.77 (5.74) | 0.41 (0.21-0.79)^a^ | **0.0082** |
|  | Median (Q1-Q3) | 14.0 (9.0-19.0) | 14.0 (8.0-19.0) | 11.0 (6.0-18.0) | 8.0 (5.0-17.0) | 0.15 (-0.22-0.52)^e^ |  | 9.5 (5.0-16.0) | 3.0 (1.0-8.0) | 0.65 (0.26-1.05)^e^ |  | 8.0 (4.0-13.5) | 2.0 (0.0-8.0) | 0.63 (0.23-1.03)^e^ |  | 6.5 (2.0-11.5) | 2.0 (0.0-7.0) | 0.47 (0.09-0.85)^e^ |  |
|  | Min-Max | 0.0-27.0 | 0.0-27.0 | 0.0-26.0 | 0.0-27.0 |  |  | 0.0-30.0 | 0.0-24.0 |  |  | 0.0-27.0 | 0.0-25.0 |  |  | 0.0-25.0 | 0.0-21.0 |  |  |
| Self-estimated intrusion frequency to explore convergence with diary at Baseline, Week 1, Week 5, 3 Month and 6 Month | | | | | | | | | | | | | | | | | | | |
| Intrusion questionnaire: Frequency item (7-point scale) |  |  |  |  |  | 0.23 (0.11-0.45)^a^ | **<0.0001** |  |  | 0.20 (0.09-0.40)^a^ | **<0.0001** |  |  | 0.25 (0.11-0.52)^a^ | **0.0004** |  |  | 0.36 (0.17-0.75)^a^ | **0.0067** |
| Never | n(%) | 3 (4.2) | 1 (1.4) | 3 (4.8) | 7 (12.3) |  |  | 11 (18.6) | 28 (52.8) |  |  | 19 (33.9) | 32 (65.3) |  |  | 25 (41.7) | 36 (67.9) |  |  |
| Once | n(%) | 7 (9.9) | 3 (4.1) | 4 (6.5) | 10 (17.5) |  |  | 8 (13.6) | 9 (17.0) |  |  | 13 (23.2) | 12 (24.5) |  |  | 17 (28.3) | 9 (17.0) |  |  |

|  | **Statistics** | **Control** | **Inter-vention** | **Control** | **Inter-vention** | **Treat-ment effect estimate (95% CI)** | **Sig.** | **Control** | **Inter-vention** | **Treat-ment effect estimate (95% CI)** | **Sig.** | **Control** | **Intervention** | **Treat-ment effect estimate (95% CI)** | **Sig.** | **Control** | **Intervention** | **Treat-ment effect estimate (95% CI)** | **Sig.** |
| --- | --- | --- | --- | --- | --- | --- | --- | --- | --- | --- | --- | --- | --- | --- | --- | --- | --- | --- | --- |
|  |  | *Baseline* | | *Week 1* | | | | *1 Month/ Week 5* | | | | *3 Month* | | | | *6 Month* | | | |
| Twice | n(%) | 10 (14.1) | 9 (12.3) | 11 (17.7) | 22 (38.6) |  |  | 11 (18.6) | 8 (15.1) |  |  | 14 (25.0) | 2 (4.1) |  |  | 10 (16.7) | 4 (7.5) |  |  |
| Every other day | n(%) | 15 (21.1) | 16 (21.9) | 14 (22.6) | 7 (12.3) |  |  | 13 (22.0) | 5 (9.4) |  |  | 8 (14.3) | 0 (0) |  |  | 4 (6.7) | 3 (5.7) |  |  |
| Once a day | n(%) | 11 (15.5) | 17 (23.3) | 10 (16.1) | 7 (12.3) |  |  | 8 (13.6) | 1 (1.9) |  |  | 1 (1.8) | 0 (0) |  |  | 3 (5.0) | 0 (0) |  |  |
| Several times a day | n(%) | 21 (29.6) | 25 (34.2) | 20 (32.3) | 3 (5.3) |  |  | 8 (13.6) | 2 (3.8) |  |  | 1 (1.8) | 3 (6.1) |  |  | 0 (0) | 1 (1.9) |  |  |
| Many times a day | n(%) | 4 (5.6) | 2 (2.7) | 0 (0) | 1 (1.8) |  |  | 0 (0) | 0 (0) |  |  | 0 | 0 |  |  | 1 (1.7) | 0 (0) |  |  |
| Sum score (number of intrusions) | n | 70 | 73 | 62 | 57 |  |  | 59 | 53 |  |  | 56 | 49 |  |  | 60 | 53 |  |  |
|  | Mean (s.d.) | 10.64 (12.67) | 13.42 (19.44) | 8.55 (8.74) | 6.90 (27.71) | 0.81 (0.29-2.17)^c^ | 0.6737 | 5.91 (9.52) | 2.52 (9.72) | 0.43 (0.14-1.11)^c^ | 0.1022 | 1.73 (2.98) | 4.18 (20.41) | 2.42 (0.48-17.54)^c^ | 0.3804 | 1.78 (4.67) | 0.78 (2.08) | 0.44 (0.13-1.22)^c^ | 0.1384 |
|  | Median (Q1-Q3) | 5.3 (2.0-14.0) | 7.0 (3.5-21.0) | 3.5 (2.0-14.0) | 2.0 (1.0-3.5) | 0.08  (-0.28-0.45)^e^ |  | 2.0 (1.0-7.0) | 0.0 (0.0-2.0) | 0.35  (-0.03-0.73)^e^ |  | 1.0 (0.0-2.0) | 0.0 (0.0-1.0) | -0.17  (-0.56-0.21)^e^ |  | 1.0 (0.0-2.0) | 0.0 (0.0-1.0) | 0.27  (-0.10-0.65)^e^ |  |
|  | Min-Max | 0.0-70.0 | 0.0-140.0 | 0.0-35.0 | 0.0-210.0 |  |  | 0.0-49.0 | 0.0-70.0 |  |  | 0.0-21.0 | 0.0-140.0 |  |  | 0.0-35.0 | 0.0-14.0 |  |  |
| Intrusion questionnaire (0-100) | | | | | | | | | | | | | | | | | | | |
| Distress | n | 67 | 72 | 59 | 50 |  |  | 46 | 25 |  |  | 37 | 17 |  |  | 35 | 17 |  |  |
|  | Mean (s.d.) | 44.69 (20.04) | 46.15 (19.97) | 36.83 (22.33) | 31.24 (19.18) | 0.64 (0.33-1.24)^a^ | 0.1900 | 38.83 (24.08) | 38.72 (27.04) | 0.85 (0.36-2.00)^a^ | 0.7093 | 35.24 (23.17) | 44.06 (26.01) | 1.97 (0.73-5.49)^a^ | 0.1855 | 34.83 (22.95) | 38.06 (28.13) | 1.04 (0.38-2.88)^a^ | 0.9369 |
|  | Median (Q1-Q3) | 44.0 (30.0-59.0) | 50.0 (35.0-59.5) | 41.0 (16.0-52.0) | 27.0 (19.0-48.0) | 0.27  (-0.12-0.65)^e^ |  | 37.5 (20.0-51.0) | 31.0 (18.0-50.0) | 0.00  (-0.49-0.50)^e^ |  | 25.0 (13.0-56.0) | 44.0 (25.0-60.0) | -0.37  (-0.96-0.23)^e^ |  | 32.0 (18.0-55.0) | 31.0 (16.0-42.0) | -0.13  (-0.72-0.46)^e^ |  |
|  | Min-Max | 2.0-88.0 | 0.0-91.0 | 0.0-84.0 | 1.0-84.0 |  |  | 0.0-94.0 | 0.0-100.0 |  |  | 0.0-93.0 | 8.0-100.0 |  |  | 0.0-87.0 | 9.0-100.0 |  |  |

|  | **Statistics** | **Control** | **Inter-vention** | **Control** | **Inter-vention** | **Treatment effect estimate (95% CI)** | **Sig.** | **Control** | **Inter-vention** | **Treatment effect estimate (95% CI)** | **Sig.** | **Control** | **Intervention** | **Treatment effect estimate (95% CI)** | **Sig.** | **Control** | **Intervention** | **Treatment effect estimate (95% CI)** | **Sig.** |
| --- | --- | --- | --- | --- | --- | --- | --- | --- | --- | --- | --- | --- | --- | --- | --- | --- | --- | --- | --- |
|  |  | *Baseline* | | *Week 1* | | | | *1 Month/ Week 5* | | | | *3 Month* | | | | *6 Month* | | | |
| Nowness | n | 67 | 72 | 59 | 50 |  |  | 46 | 25 |  |  | 37 | 17 |  |  | 35 | 17 |  |  |
|  | Mean (s.d.) | 36.90 (27.71) | 35.39 (28.08) | 23.47 (23.88) | 25.70 (24.31) | 1.31 (0.68-2.52)^a^ | 0.4207 | 25.20 (25.62) | 27.76 (24.33) | 1.35 (0.59-3.15)^a^ | 0.4780 | 20.76 (22.44) | 30.94 (29.95) | 1.99 (0.73-5.59)^a^ | 0.1836 | 26.97 (29.64) | 15.29 (16.39) | 0.71 (0.26-1.90)^a^ | 0.4949 |
|  | Median (Q1-Q3) | 30.0 (15.0-54.0) | 29.0 (10.5-60.0) | 15.0 (4.0-37.0) | 19.0 (6.0-39.0) | -0.09  (-0.47-0.29)^e^ |  | 15.0 (3.0-48.0) | 21.0 (6.0-49.0) | -0.10  (-0.60-0.39)^e^ |  | 15.0 (1.0-33.0) | 17.0 (11.0-43.0) | -0.41  (-1.00-0.19)^e^ |  | 18.0 (1.0-52.0) | 15.0 (2.0-22.0) | 0.45  (-0.15-1.05)^e^ |  |
|  | Min-Max | 0.0-100.0 | 0.0-100.0 | 0.0-86.0 | 0.0-87.0 |  |  | 0.0-94.0 | 0.0-81.0 |  |  | 0.0-80.0 | 0.0-86.0 |  |  | 0.0-100.0 | 0.0-68.0 |  |  |
| Reliving | n | 67 | 72 | 59 | 50 |  |  | 46 | 25 |  |  | 37 | 17 |  |  | 35 | 17 |  |  |
|  | Mean (s.d.) | 48.07 (24.90) | 50.63 (24.48) | 41.31 (26.42) | 36.50 (23.58) | 0.73 (0.38-1.41)^a^ | 0.3551 | 43.00 (28.86) | 39.36 (27.80) | 0.74 (0.32-1.73)^a^ | 0.4917 | 31.95 (23.42) | 43.24 (25.24) | 2.22 (0.82-6.18)^a^ | 0.1204 | 37.03 (27.45) | 34.12 (27.06) | 0.85 (0.31-2.29)^a^ | 0.7401 |
|  | Median (Q1-Q3) | 49.0 (30.0-70.0) | 50.0 (31.5-70.5) | 41.0 (20.0-60.0) | 30.5 (17.0-53.0) | 0.19  (-0.19-0.57)^e^ |  | 47.5 (21.0-62.0) | 29.0 (21.0-51.0) | 0.13  (-0.37-0.62)^e^ |  | 31.0 (8.0-47.0) | 44.0 (25.0-55.0) | -0.47  (-1.07-0.12)^e^ |  | 31.0 (12.0-60.0) | 30.0 (15.0-41.0) | 0.11  (-0.49-0.70)^e^ |  |
|  | Min-Max | 1.0-100.0 | 1.0-100.0 | 0.0-100.0 | 1.0-90.0 |  |  | 0.0-100.0 | 0.0-95.0 |  |  | 0.0-79.0 | 4.0-100.0 |  |  | 0.0-86.0 | 0.0-100.0 |  |  |

|  | **Statistics** | **Control** | **Inter-vention** | **Control** | **Inter-vention** | **Treat-ment effect estimate (95% CI)** | **Sig.** | **Control** | **Inter-vention** | **Treat-ment effect estimate (95% CI)** | **Sig.** | **Control** | **Inter-vention** | **Treat-ment effect estimate (95% CI)** | **Sig.** | **Control** | **Inter-vention** | **Treat-ment effect estimate (95% CI)** | **Sig.** |
| --- | --- | --- | --- | --- | --- | --- | --- | --- | --- | --- | --- | --- | --- | --- | --- | --- | --- | --- | --- |
|  |  | *Baseline* | | *Week 1* | | | | *1 Month/ Week 5* | | | | *3 Month* | | | | *6 Month* | | | |
| Disconnectedness | n | 67 | 72 | 59 | 50 |  |  | 46 | 25 |  |  | 37 | 17 |  |  | 35 | 17 |  |  |
|  | Mean (s.d.) | 54.28 (31.57) | 54.76 (28.82) | 40.66 (27.60) | 52.06 (28.55) | 2.06 (1.06-4.03)^a^ | **0.0332** | 39.11 (25.62) | 49.08 (29.07) | 2.04 (0.86-4.92)^a^ | 0.1056 | 37.62 (28.97) | 53.53 (27.35) | 2.82 (1.03-7.94)^a^ | **0.0451** | 46.57 (29.29) | 40.47 (34.40) | 0.71 (0.25-2.01)^a^ | 0.5210 |
|  | Median (Q1-Q3) | 57.0 (29.0-80.0) | 55.0 (37.0-78.5) | 39.0 (19.0-57.0) | 51.5 (32.0-76.0) | -0.41  (-0.79-  -0.02)^e^ |  | 40.5 (21.0-54.0) | 50.0 (27.0-69.0) | -0.37  (-0.87-0.13)^e^ |  | 30.0 (18.0-53.0) | 50.0 (40.0-76.0) | -0.56  (-1.16-0.04)^e^ |  | 50.0 (22.0-72.0) | 43.0 (4.0-62.0) | 0.20  (-0.40-0.79)^e^ |  |
|  | Min-Max | 0.0-100.0 | 0.0-100.0 | 0.0-100.0 | 0.0-100.0 |  |  | 0.0-94.0 | 2.0-100.0 |  |  | 0.0-100.0 | 3.0-100.0 |  |  | 0.0-100.0 | 0.0-100.0 |  |  |
| Triggers | n | 67 | 72 | 59 | 50 |  |  | 46 | 25 |  |  | 37 | 17 |  |  | 35 | 17 |  |  |
|  | Mean (s.d.) | 59.15 (28.28) | 57.93 (25.72) | 52.00 (28.62) | 42.10 (25.76) | 0.53 (0.27-1.02)^a^ | 0.0578 | 58.13 (27.26) | 41.24 (33.85) | 0.37 (0.15-0.90)^a^ | **0.0299** | 53.14 (33.79) | 47.47 (29.05) | 0.77 (0.29-2.05)^a^ | 0.5998 | 50.91 (25.59) | 36.35 (30.86) | 0.33 (0.11-0.96)^a^ | **0.0440** |
|  | Median (Q1-Q3) | 66.0 (36.0-80.0) | 57.0 (41.5-79.0) | 54.0 (25.0-75.0) | 39.0 (20.0-58.0) | 0.36  (-0.02-0.75)^e^ |  | 59.0 (40.0-76.0) | 41.0 (11.0-74.0) | 0.57 (0.06-1.07)^e^ |  | 55.0 (23.0-79.0) | 43.0 (24.0-59.0) | 0.17  (-0.41-0.76)^e^ |  | 50.0 (27.0-74.0) | 24.0 (17.0-54.0) | 0.53  (-0.07-1.13)^e^ |  |
|  | Min-Max | 0.0-100.0 | 5.0-100.0 | 0.0-100.0 | 1.0-95.0 |  |  | 3.0-100.0 | 0.0-100.0 |  |  | 0.0-100.0 | 1.0-96.0 |  |  | 3.0-97.0 | 0.0-100.0 |  |  |

|  | **Statistics** | **Control** | **Inter-vention** | **Control** | **Inter-vention** | **Treat-ment effect estimate (95% CI)** | **Sig.** | **Control** | **Inter-vention** | **Treat-ment effect estimate (95% CI)** | **Sig.** | **Control** | **Inter-vention** | **Treat-ment effect estimate (95% CI)** | **Sig.** | **Control** | **Inter-vention** | **Treat-ment effect estimate (95% CI)** | **Sig.** |
| --- | --- | --- | --- | --- | --- | --- | --- | --- | --- | --- | --- | --- | --- | --- | --- | --- | --- | --- | --- |
|  |  | *Baseline* | | *Week 1* | | | | *1 Month/ Week 5* | | | | *3 Month* | | | | *6 Month* | | | |
| **Other outcome measures** | | | | | | | | | | | | | | | | | | | |
| **Functioning** | | | | | | | | | | | | | | | | | | | |
| Scale of Work Engagement and Burnout (SWEBO) - Burnout subscale | | | | | | | | | | | | | | | | | | | |
| Exhaustion (1-4) | n | 71 | 70 |  |  |  |  |  |  |  |  |  |  |  |  | 58 | 50 |  |  |
|  | Mean (s.d.) | 2.14 (0.78) | 2.12 (0.81) |  |  |  |  |  |  |  |  |  |  |  |  | 1.95 (0.75) | 1.77 (0.74) | 0.64 (0.32-1.25)^a^ | 0.1920 |
|  | Median (Q1-Q3) | 2.0 (1.7-3.0) | 2.0 (1.3-3.0) |  |  |  |  |  |  |  |  |  |  |  |  | 2.0 (1.3-2.3) | 1.7 (1.0-2.3) | 0.25  (-0.13-0.63)^e^ |  |
|  | Min-Max | 1.0-4.0 | 1.0-4.0 |  |  |  |  |  |  |  |  |  |  |  |  | 1.0-4.0 | 1.0-4.0 |  |  |
| Disengagement  (1-4) | n | 71 | 70 |  |  |  |  |  |  |  |  |  |  |  |  | 58 | 50 |  |  |
|  | Mean (s.d.) | 1.82 (0.73) | 1.83 (0.69) |  |  |  |  |  |  |  |  |  |  |  |  | 1.66 (0.66) | 1.42 (0.60) | 0.45 (0.22-0.90)^a^ | **0.0245** |
|  | Median (Q1-Q3) | 1.7 (1.3-2.0) | 1.7 (1.3-2.3) |  |  |  |  |  |  |  |  |  |  |  |  | 1.3 (1.0-2.0) | 1.3 (1.0-1.7) | 0.38  (-0.00-0.77)^e^ |  |
|  | Min-Max | 1.0-4.0 | 1.0-3.3 |  |  |  |  |  |  |  |  |  |  |  |  | 1.0-4.0 | 1.0-4.0 |  |  |

|  | **Statistics** | **Control** | **Inter-vention** | **Control** | **Inter-vention** | **Treat-ment effect estimate (95% CI)** | **Sig.** | **Control** | **Inter-vention** | **Treat-ment effect estimate (95% CI)** | **Sig.** | **Control** | **Inter-vention** | **Treat-ment effect estimate (95% CI)** | **Sig.** | **Control** | **Inter-vention** | **Treat-ment effect estimate (95% CI)** | **Sig.** |
| --- | --- | --- | --- | --- | --- | --- | --- | --- | --- | --- | --- | --- | --- | --- | --- | --- | --- | --- | --- |
|  |  | *Baseline* | | *Week 1* | | | | *1 Month/ Week 5* | | | | *3 Month* | | | | *6 Month* | | | |
| Inattentiveness  (1-4) | n | 71 | 70 |  |  |  |  |  |  |  |  |  |  |  |  | 58 | 50 |  |  |
|  | Mean (s.d.) | 1.97 (0.66) | 2.05 (0.66) |  |  |  |  |  |  |  |  |  |  |  |  | 1.78 (0.65) | 1.49 (0.60) | 0.40 (0.20-0.80)^a^ | **0.0102** |
|  | Median (Q1-Q3) | 2.0 (1.7-2.3) | 2.0 (1.7-2.3) |  |  |  |  |  |  |  |  |  |  |  |  | 1.7 (1.3-2.0) | 1.3 (1.0-2.0) | 0.46 (0.07-0.85)^e^ |  |
|  | Min-Max | 1.0-3.7 | 1.0-4.0 |  |  |  |  |  |  |  |  |  |  |  |  | 1.0-3.7 | 1.0-3.3 |  |  |
| Total (1-4) | n | 71 | 70 |  |  |  |  |  |  |  |  |  |  |  |  | 58 | 50 |  |  |
|  | Mean (s.d.) | 1.98 (0.64) | 2.00 (0.63) |  |  |  |  |  |  |  |  |  |  |  |  | 1.80 (0.61) | 1.56 (0.58) | 0.46 (0.23-0.90)^a^ | **0.0240** |
|  | Median (Q1-Q3) | 2.0 (1.6-2.3) | 1.8 (1.6-2.4) |  |  |  |  |  |  |  |  |  |  |  |  | 1.7 (1.3-2.1) | 1.3 (1.1-1.9) | 0.40 (0.01-0.79)^e^ |  |
|  | Min-Max | 1.0-3.8 | 1.0-3.7 |  |  |  |  |  |  |  |  |  |  |  |  | 1.0-3.6 | 1.0-3.8 |  |  |
| Sick leave (total number of days) | n | 71 | 73 | 59 | 54 |  |  | 55 | 48 |  |  | 52 | 46 |  |  | 58 | 51 |  |  |
|  | Mean (s.d.) | 1.24 (3.25) | 1.15 (2.75) | 0.95 (4.12) | 0.26 (0.76) | 0.27 (0.02-1.44)^c^ | 0.1805 | 1.18 (2.84) | 0.67 (1.84) | 0.56 (0.18-1.53)^c^ | 0.2821 | 0.77 (2.39) | 0.24 (0.74) | 0.31 (0.05-1.20)^c^ | 0.1283 | 0.86 (2.90) | 0.41 (1.22) | 0.48 (0.10-1.69)^c^ | 0.2818 |
|  | Median (Q1-Q3) | 0.0 (0.0-0.0) | 0.0 (0.0-0.0) | 0.0 (0.0-0.0) | 0.0 (0.0-0.0) | 0.23  (-0.15-0.60)^e^ |  | 0.0 (0.0-0.0) | 0.0 (0.0-0.0) | 0.21  (-0.18-0.61)^e^ |  | 0.0 (0.0-0.0) | 0.0 (0.0-0.0) | 0.29  (-0.11-0.70)^e^ |  | 0.0 (0.0-0.0) | 0.0 (0.0-0.0) | 0.20  (-0.18-0.58)^e^ |  |
|  | Min-Max | 0.0-14.0 | 0.0-14.0 | 0.0-30.0 | 0.0-3.0 |  |  | 0.0-14.0 | 0.0-10.0 |  |  | 0.0-10.0 | 0.0-3.0 |  |  | 0.0-15.0 | 0.0-7.0 |  |  |

|  | **Statistics** | **Control** | **Inter-vention** | **Control** | **Inter-vention** | **Treat-ment effect estimate (95% CI)** | **Sig.** | **Control** | **Inter-vention** | **Treat-ment effect estimate (95% CI)** | **Sig.** | **Control** | **Inter-vention** | **Treat-ment effect estimate (95% CI)** | **Sig.** | **Control** | **Inter-vention** | **Treat-ment effect estimate (95% CI)** | **Sig.** |
| --- | --- | --- | --- | --- | --- | --- | --- | --- | --- | --- | --- | --- | --- | --- | --- | --- | --- | --- | --- |
|  |  | *Baseline* | | *Week 1* | | | | *1 Month/ Week 5* | | | | *3 Month* | | | | *6 Month* | | | |
| Sick leave during the last two weeks (total number of full workdays) | n | 71 | 73 | 59 | 54 |  |  | 55 | 48 |  |  | 52 | 46 |  |  | 58 | 51 |  |  |
|  | Mean (s.d.) | 1.69 (3.82) | 1.05 (2.89) | 1.12 (3.15) | 0.78 (2.23) | 0.70 (0.22-2.00)^c^ | 0.5077 | 1.60 (2.90) | 0.98 (2.41) | 0.61 (0.25-1.38)^c^ | 0.2520 | 0.90 (2.29) | 0.39 (1.24) | 0.43 (0.11-1.34)^c^ | 0.1759 | 1.16 (3.84) | 0.59 (1.39) | 0.51 (0.13-1.66)^c^ | 0.2893 |
|  | Median (Q1-Q3) | 0.0 (0.0-0.0) | 0.0 (0.0-0.0) | 0.0 (0.0-0.0) | 0.0 (0.0-0.0) | 0.12  (-0.25-0.50)^e^ |  | 0.0 (0.0-2.0) | 0.0 (0.0-1.0) | 0.23  (-0.16-0.62)^e^ |  | 0.0 (0.0-0.0) | 0.0 (0.0-0.0) | 0.27  (-0.13-0.68)^e^ |  | 0.0 (0.0-0.0) | 0.0 (0.0-0.0) | 0.19  (-0.19-0.57)^e^ |  |
|  | Min-Max | 0.0-14.0 | 0.0-14.0 | 0.0-20.0 | 0.0-13.0 |  |  | 0.0-12.0 | 0.0-14.0 |  |  | 0.0-10.0 | 0.0-7.0 |  |  | 0.0-21.0 | 0.0-7.0 |  |  |
| Stress and Energy Questionnaire (SEQ) - Stress Subscale (0-12) | n | 71 | 71 | 59 | 53 |  |  | 55 | 48 |  |  | 51 | 46 |  |  | 58 | 51 |  |  |
|  | Mean (s.d.) | 6.10 (3.65) | 6.08 (3.28) | 5.85 (3.85) | 4.51 (3.21) | 0.54 (0.28-1.04)^a^ | 0.0654 | 6.42 (3.53) | 4.25 (2.69) | 0.33 (0.16-0.66)^a^ | **0.0018** | 5.02 (3.62) | 3.76 (2.73) | 0.57 (0.28-1.15)^a^ | 0.1169 | 4.76 (3.55) | 3.57 (2.35) | 0.56 (0.29-1.09)^a^ | 0.0875 |
|  | Median (Q1-Q3) | 6.0 (3.0-9.0) | 6.0 (4.0-9.0) | 6.0 (3.0-9.0) | 4.0 (2.0-6.0) | 0.38  (-0.00-0.75)^e^ |  | 6.0 (3.0-9.0) | 4.0 (2.0-6.0) | 0.68 (0.28-1.09)^e^ |  | 4.0 (2.0-8.0) | 3.0 (2.0-5.0) | 0.39  (-0.02-0.80)^e^ |  | 4.5 (2.0-7.0) | 3.0 (2.0-5.0) | 0.39 (0.01-0.77)^e^ |  |
|  | Min-Max | 0.0-12.0 | 0.0-12.0 | 0.0-12.0 | 0.0-12.0 |  |  | 0.0-12.0 | 0.0-12.0 |  |  | 0.0-12.0 | 0.0-12.0 |  |  | 0.0-12.0 | 0.0-12.0 |  |  |

|  | **Statistics** | **Control** | **Inter-vention** | **Control** | **Inter-vention** | **Treat-ment effect estimate (95% CI)** | **Sig.** | **Control** | **Inter-vention** | **Treat-ment effect estimate (95% CI)** | **Sig.** | **Control** | **Inter-vention** | **Treat-ment effect estimate (95% CI)** | **Sig.** | **Control** | **Inter-vention** | **Treat-ment effect estimate (95% CI)** | **Sig.** |
| --- | --- | --- | --- | --- | --- | --- | --- | --- | --- | --- | --- | --- | --- | --- | --- | --- | --- | --- | --- |
|  |  | *Baseline* | | *Week 1* | | | | *1 Month/ Week 5* | | | | *3 Month* | | | | *6 Month* | | | |
| Moral stress at work (5-20)^0^ | n | 70 | 73 | 59 | 52 |  |  | 55 | 48 |  |  | 51 | 46 |  |  | 58 | 50 |  |  |
|  | Mean (s.d.) | 13.47 (3.99) | 13.00 (3.65) | 13.22 (4.00) | 14.23 (3.53) | 1.51 (0.79-2.90)^a^ | 0.2175 | 13.44 (3.81) | 15.21 (3.11) | 2.28 (1.15-4.57)^a^ | **0.0189** | 14.25 (3.53) | 14.65 (3.75) | 1.33 (0.67-2.69)^a^ | 0.4180 | 14.50 (3.54) | 14.88 (2.99) | 1.21 (0.62-2.34)^a^ | 0.5763 |
|  | Median (Q1-Q3) | 14.0 (11.0-16.0) | 13.0 (10.0-16.0) | 14.0 (10.0-16.0) | 14.0 (12.0-17.0) | -0.27  (-0.65-0.11)^e^ |  | 14.0 (11.0-16.0) | 15.0 (13.0-18.0) | -0.51  (-0.90-  -0.11)^e^ |  | 14.0 (11.0-18.0) | 15.0 (12.0-18.0) | -0.11  (-0.51-0.29)^e^ |  | 14.0 (12.0-18.0) | 14.0 (13.0-18.0) | -0.12  (-0.50-0.27)^e^ |  |
|  | Min-Max | 5.0-20.0 | 5.0-20.0 | 5.0-20.0 | 5.0-20.0 |  |  | 5.0-20.0 | 5.0-20.0 |  |  | 8.0-20.0 | 5.0-20.0 |  |  | 9.0-20.0 | 10.0-20.0 |  |  |
| Difficulties in letting go of work-related thoughts during leisure time (0-4)^1^ | n | 71 | 71 | 59 | 53 |  |  | 55 | 48 |  |  | 51 | 46 |  |  | 58 | 51 |  |  |
|  | Mean (s.d.) | 1.94 (1.19) | 1.90 (1.20) | 1.98 (1.25) | 2.34 (1.19) | 1.74 (0.89-3.42)^a^ | 0.1045 | 2.02 (1.30) | 2.35 (1.21) | 1.59 (0.80-3.19)^a^ | 0.1902 | 2.12 (1.28) | 2.65 (1.29) | 2.15 (1.05-4.47)^a^ | **0.0390** | 1.93 (1.23) | 2.51 (1.24) | 2.36 (1.19-4.75)^a^ | **0.0145** |
|  | Median (Q1-Q3) | 2.0 (1.0-3.0) | 2.0 (1.0-3.0) | 2.0 (1.0-3.0) | 3.0 (1.0-3.0) | -0.29  (-0.67-0.09)^e^ |  | 2.0 (1.0-3.0) | 2.5 (1.0-3.0) | -0.27  (-0.66-0.13)^e^ |  | 2.0 (1.0-3.0) | 3.0 (2.0-4.0) | -0.42  (-0.83-  -0.01)^e^ |  | 2.0 (1.0-3.0) | 3.0 (1.0-4.0) | -0.47  (-0.86-  -0.08)^e^ |  |
|  | Min-Max | 0.0-4.0 | 0.0-4.0 | 0.0-4.0 | 0.0-4.0 |  |  | 0.0-4.0 | 0.0-4.0 |  |  | 0.0-4.0 | 0.0-4.0 |  |  | 0.0-4.0 | 0.0-4.0 |  |  |
| Whether above mentioned difficulties are due to intrusive memories |  |  |  |  |  | 0.39 (0.18-0.84)^b^ | **0.0178** |  |  | 0.28 (0.10-0.70)^b^ | **0.0093** |  |  | 0.25 (0.08-0.67)^b^ | **0.0088** |  |  | 0.61 (0.23-1.53)^b^ | 0.3041 |
| YYes | n(%) | 40 (56.3) | 35 (49.3) | 31 (52.5) | 16 (30.2) |  |  | 21 (38.2) | 7 (14.6) |  |  | 19 (37.3) | 6 (13.0) |  |  | 15 (25.9) | 9 (17.6) |  |  |
| NNo | n(%) | 31 (43.7) | 36 (50.7) | 28 (47.5) | 37 (69.8) |  |  | 34 (61.8) | 41 (85.4) |  |  | 32 (62.7) | 40 (87.0) |  |  | 43 (74.1) | 42 (82.4) |  |  |

|  | **Statistics** | **Control** | **Inter-vention** | **Control** | **Inter-vention** | **Treat-ment effect estimate (95% CI)** | **Sig.** | **Control** | **Inter-vention** | **Treat-ment effect estimate (95% CI)** | **Sig.** | **Control** | **Inter-vention** | **Treat-ment effect estimate (95% CI)** | **Sig.** | **Control** | **Inter-vention** | **Treat-ment effect estimate (95% CI)** | **Sig.** |
| --- | --- | --- | --- | --- | --- | --- | --- | --- | --- | --- | --- | --- | --- | --- | --- | --- | --- | --- | --- |
|  |  | *Baseline* | | *Week 1* | | | | *1 Month/ Week 5* | | | | *3 Month* | | | | *6 Month* | | | |
| **Wellbeing** | | | | | | | | | | | | | | | | | | | |
| World Health Organization Disability Assessment Schedule 2.0 (WHODAS) | | | | | | | | | | | | | | | | | | | |
| Cognition (2-10) | n |  |  |  |  |  |  |  |  |  |  |  |  |  |  | 58 | 50 |  |  |
|  | Mean (s.d.) |  |  |  |  |  |  |  |  |  |  |  |  |  |  | 3.59 (1.55) | 3.16 (1.71) | 0.48 (0.23-0.96)^a^ | **0.0400** |
|  | Median (Q1-Q3) |  |  |  |  |  |  |  |  |  |  |  |  |  |  | 3.0 (2.0-5.0) | 2.0 (2.0-4.0) | 0.26  (-0.12-0.65)^e^ |  |
|  | Min-Max |  |  |  |  |  |  |  |  |  |  |  |  |  |  | 2.0-8.0 | 2.0-8.0 |  |  |
| Mobility (2-10) | n |  |  |  |  |  |  |  |  |  |  |  |  |  |  | 58 | 50 |  |  |
|  | Mean (s.d.) |  |  |  |  |  |  |  |  |  |  |  |  |  |  | 3.03 (1.57) | 2.70 (1.28) | 0.62 (0.29-1.30)^a^ | 0.2074 |
|  | Median (Q1-Q3) |  |  |  |  |  |  |  |  |  |  |  |  |  |  | 2.0 (2.0-3.0) | 2.0 (2.0-3.0) | 0.23  (-0.15-0.62)^e^ |  |
|  | Min-Max |  |  |  |  |  |  |  |  |  |  |  |  |  |  | 2.0-9.0 | 2.0-8.0 |  |  |
| Personal Care  (2-10) | n |  |  |  |  |  |  |  |  |  |  |  |  |  |  | 58 | 50 |  |  |
|  | Mean (s.d.) |  |  |  |  |  |  |  |  |  |  |  |  |  |  | 2.55 (1.16) | 2.20 (0.81) | 0.30 (0.08-0.91)^a^ | **0.0469** |
|  | Median (Q1-Q3) |  |  |  |  |  |  |  |  |  |  |  |  |  |  | 2.0 (2.0-2.0) | 2.0 (2.0-2.0) | 0.35  (-0.04-0.73)^e^ |  |
|  | Min-Max |  |  |  |  |  |  |  |  |  |  |  |  |  |  | 2.0-7.0 | 2.0-7.0 |  |  |

|  | **Statistics** | **Control** | **Inter-vention** | **Control** | **Inter-vention** | **Treat-ment effect estimate (95% CI)** | **Sig.** | **Control** | **Inter-vention** | **Treat-ment effect estimate (95% CI)** | **Sig.** | **Control** | **Inter-vention** | **Treat-ment effect estimate (95% CI)** | **Sig.** | **Control** | **Inter-vention** | **Treat-ment effect estimate (95% CI)** | **Sig.** |
| --- | --- | --- | --- | --- | --- | --- | --- | --- | --- | --- | --- | --- | --- | --- | --- | --- | --- | --- | --- |
|  |  | *Baseline* | | *Week 1* | | | | *1 Month/ Week 5* | | | | *3 Month* | | | | *6 Month* | | | |
| Relations (2-10) | n |  |  |  |  |  |  |  |  |  |  |  |  |  |  | 58 | 50 |  |  |
|  | Mean (s.d.) |  |  |  |  |  |  |  |  |  |  |  |  |  |  | 3.50 (1.73) | 3.28 (1.63) | 0.80 (0.40-1.60)^a^ | 0.5366 |
|  | Median (Q1-Q3) |  |  |  |  |  |  |  |  |  |  |  |  |  |  | 3.0 (2.0-4.0) | 3.0 (2.0-4.0) | 0.13  (-0.25-0.51)^e^ |  |
|  | Min-Max |  |  |  |  |  |  |  |  |  |  |  |  |  |  | 2.0-8.0 | 2.0-8.0 |  |  |
| Daily activities  (2-10) | n |  |  |  |  |  |  |  |  |  |  |  |  |  |  | 58 | 50 |  |  |
|  | Mean (s.d.) |  |  |  |  |  |  |  |  |  |  |  |  |  |  | 3.86 (1.98) | 3.34 (1.62) | 0.60 (0.30-1.20)^a^ | 0.1516 |
|  | Median (Q1-Q3) |  |  |  |  |  |  |  |  |  |  |  |  |  |  | 3.0 (2.0-5.0) | 3.0 (2.0-4.0) | 0.29  (-0.10-0.67)^e^ |  |
|  | Min-Max |  |  |  |  |  |  |  |  |  |  |  |  |  |  | 2.0-10.0 | 2.0-8.0 |  |  |
| Participation in society (2-10) | n |  |  |  |  |  |  |  |  |  |  |  |  |  |  | 58 | 50 |  |  |
|  | Mean (s.d.) |  |  |  |  |  |  |  |  |  |  |  |  |  |  | 4.93 (1.94) | 4.54 (2.15) | 0.72 (0.37-1.41)^a^ | 0.3369 |
|  | Median (Q1-Q3) |  |  |  |  |  |  |  |  |  |  |  |  |  |  | 5.0 (3.0-6.0) | 4.5 (2.0-6.0) | 0.19  (-0.19-0.58)^e^ |  |
|  | Min-Max |  |  |  |  |  |  |  |  |  |  |  |  |  |  | 2.0-9.0 | 2.0-9.0 |  |  |

|  | **Statistics** | **Control** | **Inter-vention** | **Control** | **Inter-vention** | **Treat-ment effect estimate (95% CI)** | **Sig.** | **Control** | **Inter-vention** | **Treat-ment effect estimate (95% CI)** | **Sig.** | **Control** | **Inter-vention** | **Treat-ment effect estimate (95% CI)** | **Sig.** | **Control** | **Inter-vention** | **Treat-ment effect estimate (95% CI)** | **Sig.** |
| --- | --- | --- | --- | --- | --- | --- | --- | --- | --- | --- | --- | --- | --- | --- | --- | --- | --- | --- | --- |
|  |  | *Baseline* | | *Week 1* | | | | *1 Month/ Week 5* | | | | *3 Month* | | | | *6 Month* | | | |
| WHODAS Disability Score (12-60) | n |  |  |  |  |  |  |  |  |  |  |  |  |  |  | 58 | 50 |  |  |
|  | Mean (s.d.) |  |  |  |  |  |  |  |  |  |  |  |  |  |  | 21.47 (7.86) | 19.22 (7.57) | 0.55 (0.28-1.08)^a^ | 0.0826 |
|  | Median (Q1-Q3) |  |  |  |  |  |  |  |  |  |  |  |  |  |  | 19.0 (16.0-27.0) | 17.5 (13.0-23.0) | 0.29  (-0.09-0.68)^e^ |  |
|  | Min-Max |  |  |  |  |  |  |  |  |  |  |  |  |  |  | 12.0-44.0 | 12.0-44.0 |  |  |

|  | **Statistics** | **Control** | **Inter-vention** | **Control** | **Inter-vention** | **Treat-ment effect estimate (95% CI)** | **Sig.** | **Control** | **Inter-vention** | **Treat-ment effect estimate (95% CI)** | **Sig.** | **Control** | **Inter-vention** | **Treat-ment effect estimate (95% CI)** | **Sig.** | **Control** | **Inter-vention** | **Treat-ment effect estimate (95% CI)** | **Sig.** |
| --- | --- | --- | --- | --- | --- | --- | --- | --- | --- | --- | --- | --- | --- | --- | --- | --- | --- | --- | --- |
|  |  | *Baseline* | | *Week 1* | | | | *1 Month/ Week 5* | | | | *3 Month* | | | | *6 Month* | | | |
| How many of the past 7 days did you have these difficulties? | n |  |  |  |  |  |  |  |  |  |  |  |  |  |  | 58 | 50 |  |  |
|  | Mean (s.d.) |  |  |  |  |  |  |  |  |  |  |  |  |  |  | 1.97 (2.41) | 0.88 (1.85) | 0.31 (0.14-0.66)^a^ | **0.0027** |
|  | Median (Q1-Q3) |  |  |  |  |  |  |  |  |  |  |  |  |  |  | 1.0 (0.0-3.0) | 0.0 (0.0-1.0) | 0.50 (0.11-0.89)^e^ |  |
|  | Min-Max |  |  |  |  |  |  |  |  |  |  |  |  |  |  | 0.0-7.0 | 0.0-7.0 |  |  |
| How many of the past 7 days were you completely unable to carry out your usual activities or work because of your health condition? | n |  |  |  |  |  |  |  |  |  |  |  |  |  |  | 58 | 50 |  |  |
|  | Mean (s.d.) |  |  |  |  |  |  |  |  |  |  |  |  |  |  | 0.50 (1.52) | 0.16 (0.74) | 0.46 (0.12-1.51)^a^ | 0.2182 |
|  | Median (Q1-Q3) |  |  |  |  |  |  |  |  |  |  |  |  |  |  | 0.0 (0.0-0.0) | 0.0 (0.0-0.0) | 0.28  (-0.11-0.66)^e^ |  |
|  | Min-Max |  |  |  |  |  |  |  |  |  |  |  |  |  |  | 0.0-7.0 | 0.0-5.0 |  |  |

|  | **Statistics** | **Control** | **Inter-vention** | **Control** | **Inter-vention** | **Treat-ment effect estimate (95% CI)** | **Sig.** | **Control** | **Inter-vention** | **Treat-ment effect estimate (95% CI)** | **Sig.** | **Control** | **Inter-vention** | **Treat-ment effect estimate (95% CI)** | **Sig.** | **Control** | **Inter-vention** | **Treat-ment effect estimate (95% CI)** | **Sig.** |
| --- | --- | --- | --- | --- | --- | --- | --- | --- | --- | --- | --- | --- | --- | --- | --- | --- | --- | --- | --- |
|  |  | *Baseline* | | *Week 1* | | | | *1 Month/ Week 5* | | | | *3 Month* | | | | *6 Month* | | | |
| How many of the past 7 days did you cut back or reduce your usual activities or work because of your health condition? | n |  |  |  |  |  |  |  |  |  |  |  |  |  |  | 58 | 50 |  |  |
|  | Mean (s.d.) |  |  |  |  |  |  |  |  |  |  |  |  |  |  | 1.64 (2.50) | 0.64 (1.50) | 0.46 (0.21-1.01)^a^ | 0.0565 |
|  | Median (Q1-Q3) |  |  |  |  |  |  |  |  |  |  |  |  |  |  | 0.0 (0.0-3.0) | 0.0 (0.0-1.0) | 0.48 (0.09-0.86)^e^ |  |
|  | Min-Max |  |  |  |  |  |  |  |  |  |  |  |  |  |  | 0.0-7.0 | 0.0-7.0 |  |  |
| Self Rated Health (SRH) (0-6) | n | 71 | 73 | 60 | 54 |  |  | 55 | 50 |  |  | 53 | 46 |  |  | 58 | 52 |  |  |
|  | Mean (s.d.) | 4.08 (1.16) | 4.10 (1.22) | 3.95 (1.05) | 4.44 (1.18) | 2.61 (1.32-5.26)^a^ | **0.0066** | 3.89 (1.52) | 4.68 (1.19) | 2.84 (1.41-5.87)^a^ | **0.0041** | 4.11 (1.07) | 4.67 (1.17) | 3.13 (1.48-6.80)^a^ | **0.0033** | 4.10 (1.17) | 4.67 (1.22) | 2.82 (1.40-5.82)^a^ | **0.0041** |
|  | Median (Q1-Q3) | 4.0 (3.0-5.0) | 4.0 (3.0-5.0) | 4.0 (3.0-5.0) | 5.0 (4.0-5.0) | -0.45  (-0.82-  -0.07)^e^ |  | 4.0 (3.0-5.0) | 5.0 (4.0-5.0) | -0.57  (-0.97-  -0.18)^e^ |  | 4.0 (3.0-5.0) | 5.0 (4.0-5.0) | -0.50  (-0.91-  -0.09)^e^ |  | 4.0 (4.0-5.0) | 5.0 (4.0-5.5) | -0.48  (-0.86-  -0.09)^e^ |  |
|  | Min-Max | 1.0-6.0 | 1.0-6.0 | 1.0-6.0 | 1.0-6.0 |  |  | 0.0-6.0 | 1.0-6.0 |  |  | 2.0-6.0 | 0.0-6.0 |  |  | 1.0-6.0 | 1.0-6.0 |  |  |

|  | **Statistics** | **Control** | **Inter-vention** | **Control** | **Inter-vention** | **Treat-ment effect estimate (95% CI)** | **Sig.** | **Control** | **Inter-vention** | **Treat-ment effect estimate (95% CI)** | **Sig.** | **Control** | **Inter-vention** | **Treat-ment effect estimate (95% CI)** | **Sig.** | **Control** | **Inter-vention** | **Treat-ment effect estimate (95% CI)** | **Sig.** |
| --- | --- | --- | --- | --- | --- | --- | --- | --- | --- | --- | --- | --- | --- | --- | --- | --- | --- | --- | --- |
|  |  | *Baseline* | | *Week 1* | | | | *1 Month/ Week 5* | | | | *3 Month* | | | | *6 Month* | | | |
| Self-rated sleep ratings (SCI-02) (0-8)^2^ | n | 71 | 73 | 60 | 54 |  |  | 55 | 50 |  |  | 53 | 46 |  |  | 58 | 52 |  |  |
|  | Mean (s.d.) | 4.14 (2.63) | 3.97 (2.53) | 4.92 (2.30) | 5.89 (2.45) | 2.61 (1.34-5.17)^a^ | **0.0053** | 4.65 (2.82) | 6.02 (2.10) | 2.16 (1.09-4.33)^a^ | **0.0293** | 4.77 (2.33) | 6.28 (2.05) | 3.92 (1.88-8.38)^a^ | **0.0003** | 5.40 (2.41) | 6.23 (1.83) | 1.85 (0.95-3.64)^a^ | 0.0742 |
|  | Median (Q1-Q3) | 4.0 (2.0-7.0) | 4.0 (2.0-6.0) | 6.0 (3.0-6.5) | 7.0 (5.0-8.0) | -0.41  (-0.78-  -0.03)^e^ |  | 5.0 (2.0-7.0) | 7.0 (5.0-7.0) | -0.54  (-0.94-  -0.15)^e^ |  | 5.0 (3.0-7.0) | 7.0 (6.0-8.0) | -0.68  (-1.10-  -0.27)^e^ |  | 6.0 (4.0-7.0) | 7.0 (6.0-7.0) | -0.39  (-0.77-  -0.00)^e^ |  |
|  | Min-Max | 0.0-8.0 | 0.0-8.0 | 0.0-8.0 | 0.0-8.0 |  |  | 0.0-8.0 | 0.0-8.0 |  |  | 0.0-8.0 | 0.0-8.0 |  |  | 0.0-8.0 | 0.0-8.0 |  |  |
| Social support after traumatic event (1-10)^3^ | n | 71 | 73 | 61 | 54 |  |  | 55 | 50 |  |  | 55 | 47 |  |  | 58 | 51 |  |  |
|  | Mean (s.d.) | 6.39 (2.73) | 6.01 (3.02) | 5.98 (2.76) | 6.81 (2.43) | 1.65 (0.87-3.16)^a^ | 0.1265 | 5.96 (2.77) | 6.22 (3.25) | 1.25 (0.64-2.47)^a^ | 0.5118 | 6.09 (3.12) | 5.85 (3.24) | 0.89 (0.45-1.76)^a^ | 0.7380 | 5.48 (3.12) | 5.92 (2.83) | 1.29 (0.67-2.50)^a^ | 0.4460 |
|  | Median (Q1-Q3) | 7.0 (4.0-9.0) | 6.0 (3.0-8.0) | 6.0 (3.0-8.0) | 7.0 (5.0-9.0) | -0.32  (-0.69-0.05)^e^ |  | 7.0 (3.0-8.0) | 7.0 (4.0-9.0) | -0.09  (-0.47-0.30)^e^ |  | 7.0 (4.0-9.0) | 6.0 (3.0-9.0) | 0.08  (-0.32-0.47)^e^ |  | 5.0 (3.0-8.0) | 6.0 (4.0-8.0) | -0.15  (-0.53-0.23)^e^ |  |
|  | Min-Max | 1.0-10.0 | 1.0-10.0 | 1.0-10.0 | 1.0-10.0 |  |  | 1.0-10.0 | 1.0-10.0 |  |  | 1.0-10.0 | 1.0-10.0 |  |  | 1.0-10.0 | 1.0-10.0 |  |  |

|  | **Statistics** | **Control** | **Inter-vention** | **Control** | **Inter-vention** | **Treat-ment effect estimate (95% CI)** | **Sig.** | **Control** | **Inter-vention** | **Treat-ment effect estimate (95% CI)** | **Sig.** | **Control** | **Inter-vention** | **Treat-ment effect estimate (95% CI)** | **Sig.** | **Control** | **Inter-vention** | **Treat-ment effect estimate (95% CI)** | **Sig.** |
| --- | --- | --- | --- | --- | --- | --- | --- | --- | --- | --- | --- | --- | --- | --- | --- | --- | --- | --- | --- |
|  |  | *Baseline* | | *Week 1* | | | | *1 Month/ Week 5* | | | | *3 Month* | | | | *6 Month* | | | |
| **Intrusion memory ratings** | | | | | | | | | | | | | | | | | | | |
| Concentration disruption (diary, week 0, 1, 5) | | | | | | | | | | | | | | | | | | | |
| Level Diary week (0-10) | n | 69 | 68 | 57 | 51 |  |  | 46 | 27 |  |  |  |  |  |  |  |  |  |  |
|  | Mean (s.d.) | 3.55 (2.15) | 3.85 (2.63) | 2.77 (2.06) | 1.92 (2.23) | 0.40 (0.20-0.79)^a^ | **0.0092** | 2.67 (2.31) | 1.78 (2.26) | 0.39 (0.16-0.91)^a^ | **0.0321** |  |  |  |  |  |  |  |  |
|  | Median (Q1-Q3) | 3.0 (2.0-5.0) | 3.0 (2.0-6.0) | 3.0 (1.0-4.0) | 1.0 (0.0-3.0) | 0.40 (0.01-0.78)^e^ |  | 2.0 (1.0-4.0) | 1.0 (0.0-2.0) | 0.39  (-0.10-0.88)^e^ |  |  |  |  |  |  |  |  |  |
|  | Min-Max | 0.0-9.0 | 0.0-10.0 | 0.0-9.0 | 0.0-10.0 |  |  | 0.0-9.0 | 0.0-10.0 |  |  |  |  |  |  |  |  |  |  |
| Duration  (0-5) | n | 69 | 68 | 57 | 51 |  |  | 46 | 27 |  |  |  |  |  |  |  |  |  |  |
|  | Mean (s.d.) | 1.10 (1.09) | 1.25 (0.89) | 0.98 (1.08) | 0.67 (0.77) | 0.60 (0.29-1.22)^a^ | 0.1619 | 1.02 (1.16) | 0.85 (1.03) | 0.75 (0.30-1.83)^a^ | 0.5294 |  |  |  |  |  |  |  |  |
|  | Median (Q1-Q3) | 1.0 (0.0-1.0) | 1.0 (1.0-2.0) | 1.0 (0.0-1.0) | 1.0 (0.0-1.0) | 0.33  (-0.05-0.72)^e^ |  | 1.0 (0.0-1.0) | 1.0 (0.0-1.0) | 0.15  (-0.33-0.64)^e^ |  |  |  |  |  |  |  |  |  |
|  | Min-Max | 0.0-4.0 | 0.0-4.0 | 0.0-4.0 | 0.0-3.0 |  |  | 0.0-5.0 | 0.0-4.0 |  |  |  |  |  |  |  |  |  |  |

|  | **Statistics** | **Control** | **Inter-vention** | **Control** | **Inter-vention** | **Treat-ment effect estimate (95% CI)** | **Sig.** | **Control** | **Inter-vention** | **Treat-ment effect estimate (95% CI)** | **Sig.** | **Control** | **Inter-vention** | **Treat-ment effect estimate (95% CI)** | **Sig.** | **Control** | **Inter-vention** | **Treat-ment effect estimate (95% CI)** | **Sig.** |
| --- | --- | --- | --- | --- | --- | --- | --- | --- | --- | --- | --- | --- | --- | --- | --- | --- | --- | --- | --- |
|  |  | *Baseline* | | *Week 1* | | | | *1 Month/ Week 5* | | | | *3 Month* | | | | *6 Month* | | | |
| Concentration and memory difficulties – 11 item scale (11-55)^4^ | n |  |  |  |  |  |  |  |  |  |  |  |  |  |  | 52 | 42 |  |  |
|  | Mean (s.d.) |  |  |  |  |  |  |  |  |  |  |  |  |  |  | 38.17 (10.84) | 44.55 (7.93) | 3.36 (1.62-7.14)^a^ | **0.0013** |
|  | Median (Q1-Q3) |  |  |  |  |  |  |  |  |  |  |  |  |  |  | 40.5 (32.0-47.0) | 46.0 (42.0-50.0) | -0.66  (-1.08-  -0.24)^e^ |  |
|  | Min-Max |  |  |  |  |  |  |  |  |  |  |  |  |  |  | 11.0-54.0 | 15.0-53.0 |  |  |
| Self-rated impact of IMs on occupational functioning (0-10) | n | 71 | 73 | 61 | 54 |  |  | 55 | 50 |  |  | 55 | 47 |  |  | 58 | 51 |  |  |
|  | Mean (s.d.) | 2.58 (2.32) | 2.52 (2.32) | 2.69 (2.74) | 1.72 (1.96) | 0.56 (0.29-1.07)^a^ | 0.0816 | 2.96 (2.66) | 1.20 (1.97) | 0.22 (0.10-0.45)^a^ | <0.0001 | 2.20 (2.36) | 0.87 (1.60) | 0.30 (0.14-0.63)^a^ | 0.0016 | 2.10 (2.11) | 1.06 (1.87) | 0.28 (0.13-0.56)^a^ | **0.0005** |
|  | Median (Q1-Q3) | 2.0 (1.0-5.0) | 2.0 (1.0-4.0) | 1.0 (1.0-5.0) | 1.0 (0.0-3.0) | 0.40 (0.03-0.78)^e^ |  | 2.0 (1.0-5.0) | 0.0 (0.0-2.0) | 0.75 (0.35-1.15)^e^ |  | 1.0 (0.0-4.0) | 0.0 (0.0-1.0) | 0.65 (0.25-1.05)^e^ |  | 1.0 (1.0-3.0) | 0.0 (0.0-1.0) | 0.52 (0.14-0.91)^e^ |  |
|  | Min-Max | 0.0-9.0 | 0.0-10.0 | 0.0-9.0 | 0.0-8.0 |  |  | 0.0-10.0 | 0.0-9.0 |  |  | 0.0-8.0 | 0.0-9.0 |  |  | 0.0-8.0 | 0.0-9.0 |  |  |

|  | **Statistics** | **Control** | **Inter-vention** | **Control** | **Inter-vention** | **Treat-ment effect estimate (95% CI)** | **Sig.** | **Control** | **Inter-vention** | **Treat-ment effect estimate (95% CI)** | **Sig.** | **Control** | **Inter-vention** | **Treat-ment effect estimate (95% CI)** | **Sig.** | **Control** | **Inter-vention** | **Treat-ment effect estimate (95% CI)** | **Sig.** |
| --- | --- | --- | --- | --- | --- | --- | --- | --- | --- | --- | --- | --- | --- | --- | --- | --- | --- | --- | --- |
|  |  | *Baseline* | | *Week 1* | | | | *1 Month/ Week 5* | | | | *3 Month* | | | | *6 Month* | | | |
| Self-rated impact of IMs on daily functioning in other areas (1-10)^5^ | n | 71 | 73 | 61 | 54 |  |  | 55 | 50 |  |  | 55 | 47 |  |  | 58 | 51 |  |  |
|  | Mean (s.d.) | 3.93 (2.46) | 3.93 (2.61) | 4.03 (2.71) | 2.85 (2.10) | 0.47 (0.24-0.90)^a^ | 0.0243 | 3.91 (2.41) | 2.42 (2.22) | 0.24 (0.11-0.49)^a^ | 0.0001 | 3.51 (2.46) | 1.89 (1.66) | 0.23 (0.11-0.49)^a^ | 0.0002 | 3.17 (2.19) | 2.02 (1.87) | 0.26 (0.12-0.53)^a^ | **0.0003** |
|  | Median (Q1-Q3) | 4.0 (2.0-6.0) | 3.0 (2.0-6.0) | 4.0 (1.0-6.0) | 2.0 (1.0-4.0) | 0.48 (0.11-0.86)^e^ |  | 3.0 (2.0-6.0) | 1.0 (1.0-3.0) | 0.64 (0.24-1.04)^e^ |  | 3.0 (1.0-6.0) | 1.0 (1.0-2.0) | 0.76 (0.35-1.17)^e^ |  | 2.5 (1.0-4.0) | 1.0 (1.0-2.0) | 0.56 (0.17-0.95)^e^ |  |
|  | Min-Max | 1.0-10.0 | 1.0-10.0 | 1.0-10.0 | 1.0-10.0 |  |  | 1.0-10.0 | 1.0-10.0 |  |  | 1.0-9.0 | 1.0-10.0 |  |  | 1.0-8.0 | 1.0-9.0 |  |  |
| Perceived impact of intrusive memories on functioning | | | | | | | | | | | | | | | | | | | |
| Diary week 0, 1, 5 (0-10) | | | | | | | | | | | | | | | | | | | |
| Distress | n | 69 | 68 | 57 | 51 |  |  | 44 | 28 |  |  |  |  |  |  |  |  |  |  |
|  | Mean (s.d.) | 3.97 (2.07) | 4.25 (2.37) | 3.23 (2.26) | 2.61 (2.37) | 0.56 (0.29-1.10)^a^ | 0.0952 | 3.14 (2.43) | 2.04 (1.75) | 0.46 (0.20-1.06)^a^ | 0.0708 |  |  |  |  |  |  |  |  |
|  | Median (Q1-Q3) | 4.0 (2.0-6.0) | 4.0 (3.0-5.5) | 3.0 (2.0-5.0) | 2.0 (1.0-3.0) | 0.27  (-0.12-0.65)^e^ |  | 3.0 (1.0-5.5) | 2.0 (1.0-3.0) | 0.50 (0.01-0.99)^e^ |  |  |  |  |  |  |  |  |  |
|  | Min-Max | 0.0-8.0 | 0.0-10.0 | 0.0-10.0 | 0.0-10.0 |  |  | 0.0-9.0 | 0.0-7.0 |  |  |  |  |  |  |  |  |  |  |
| Vividness | n | 68 | 68 | 56 | 51 |  |  | 47 | 28 |  |  |  |  |  |  |  |  |  |  |
|  | Mean (s.d.) | 4.56 (2.54) | 4.26 (2.37) | 3.68 (2.31) | 3.08 (2.59) | 0.57 (0.29-1.12)^a^ | 0.1055 | 3.26 (2.33) | 2.75 (2.58) | 0.56 (0.24-1.30)^a^ | 0.1793 |  |  |  |  |  |  |  |  |
|  | Median (Q1-Q3) | 4.0 (3.0-6.0) | 4.0 (3.0-6.0) | 3.5 (2.0-5.0) | 3.0 (1.0-5.0) | 0.25  (-0.14-0.63)^e^ |  | 3.0 (2.0-4.0) | 2.0 (1.0-4.5) | 0.21  (-0.27-0.69)^e^ |  |  |  |  |  |  |  |  |  |
|  | Min-Max | 0.0-10.0 | 0.0-10.0 | 0.0-10.0 | 0.0-10.0 |  |  | 0.0-10.0 | 0.0-10.0 |  |  |  |  |  |  |  |  |  |  |

|  | **Statistics** | **Control** | **Inter-vention** | **Control** | **Inter-vention** | **Treat-ment effect estimate (95% CI)** | **Sig.** | **Control** | **Inter-vention** | **Treat-ment effect estimate (95% CI)** | **Sig.** | **Control** | **Inter-vention** | **Treat-ment effect estimate (95% CI)** | **Sig.** | **Control** | **Inter-vention** | **Treat-ment effect estimate (95% CI)** | **Sig.** |
| --- | --- | --- | --- | --- | --- | --- | --- | --- | --- | --- | --- | --- | --- | --- | --- | --- | --- | --- | --- |
|  |  | *Baseline* | | *Week 1* | | | | *1 Month/ Week 5* | | | | *3 Month* | | | | *6 Month* | | | |
| Concentration disruption (1, 3 and 6 month in follow-up questionnaires) | | | | | | | | | | | | | | | | | | | |
| Level questionnaire (0-10) | n |  |  |  |  |  |  | 45 | 29 |  |  | 44 | 20 |  |  | 49 | 26 |  |  |
|  | Mean (s.d.) |  |  |  |  |  |  | 2.98 (2.47) | 2.24 (2.26) | 0.55 (0.24-1.27)^a^ | 0.1661 | 2.14 (2.25) | 1.35 (2.13) | 0.46 (0.17-1.21)^a^ | 0.1201 | 2.27 (2.35) | 2.04 (2.24) | 0.90 (0.39-2.06)^a^ | 0.7998 |
|  | Median (Q1-Q3) |  |  |  |  |  |  | 2.0 (1.0-5.0) | 2.0 (0.0-4.0) | 0.31  (-0.17-0.79)^e^ |  | 1.5 (0.0-3.5) | 0.5 (0.0-2.0) | 0.36  (-0.19-0.90)^e^ |  | 2.0 (0.0-4.0) | 1.0 (1.0-3.0) | 0.10  (-0.39-0.58)^e^ |  |
|  | Min-Max |  |  |  |  |  |  | 0.0-9.0 | 0.0-7.0 |  |  | 0.0-7.0 | 0.0-9.0 |  |  | 0.0-9.0 | 0.0-9.0 |  |  |
| **Work situation** | | | | | | | | | | | | | | | | | | | |
| Question related to work situation | | | | | | | | | | | | | | | | | | | |
| Whether the work situation changed since study start^6^ |  |  |  |  |  | 1.45 (0.55-3.91)^b^ | 0.4531 |  |  | 0.98 (0.40-2.38)^b^ | 0.9578 |  |  | 0.65 (0.26-1.56)^b^ | 0.3385 |  |  | 0.95 (0.43-2.10)^b^ | 0.8994 |
| YYes | n(%) | 52 (74.3) | 60 (82.2) | 9 (15.0) | 11 (20.4) |  |  | 14 (25.5) | 12 (25.0) |  |  | 17 (32.7) | 11 (23.9) |  |  | 20 (34.5) | 17 (33.3) |  |  |
| NNo | n(%) | 18 (25.7) | 13 (17.8) | 51 (85.0) | 43 (79.6) |  |  | 41 (74.5) | 36 (75.0) |  |  | 35 (67.3) | 35 (76.1) |  |  | 38 (65.5) | 34 (66.7) |  |  |
| If yes, how^7^: |  |  |  |  |  |  | 0.2087^d^ |  |  |  | 0.0755^d^ |  |  |  | 0.2412^d^ |  |  |  | 0.8586^d^ |
| Changed job | n(%) | 9 (17.6) | 4 (7.0) | 1 (11.1) | 4 (44.4) |  |  | 0 | 3 (25.0) |  |  | 3 (18.8) | 4 (36.4) |  |  | 8 (44.4) | 8 (47.1) |  |  |
| Left profession/stopped working | n(%) | 2 (3.9) | 5 (8.8) | 1 (11.1) | 0 |  |  | 2 (15.4) | 0 |  |  | 3 (18.8) | 0 |  |  | 2 (11.1) | 1 (5.9) |  |  |
| Other | n(%) | 40 (78.4) | 48 (84.2) | 7 (77.8) | 5 (55.6) |  |  | 11 (84.6) | 9 (75.0) |  |  | 10 (62.5) | 7 (63.6) |  |  | 8 (44.4) | 8 (47.1) |  |  |

|  | **Statistics** | **Control** | **Inter-vention** | **Control** | **Inter-vention** | **Treat-ment effect estimate (95% CI)** | **Sig.** | **Control** | **Inter-vention** | **Treat-ment effect estimate (95% CI)** | **Sig.** | **Control** | **Inter-vention** | **Treat-ment effect estimate (95% CI)** | **Sig.** | **Control** | **Inter-vention** | **Treat-ment effect estimate (95% CI)** | **Sig.** |
| --- | --- | --- | --- | --- | --- | --- | --- | --- | --- | --- | --- | --- | --- | --- | --- | --- | --- | --- | --- |
|  |  | *Baseline* | | *Week 1* | | | | *1 Month/ Week 5* | | | | *3 Month* | | | | *6 Month* | | | |
| **Other cognitive assessments** | | | | | | | | | | | | | | | | | | | |
| Appraisals of IMs (0-300) | | | | | | | | | | | | | | | | | | | |
| Psychological problems | n | 71 | 73 | 60 | 54 |  |  | 55 | 50 |  |  | 54 | 47 |  |  | 58 | 51 |  |  |
|  | Mean (s.d.) | 50.56 (62.93) | 46.03 (53.69) | 46.83 (60.32) | 25.19 (28.47) | 0.73 (0.38-1.42)^a^ | 0.3601 | 55.45 (73.56) | 19.60 (29.20) | 0.37 (0.18-0.74)^a^ | **0.0059** | 49.44 (65.28) | 20.85 (42.83) | 0.35 (0.16-0.74)^a^ | **0.0068** | 49.83 (60.39) | 18.43 (42.54) | 0.23 (0.11-0.49)^a^ | **0.0002** |
|  | Median (Q1-Q3) | 20.0 (0.0-90.0) | 20.0 (0.0-80.0) | 15.0 (0.0-90.0) | 20.0 (0.0-40.0) | 0.45 (0.08-0.83)^e^ |  | 20.0 (0.0-90.0) | 0.0 (0.0-30.0) | 0.63 (0.23-1.03)^e^ |  | 20.0 (0.0-80.0) | 0.0 (0.0-30.0) | 0.51 (0.11-0.91)^e^ |  | 25.0 (0.0-80.0) | 0.0 (0.0-20.0) | 0.59 (0.21-0.98)^e^ |  |
|  | Min-Max | 0.0-230.0 | 0.0-240.0 | 0.0-220.0 | 0.0-130.0 |  |  | 0.0-240.0 | 0.0-90.0 |  |  | 0.0-270.0 | 0.0-210.0 |  |  | 0.0-260.0 | 0.0-210.0 |  |  |
| Negative self-evaluation | n | 71 | 73 | 60 | 54 |  |  | 55 | 50 |  |  | 54 | 47 |  |  | 58 | 51 |  |  |
|  | Mean (s.d.) | 47.32 (74.87) | 37.81 (54.24) | 34.83 (69.98) | 20.19 (47.68) | 0.88 (0.42-1.83)^a^ | 0.7312 | 43.82 (81.89) | 20.00 (50.79) | 0.62 (0.28-1.37)^a^ | 0.2423 | 35.74 (74.49) | 17.87 (49.30) | 0.74 (0.31-1.70)^a^ | 0.4756 | 45.69 (80.46) | 15.88 (47.04) | 0.45 (0.20-1.00)^a^ | 0.0540 |
|  | Median (Q1-Q3) | 0.0 (0.0-80.0) | 20.0 (0.0-60.0) | 0.0 (0.0-40.0) | 0.0 (0.0-20.0) | 0.24  (-0.13-0.62)^e^ |  | 0.0 (0.0-40.0) | 0.0 (0.0-10.0) | 0.35  (-0.04-0.74)^e^ |  | 0.0 (0.0-30.0) | 0.0 (0.0-10.0) | 0.28  (-0.12-0.68)^e^ |  | 0.0 (0.0-50.0) | 0.0 (0.0-10.0) | 0.45 (0.06-0.83)^e^ |  |
|  | Min-Max | 0.0-270.0 | 0.0-270.0 | 0.0-300.0 | 0.0-290.0 |  |  | 0.0-300.0 | 0.0-290.0 |  |  | 0.0-300.0 | 0.0-300.0 |  |  | 0.0-270.0 | 0.0-300.0 |  |  |

|  | **Statistics** | **Control** | **Inter-vention** | **Control** | **Inter-vention** | **Treat-ment effect estimate (95% CI)** | **Sig.** | **Control** | **Inter-vention** | **Treat-ment effect estimate (95% CI)** | **Sig.** | **Control** | **Inter-vention** | **Treat-ment effect estimate (95% CI)** | **Sig.** | **Control** | **Inter-vention** | **Treat-ment effect estimate (95% CI)** | **Sig.** |
| --- | --- | --- | --- | --- | --- | --- | --- | --- | --- | --- | --- | --- | --- | --- | --- | --- | --- | --- | --- |
|  |  | *Baseline* | | *Week 1* | | | | *1 Month/ Week 5* | | | | *3 Month* | | | | *6 Month* | | | |
| Time perspective questionnaire (TPQ) | | | | | | | | | | | | | | | | | | | |
| Past (3-15) | n | 71 | 72 | 59 | 53 |  |  | 55 | 47 |  |  | 51 | 46 |  |  | 58 | 50 |  |  |
|  | Mean (s.d.) | 9.42 (3.71) | 9.38 (3.03) | 9.29 (3.37) | 8.19 (2.78) | 0.51 (0.26-0.97)^a^ | **0.0421** | 9.13 (3.44) | 7.85 (3.30) | 0.53 (0.26-1.04)^a^ | 0.0662 | 8.82 (3.71) | 6.67 (2.66) | 0.36 (0.17-0.72)^a^ | **0.0047** | 8.00 (3.47) | 6.62 (2.48) | 0.50 (0.25-0.97)^a^ | **0.0410** |
|  | Median (Q1-Q3) | 9.0 (6.0-13.0) | 9.5 (7.0-11.5) | 10.0 (7.0-12.0) | 8.0 (6.0-10.0) | 0.35  (-0.02-0.73)^e^ |  | 9.0 (6.0-12.0) | 7.0 (5.0-10.0) | 0.38  (-0.02-0.78)^e^ |  | 8.0 (6.0-12.0) | 6.0 (5.0-8.0) | 0.66 (0.25-1.07)^e^ |  | 8.0 (5.0-10.0) | 6.0 (5.0-8.0) | 0.45 (0.06-0.84)^e^ |  |
|  | Min-Max | 3.0-15.0 | 3.0-15.0 | 3.0-15.0 | 3.0-15.0 |  |  | 3.0-15.0 | 3.0-15.0 |  |  | 3.0-15.0 | 3.0-15.0 |  |  | 3.0-15.0 | 3.0-14.0 |  |  |
| Present (3-10) | n | 71 | 72 | 59 | 53 |  |  | 55 | 47 |  |  | 51 | 46 |  |  | 58 | 50 |  |  |
|  | Mean (s.d.) | 6.15 (2.46) | 6.50 (2.53) | 6.25 (2.13) | 6.15 (2.25) | 0.89 (0.47-1.71)^a^ | 0.7335 | 6.42 (2.17) | 6.34 (2.27) | 0.97 (0.49-1.93)^a^ | 0.9402 | 6.39 (2.10) | 6.28 (2.46) | 0.85 (0.42-1.71)^a^ | 0.6430 | 6.12 (2.39) | 5.86 (2.31) | 0.83 (0.43-1.61)^a^ | 0.5846 |
|  | Median (Q1-Q3) | 6.0 (4.0-8.0) | 6.0 (4.5-9.0) | 6.0 (5.0-8.0) | 6.0 (4.0-8.0) | 0.05  (-0.33-0.42)^e^ |  | 6.0 (5.0-8.0) | 6.0 (5.0-8.0) | 0.04  (-0.36-0.43)^e^ |  | 6.0 (5.0-8.0) | 6.0 (4.0-8.0) | 0.05  (-0.36-0.45)^e^ |  | 6.0 (4.0-8.0) | 6.0 (4.0-7.0) | 0.11  (-0.27-0.49)^e^ |  |
|  | Min-Max | 2.0-10.0 | 2.0-10.0 | 2.0-10.0 | 2.0-10.0 |  |  | 2.0-10.0 | 2.0-10.0 |  |  | 2.0-10.0 | 2.0-10.0 |  |  | 2.0-10.0 | 2.0-10.0 |  |  |
| Future (3-15) | n | 71 | 72 | 59 | 53 |  |  | 55 | 47 |  |  | 51 | 46 |  |  | 58 | 50 |  |  |
|  | Mean (s.d.) | 9.86 (2.79) | 9.64 (2.53) | 9.97 (2.84) | 10.47 (2.60) | 1.30 (0.68-2.49)^a^ | 0.4297 | 9.85 (2.81) | 10.23 (2.66) | 1.32 (0.67-2.61)^a^ | 0.4250 | 10.22 (2.53) | 10.91 (2.51) | 1.71 (0.85-3.48)^a^ | 0.1375 | 9.69 (3.04) | 10.90 (2.34) | 2.00 (1.03-3.94)^a^ | **0.0422** |
|  | Median (Q1-Q3) | 10.0 (8.0-12.0) | 10.0 (8.0-11.0) | 11.0 (8.0-12.0) | 10.0 (9.0-13.0) | -0.19  (-0.56-0.19)^e^ |  | 10.0 (8.0-12.0) | 10.0 (9.0-12.0) | -0.14  (-0.53-0.26)^e^ |  | 10.0 (9.0-12.0) | 11.5 (9.0-13.0) | -0.28  (-0.68-0.13)^e^ |  | 10.0 (8.0-12.0) | 11.0 (10.0-12.0) | -0.44  (-0.83-  -0.05)^e^ |  |
|  | Min-Max | 3.0-15.0 | 3.0-15.0 | 4.0-15.0 | 5.0-15.0 |  |  | 4.0-15.0 | 4.0-15.0 |  |  | 4.0-15.0 | 4.0-15.0 |  |  | 3.0-15.0 | 6.0-15.0 |  |  |

|  | **Statistics** | **Control** | **Inter-vention** | **Control** | **Inter-vention** | **Treat-ment effect estimate (95% CI)** | **Sig.** | **Control** | **Inter-vention** | **Treat-ment effect estimate (95% CI)** | **Sig.** | **Control** | **Inter-vention** | **Treat-ment effect estimate (95% CI)** | **Sig.** | **Control** | **Inter-vention** | **Treat-ment effect estimate (95% CI)** | **Sig.** |
| --- | --- | --- | --- | --- | --- | --- | --- | --- | --- | --- | --- | --- | --- | --- | --- | --- | --- | --- | --- |
|  |  | *Baseline* | | *Week 1* | | | | *1 Month/ Week 5* | | | | *3 Month* | | | | *6 Month* | | | |
| Future Self-Questionnaire (FSQ) short version | | | | | | | | | | | | | | | | | | | |
| Before traumatic event | | | | | | | | | | | | | | | | | | | |
| Vividness (1-10) | n |  |  |  |  |  |  |  |  |  |  | 50 | 39 |  |  |  |  |  |  |
|  | Mean (s.d.) |  |  |  |  |  |  |  |  |  |  | 7.26 (2.52) | 7.54 (2.10) | 1.11 (0.53-2.32)^a^ | 0.7810 |  |  |  |  |
|  | Median (Q1-Q3) |  |  |  |  |  |  |  |  |  |  | 8.0 (6.0-10.0) | 8.0 (6.0-10.0) | -0.12  (-0.54-0.31)^e^ |  |  |  |  |  |
|  | Min-Max |  |  |  |  |  |  |  |  |  |  | 1.0-10.0 | 3.0-10.0 |  |  |  |  |  |  |
| Positivity (1-10) | n |  |  |  |  |  |  |  |  |  |  | 50 | 39 |  |  |  |  |  |  |
|  | Mean (s.d.) |  |  |  |  |  |  |  |  |  |  | 7.90 (2.23) | 8.05 (1.89) | 1.04 (0.50-2.19)^a^ | 0.9189 |  |  |  |  |
|  | Median (Q1-Q3) |  |  |  |  |  |  |  |  |  |  | 8.5 (7.0-10.0) | 9.0 (7.0-10.0) | -0.07  (-0.50-0.35)^e^ |  |  |  |  |  |
|  | Min-Max |  |  |  |  |  |  |  |  |  |  | 2.0-10.0 | 4.0-10.0 |  |  |  |  |  |  |
| Perspective | n |  |  |  |  |  |  |  |  |  |  |  |  | 0.33 (0.13-0.80)^b^ | **0.0162** |  |  |  |  |
| Through own eyes | n(%) |  |  |  |  |  |  |  |  |  |  | 23 (46.0) | 28 (71.8) |  |  |  |  |  |  |
| From outside | n(%) |  |  |  |  |  |  |  |  |  |  | 27 (54.0) | 11 (28.2) |  |  |  |  |  |  |

|  | **Statistics** | **Control** | **Inter-vention** | **Control** | **Inter-vention** | **Treat-ment effect estimate (95% CI)** | **Sig.** | **Control** | **Inter-vention** | **Treat-ment effect estimate (95% CI)** | **Sig.** | **Control** | **Inter-vention** | **Treat-ment effect estimate (95% CI)** | **Sig.** | **Control** | **Inter-vention** | **Treat-ment effect estimate (95% CI)** | **Sig.** |
| --- | --- | --- | --- | --- | --- | --- | --- | --- | --- | --- | --- | --- | --- | --- | --- | --- | --- | --- | --- |
|  |  | *Baseline* | | *Week 1* | | | | *1 Month/ Week 5* | | | | *3 Month* | | | | *6 Month* | | | |
| After traumatic event | | | | | | | | | | | | | | | | | | | |
| Vividness (1-10) | n |  |  |  |  |  |  |  |  |  |  | 50 | 39 |  |  |  |  |  |  |
|  | Mean (s.d.) |  |  |  |  |  |  |  |  |  |  | 7.02 (2.75) | 7.15 (2.71) | 1.06 (0.51-2.22)^a^ | 0.8697 |  |  |  |  |
|  | Median (Q1-Q3) |  |  |  |  |  |  |  |  |  |  | 8.0 (5.0-9.0) | 8.0 (6.0-9.0) | -0.05  (-0.47-0.38)^e^ |  |  |  |  |  |
|  | Min-Max |  |  |  |  |  |  |  |  |  |  | 1.0-10.0 | 1.0-10.0 |  |  |  |  |  |  |
| Positivity (1-10) | n |  |  |  |  |  |  |  |  |  |  | 50 | 39 |  |  |  |  |  |  |
|  | Mean (s.d.) |  |  |  |  |  |  |  |  |  |  | 6.56 (2.96) | 7.56 (2.54) | 1.82 (0.87-3.87)^a^ | 0.1139 |  |  |  |  |
|  | Median (Q1-Q3) |  |  |  |  |  |  |  |  |  |  | 7.0 (5.0-9.0) | 8.0 (6.0-10.0) | -0.36  (-0.79-0.07)^e^ |  |  |  |  |  |
|  | Min-Max |  |  |  |  |  |  |  |  |  |  | 1.0-10.0 | 1.0-10.0 |  |  |  |  |  |  |
| Perspective | n |  |  |  |  |  |  |  |  |  |  |  |  | 0.58 (0.18-1.66)^b^ | 0.3188 |  |  |  |  |
| Through  own eyes | n(%) |  |  |  |  |  |  |  |  |  |  | 38 (76.0) | 33 (84.6) |  |  |  |  |  |  |
| From outside | n(%) |  |  |  |  |  |  |  |  |  |  | 12 (24.0) | 6 (15.4) |  |  |  |  |  |  |
| Change of mental image content^8^ |  |  |  |  |  |  |  |  |  |  |  |  |  |  | 0.0684^d^ |  |  |  |  |
| No change in image | n(%) |  |  |  |  |  |  |  |  |  |  | 10 (20.4) | 8 (21.1) |  |  |  |  |  |  |
| Different way forward | n(%) |  |  |  |  |  |  |  |  |  |  | 24 (49.0) | 26 (68.4) |  |  |  |  |  |  |
| Weaker/less positive image | n(%) |  |  |  |  |  |  |  |  |  |  | 15 (30.6) | 4 (10.5) |  |  |  |  |  |  |

*Notes.* Secondary- and other pre-specified outcome measures (excluding Number of intrusive memories (see Table S7), adverse events (see Table S3 and 4), coping (see Table S11), expectancy rating (see Table 1), subjective units of distress (see Table S5), Feedback questionnaire (see Table S18), including baseline questionnaire at pre-randomization, diary assessments (Week 0, Week 1, Week 5) and follow-up questionnaires (1 week follow-up, 1 month follow-up, 3 month follow-up, 6 month follow-up). All applicable statistical tests are 2-sided and were performed using a 5% significance level. Tests of statistical significance was not undertaken for baseline characteristics.

^a^ Odds Ratio (OR) estimated using proportional odds logistic regression for ordinal outcomes; ^b^ Odds Ratio (OR) estimated using binomial logistic regression; ^c^ Incidence Rate Ratio (IRR) estimated using Quasi-Poisson regression with overdispersion parameter; ^d^ Chi-squared test; ^e^ Cohen’s *d*; ^0^ Higher scores are indicative for lower stress; ^1^ Higher scores indicated less difficulties; ² higher ratings indicated better sleep;^6^ higher ratings indicating less concentration and memory difficulties ^5^ “social support after traumatic event” and Self-rated impact of IMs on daily functioning in other areas” was coded on an erroneous scale (1-10 instead of 0-10); ^6^ Note, that at baseline the item was “whether the work situation changed related to COVID-19 pandemic.” ^7^ Only asked if item before that has been answered with yes. Free-text item that was coded by 3 nurse collaborators, no inter-rater reliability is available; ^8^ Categories of “change of mental image content” was rated by two-independent coders based on participants’ descriptions. Cohen’s kappa indicated substantial agreement (κ = .62).

*IM* intrusive memory.

Table S3. Number of Adverse Events by Treatment arm

|  | **Control N=71** | **Intervention N=73** | **Total N=144** | **p-value*** |
| --- | --- | --- | --- | --- |
| Number of subjects reporting any AE | 38 (53.5%) | 36 (49.3%) | 74 (51.4%) | 0.6136 |
| Number of AEs reported | 119 | 64 | 183 | 0.0052 |
| Number of subjects reporting any SAE | 0 (0.0%) | 0 (0.0%) | 0 (0.0%) |  |
| Number of SAEs | 0 | 0 | 0 |  |
| Number of AE leading to withdrawal | 0 (0.0%) | 1 (1.4%) | 1 (0.7%) |  |
| Number of subjects reporting any AE definitely related to intervention | 0 (0.0%) | 0 (0.0%) | 0 (0.0%) |  |
| Number of definitely related AEs | 0 | 0 | 0 |  |
| Number of subjects reporting any AE probably related to intervention | 0 (0.0%) | 0 (0.0%) | 0 (0.0%) |  |
| Number of probably related AEs | 0 | 0 | 0 |  |
| Number of subjects reporting any AE possibly related to intervention | 1 (1.4%) | 0 (0.0%) | 1 (0.7%) |  |
| Number of possibly related AEs | 1 | 0 | 1 |  |
| Number of subjects reporting any AE unlikely related to intervention | 2 (2.8%) | 2 (2.7%) | 4 (2.8%) |  |
| Number of unlikely related AEs | 2 | 2 | 4 |  |
| Number of subjects reporting any AE not related to intervention | 37 (52.1%) | 35 (47.9%) | 72 (50.0%) |  |
| Number of not related AEs | 116 | 62 | 178 |  |
| Number of subjects reporting any AE with maximal intensity = Severe | 1 (1.4%) | 1 (1.4%) | 2 (1.4%) |  |
| Number of AEs with maximal intensity = Severe | 2 | 1 | 3 |  |
| Number of subjects reporting any AE with maximal intensity = Moderate | 20 (28.2%) | 7 (9.6%) | 27 (18.8%) |  |
| Number of AEs with maximal intensity = Moderate | 42 | 7 | 49 |  |
| Number of subjects reporting any AE with maximal intensity =Mild | 30 (42.3%) | 31 (42.5%) | 61 (42.4%) |  |
| Number of AEs with maximal intensity = Mild | 75 | 56 | 131 |  |

*Note.* All adverse events reported across all time-points. Adverse events were reported by the participants during the follow-up assessments (1-week, 1 month, 3 months, and 6 months) by providing a free text response to the question: “Have you had any health problems since the last contact?”. Participants could also spontaneously report AEs to study personnel throughout the trial. *AE*, Adverse events; *SAE*, Serious adverse event. *Statistical test between arms was performed only for the total number of AE and the number of patients having at least one AE. Chi-squared test was used to compare treatment arms in number (%) of subjects reporting AE and Quasi-Poisson regression was used to compare number of adverse events between arms.

Table S4. Types of Adverse Events by Treatment arm at each time point.

|  | AEs before researcher-guided session, n= | | AEs in researcher-guided session,  n= | | AEs up to and including One week, n= | | AEs up to and including One month, n= | | AEs up to and including 3 month, n= | | AEs up to and including 6 month, n= | |
| --- | --- | --- | --- | --- | --- | --- | --- | --- | --- | --- | --- | --- |
|  | Control n=3 | Intervention n=1 | Control n=0 | Intervention n=2 | Control  n=24 | Intervention  n=10 | Control  n=35 | Intervention  n=10 | Control  n=33 | Intervention  n=14 | Control  n=24 | Intervention n=27 |
| *Mental Health AEs (total)* | 0 | 0 | 0 | 0 | 7 | 3 | 23 | 6 | 16 | 5 | 8 | 9 |
| Burnout/tiredness | 0 | 0 | 0 | 0 | 4 | 1 | 5 | 0 | 3 | 1 | 2 | 0 |
| Depression | 0 | 0 | 0 | 0 | 1 | 1 | 3 | 1 | 3 | 0 | 2 | 2 |
| Panic | 0 | 0 | 0 | 0 | 1 | 0 | 1 | 0 | 2 | 0 | 1 | 0 |
| Anxiety/worry | 0 | 0 | 0 | 0 | 1 | 0 | 3 | 2 | 3 | 1 | 0 | 2 |
| Stress | 0 | 0 | 0 | 0 | 0 | 1 | 2 | 1 | 2 | 1 | 0 | 1 |
| PTSD symptoms | 0 | 0 | 0 | 0 | 0 | 0 | 1 | 0 | 1 | 0 | 2 | 0 |
| Eating disorder/reduced  appetite | 0 | 0 | 0 | 0 | 0 | 0 | 0 | 0 | 0 | 1 | 0 | 1 |
| Problems with sleep | 0 | 0 | 0 | 0 | 0 | 0 | 4 | 2 | 2 | 1 | 1 | 2 |
| Cognitive difficulties | 0 | 0 | 0 | 0 | 0 | 0 | 2 | 0 | 0 | 0 | 0 | 0 |
| Unspecified mental  health problems | 0 | 0 | 0 | 0 | 0 | 0 | 2 | 0 | 0 | 0 | 0 | 1 |
| *Physical Health AEs (total*) | 3 | 1 | 0 | 2 | 17 | 7 | 12 | 4 | 16 | 9 | 16 | 18 |
| Stomach problems | 1 | 0 | 0 | 0 | 3 | 1 | 3 | 1 | 0 | 0 | 1 | 2 |
| Headache/migraine | 0 | 1 | 0 | 0 | 1 | 2 | 1 | 0 | 1 | 2 | 0 | 1 |
| Throat problems | 0 | 0 | 0 | 0 | 1 | 0 | 0 | 0 | 0 | 0 | 0 | 2 |
| Sinus infection | 0 | 0 | 0 | 0 | 0 | 1 | 0 | 0 | 0 | 0 | 0 | 0 |
| Heart problems | 0 | 0 | 0 | 0 | 0 | 0 | 0 | 1 | 1 | 0 | 2 | 0 |
| High blood pressure | 0 | 0 | 0 | 0 | 0 | 0 | 0 | 0 | 0 | 0 | 0 | 1 |
| Back/neck problems | 0 | 0 | 0 | 1 | 1 | 1 | 0 | 0 | 2 | 0 | 0 | 1 |
| Common cold | 1 | 0 | 0 | 0 | 3 | 0 | 0 | 1 | 1 | 1 | 3 | 2 |
| Infection | 0 | 0 | 0 | 0 | 0 | 0 | 0 | 0 | 0 | 0 | 2 | 0 |
| Dental problem | 0 | 0 | 0 | 0 | 0 | 0 | 1 | 0 | 1 | 0 | 1 | 0 |
| Shoulder problem | 0 | 0 | 0 | 0 | 0 | 0 | 0 | 0 | 1 | 0 | 1 | 0 |
| Broken arm | 0 | 0 | 0 | 0 | 0 | 0 | 0 | 0 | 0 | 0 | 0 | 1 |
| Gynaecological  problems | 0 | 0 | 0 | 0 | 0 | 0 | 0 | 0 | 1 | 0 | 0 | 2 |
| Sprainedfoot/feet pain | 0 | 0 | 0 | 0 | 1 | 0 | 1 | 0 | 0 | 0 | 0 | 0 |
| Pain in joints/muscles | 0 | 0 | 0 | 0 | 2 | 0 | 1 | 0 | 1 | 0 | 2 | 2 |
| Torn meniscus | 0 | 0 | 0 | 0 | 0 | 0 | 0 | 0 | 1 | 0 | 1 | 0 |
| Surgery | 0 | 0 | 0 | 0 | 1 | 0 | 1 | 0 | 1 | 0 | 0 | 0 |
| Basal-cell carcinoma | 0 | 0 | 0 | 0 | 0 | 0 | 1 | 0 | 0 | 0 | 0 | 0 |
| Gynecological surgery | 1 | 0 | 0 | 0 | 0 | 0 | 0 | 0 | 0 | 0 | 0 | 0 |
| Herniorrhaphy | 0 | 0 | 0 | 0 | 0 | 0 | 0 | 0 | 0 | 0 | 1 | 0 |
| Thyroidectomy | 0 | 0 | 0 | 0 | 1 | 0 | 1 | 0 | 1 | 0 | 0 | 0 |
| Covid-19 | 0 | 0 | 0 | 0 | 1 | 0 | 0 | 0 | 2 | 1 | 1 | 3 |
| Breast cancer | 0 | 0 | 0 | 0 | 1 | 0 | 0 | 0 | 1 | 0 | 0 | 0 |
| Cancer treatment | 0 | 0 | 0 | 0 | 0 | 0 | 1 | 1 | 0 | 1 | 0 | 0 |
| Vertigo/fainting | 0 | 0 | 0 | 1 | 0 | 1 | 1 | 0 | 0 | 0 | 0 | 0 |
| Asthma | 0 | 0 | 0 | 0 | 0 | 0 | 0 | 0 | 1 | 1 | 0 | 0 |
| Cell dysplasia | 0 | 0 | 0 | 0 | 1 | 0 | 0 | 0 | 0 | 0 | 0 | 0 |
| Overweight | 0 | 0 | 0 | 0 | 0 | 0 | 0 | 0 | 0 | 1 | 0 | 0 |
| Cystic kidney disease | 0 | 0 | 0 | 0 | 0 | 0 | 0 | 0 | 0 | 1 | 0 | 0 |
| Gall stone | 0 | 0 | 0 | 0 | 0 | 0 | 0 | 0 | 0 | 0 | 1 | 0 |
| Unspecified physical problems | 0 | 0 | 0 | 0 | 0 | 1 | 0 | 0 | 0 | 1 | 0 | 1 |
| *Sick leave without specified reason ^1^* | 0 | 0 | 0 | 0 | 0 | 0 | 0 | 0 | 1 | 0 | 0 | 0 |

*Note*. Total number of AEs reported by category. AEs types are grouped by Mental Health, Physical Health and Sick leave without specified reason. AE type (mental/physical health) was rated by two-independent coders based on AE descriptions. Cohen’s kappa indicated high agreement (κ = .989). *AE*, Adverse events; *PTSD*, Post traumatic stress disorder.
^1^Participants that reported Sick leave with no further information of any mental or physical health symptoms.

Table S5. Subjective Units of Distress (SUDS)

|  | **Statistics** | **Control n=71** | **Intervention n=73** | **Total n=144** | **Mean Ratio** | **Treatment effect estimate (95% CI)** | **p-value** |
| --- | --- | --- | --- | --- | --- | --- | --- |
| Subjective Units of Distress 1  (0-10) | n | 69 | 73 | 142 |  |  |  |
|  | Mean (s.d.) | 1.99 (2.32) | 1.44 (1.99) | 1.70 (2.16) | 0.72 | 0.69 (0.38-1.26)^a^ | 0.2298 |
|  | Median (Q1-Q3) | 1.0 (0.0-4.0) | 1.0 (0.0-2.0) | 1.0 (0.0-3.0) |  |  |  |
|  | Min-Max | 0.0-8.0 | 0.0-8.0 | 0.0-8.0 |  |  |  |
| Subjective Units of Distress 2  (0-10) | n | 69 | 71 | 140 |  |  |  |
|  | Mean (s.d.) | 1.90 (2.46) | 5.45 (2.36) | 3.70 (2.99) | 2.87 | 12.20 (6.14-25.22)^a^ | <0.0001 |
|  | Median (Q1-Q3) | 1.0 (0.0-3.0) | 5.0 (4.0-7.0) | 4.0 (0.5-6.0) |  |  |  |
|  | Min-Max | 0.0-9.0 | 0.0-10.0 | 0.0-10.0 |  |  |  |
| Subjective Units of Distress 3  (0-10) | n | 68 | 70 | 138 |  |  |  |
|  | Mean (s.d.) | 1.81 (2.31) | 1.71 (2.27) | 1.76 (2.28) | 0.95 | 0.99 (0.54-1.81)^a^ | 0.9732 |
|  | Median (Q1-Q3) | 1.0 (0.0-3.0) | 1.0 (0.0-3.0) | 1.0 (0.0-3.0) |  |  |  |
|  | Min-Max | 0.0-9.0 | 0.0-10.0 | 0.0-10.0 |  |  |  |

^a^ Odds Ratio (OR) – Estimated using proportional odds logistic regression.

Table S6. Diagnostics for the multiple imputation

**Mean number of intrusive memories by day at week 0**

|  | Observed | | | | Imputed | | | |
| --- | --- | --- | --- | --- | --- | --- | --- | --- |
| Variable | Mean | Std Dev | Minimum | Maximum | Mean | Std Dev | Minimum | Maximum |
| w0_ims_d1 w0_ims_d2 w0_ims_d3 w0_ims_d4 w0_ims_d5 w0_ims_d6 w0_ims_d7 | 3.14 3.76 3.32 2.99 2.68 2.60 2.60 | 2.58 4.58 3.51 4.01 3.36 2.98 2.94 | 0.00 0.00 0.00 0.00 0.00 0.00 0.00 | 12.00 39.00 16.00 27.00 23.00 14.00 17.00 | 2.59 4.79 2.93 2.95 2.05 2.75 2.50 | 1.83 4.20 3.34 3.02 2.07 2.69 2.96 | 0.00 0.00 0.00 0.00 0.00 0.00 0.00 | 10.00 39.00 16.00 14.00 9.00 13.00 12.00 |

**Mean number of intrusive memories by day at week 1**

|  | Observed | | | | Imputed | | | |
| --- | --- | --- | --- | --- | --- | --- | --- | --- |
| Variable | Mean | Std Dev | Minimum | Maximum | Mean | Std Dev | Minimum | Maximum |
| w1_ims_d1 w1_ims_d2 w1_ims_d3 w1_ims_d4 w1_ims_d5 w1_ims_d6 w1_ims_d7 | 1.63 2.00 2.02 1.84 2.06 1.68 1.52 | 2.33 2.47 2.88 3.06 5.50 2.28 2.37 | 0.00 0.00 0.00 0.00 0.00 0.00 0.00 | 17.00 9.00 15.00 25.00 57.00 10.00 13.00 | 1.69 2.07 1.83 1.48 1.53 1.80 1.69 | 1.48 2.50 2.73 2.02 2.44 2.25 2.56 | 0.00 0.00 0.00 0.00 0.00 0.00 0.00 | 8.00 9.00 15.00 12.00 13.00 10.00 13.00 |

**Mean number of intrusive memories by day at week 5**

|  | Observed | | | | Imputed | | | |
| --- | --- | --- | --- | --- | --- | --- | --- | --- |
| Variable | Mean | Std Dev | Minimum | Maximum | Mean | Std Dev | Minimum | Maximum |
| w5_ims_d1 w5_ims_d2 w5_ims_d3 w5_ims_d4 w5_ims_d5 w5_ims_d6 w5_ims_d7 | 0.70 1.22 1.03 1.20 1.14 1.37 1.22 | 1.31 2.22 1.97 2.92 2.47 2.99 2.56 | 0.00 0.00 0.00 0.00 0.00 0.00 0.00 | 6.00 14.00 12.00 19.00 15.00 22.00 13.00 | 0.77 1.25 0.72 0.89 1.12 1.24 0.90 | 1.34 2.25 1.42 1.98 2.31 2.39 2.26 | 0.00 0.00 0.00 0.00 0.00 0.00 0.00 | 6.00 14.00 9.00 14.00 13.00 10.00 13.00 |

**Mean of the total number of intrusive memories at Week 0 (before and after imputation)**

| Variable | Mean | Std Dev | Minimum | Maximum |
| --- | --- | --- | --- | --- |
| IM at Week 0 imputed IM at Week 0 | 21.05 21.08 | 18.93 19.22 | 0.00 0.00 | 137.00 137.00 |

**Mean of the total number of intrusive memories at Week 1 (before and after imputation)**

| Variable | Mean | Std Dev | Minimum | Maximum |
| --- | --- | --- | --- | --- |
| IM at Week 1 imputed IM at Week 1 | 12.71 12.78 | 16.87 17.58 | 0.00 0.00 | 140.00 140.00 |

**Mean of the total number of intrusive memories at Week 5 (before and after imputation)**

| Variable | Mean | Std Dev | Minimum | Maximum |
| --- | --- | --- | --- | --- |
| IM at Week 5 imputed IM at Week 5 | 7.67 7.85 | 13.38 14.34 | 0.00 0.00 | 90.00 90.00 |

Table S7a. Number of intrusive memories at Week 0 (baseline), Week 1, and Week 5 (primary outcome) for complete diary data.

| **Time point** | **Statistic** | **Control** | **Intervention** | **Total** | **Mean Ratio** | **Treatment effect estimate (95% CI)** | **p-value** |
| --- | --- | --- | --- | --- | --- | --- | --- |
| Week 0 (Day 7 to 1)^1^ | n | 69 | 67 | 136 |  |  |  |
|  | Mean (s.d.) | 19.06 (16.55) | 23.16 (21.69) | 21.08 (19.29) | 1.22 |  |  |
|  | Median (Q1-Q3) | 14.0 (7.0-25.0) | 18.0 (8.0-29.0) | 15.0 (7.5-28.5) |  |  |  |
|  | Min-Max | 0.0-73.0 | 2.0-137.0 | 0.0-137.0 |  |  |  |
| Week 1 | n | 65 | 64 | 129 |  |  |  |
|  | Mean (s.d.) | 14.85 (14.28) | 10.69 (20.41) | 12.78 (17.64) | 0.72 | 0.51 (0.38-0.68)^a^ | <0.0001 |
|  | Median (Q1-Q3) | 11.0 (3.0-21.0) | 4.5 (2.0-10.5) | 7.0 (3.0-15.0) |  | 0.24 (-0.11-0.59)^b^ |  |
|  | Min-Max | 0.0-54.0 | 0.0-140.0 | 0.0-140.0 |  |  |  |
| Week 5^2^ | n | 58 | 60 | 118 |  |  |  |
|  | Mean (s.d.) | 12.41 (17.53) | 3.43 (8.60) | 7.85 (14.40) | 0.28 |  |  |
|  | Median (Q1-Q3) | 5.0 (1.0-17.0) | 0.5 (0.0-3.0) | 2.0 (0.0-8.0) |  | 0.65 (0.28-1.03)^b^ |  |
|  | Min-Max | 0.0-90.0 | 0.0-47.0 | 0.0-90.0 |  |  |  |

*Note.* These data are based on the ITT sample including the *complete* diary data for participants that reported numbers for every day in the week or the total for that week. The baseline number of intrusive memories (during day-7 to -1) has been included as a covariate. ^a^ Incidence Rate Ratio; ^b^ Cohen’s *d.* ^1^ Tests of statistical significance were not undertaken for Week 0. ² Analysed separately (see **Table S15).**

Table S7b. Number of intrusive memories at Week 0 (baseline), Week 1, and Week 5 (primary outcome) including incomplete diary data.

| **Time point** | **Statistic** | **Control** | **Intervention** | **Total** | **Mean Ratio** | **Treatment effect estimate (95% CI)^1^** | **p-value** |
| --- | --- | --- | --- | --- | --- | --- | --- |
| Week 0 (Day -7 to -1) | n | 71 | 73 | 144 |  |  |  |
|  | Mean (s.d.) | 19.48 (16.50) | 21.79 (21.32) | 20.65 (19.06) | 1.12 |  |  |
|  | Median (Q1-Q3) | 14.0 (7.0-28.0) | 16.0 (6.0-29.0) | 15.0 (6.25-28.75) |  |  |  |
|  | Min-Max | 0.0-73.0 | 2.0-137.0 | 0.0-137.0 |  |  |  |
| Week 1 | n | 67 | 69 | 136 |  |  |  |
|  | Mean (s.d.) | 14.67 (14.19) | 10.26 (19.71) | 12.43 (17.29) | 0.70 |  |  |
|  | Median (Q1-Q3) | 11 (3.0-21.0) | 4.0 (2.0-10.0) | 7.0 (3.0-15.0) |  |  |  |
|  | Min-Max | 0.0-54.0 | 0.0-140.0 | 0.0.-140.0 |  |  |  |
| Week 5 | n | 66 | 64 | 130 |  |  |  |
|  | Mean (s.d.) | 11.26 (16.79) | 3.27 (8.35) | 7.32 (13.86) | 0.29 |  |  |
|  | Median (Q1-Q3) | 4.0 (1.0-15.0) | 0.5 (0.0-2.75) | 2.0 (0.0-6.5) |  |  |  |
|  | Min-Max | 0.0-90.0 | 0.0-47.0 | 0.0-90.0 |  |  |  |

*Note.* These data are based on the ITT sample including *incomplete* diary data. (see also **Table 2).** ^1^ Tests of statistical significance were not undertaken.

Figure S1. Time course of the number of intrusive memories day-by-day.

Frequency scatter graphs showing the time course of the number of intrusive memories recorded in a diary from day 1 (day of completing the intervention/control procedure) to day 7 for participants who reported intrusive memories seven days per week at least once per day in the intervention arm and control arm on a daily basis (A: week 0: n control=69; n intervention=67; B: week 1: n control=63; n intervention=62; C: week 5: n control=54; n intervention=57). The size of the circles represents the number of participants who reported the indicated number of intrusive memories on that particular day, scaled separately for each arm; the black line indicates mean per day; red = intervention arm; black = control arm.

**
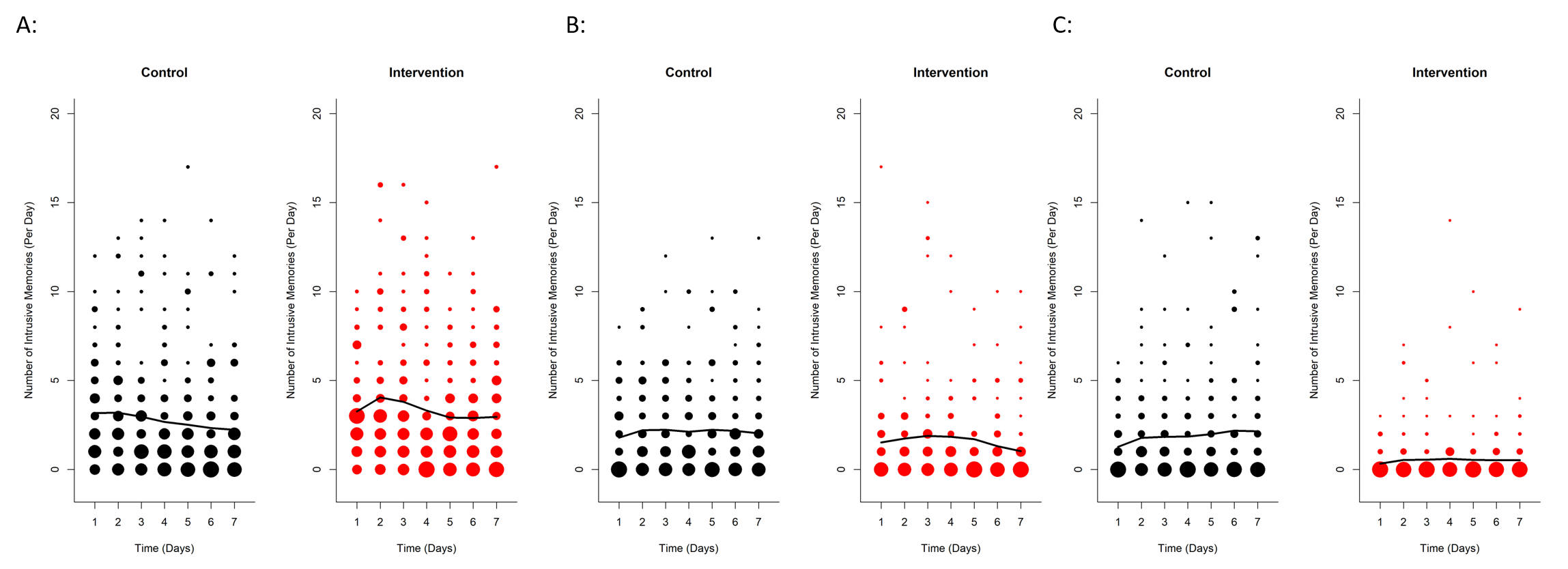
**

Table S8. Breakdown of the category ’other’ in the Type of trauma(s) leading to intrusive memories questionnaire in Table 1 of the main paper.

|  | **Statistics** | **Control n=71** | **Intervention  n=73** | **Total  n=144** |
| --- | --- | --- | --- | --- |
| **Traumatic event in relation to clinical work during COVID-19 pandemic** | n |  |  | 144 |
| A traumatic or tragic death of a patient | n (%) | 57  (39.6%) | 56 (38.9%) | 113 (78.5%) |
| A severe or unsuccessful resuscitation | n (%) | 15  (10.4%) | 18 (12.5%) | 33 (22.9%) |
| Witnessing events surrounding colleague who has fallen ill or died of COVID-19 | n (%) | 11  (7.6%) | 15 (10.4%) | 26 (18.1%) |
| Situation where the care of a patient failed or did not go as planned | n (%) | 46  (31.9%) | 51 (35.4%) | 97 (67.4%) |
| Threats or violence against healthcare professionals | n (%) | 12  (8.3%) | 14 (9.7%) | 26 (18.1%) |
| Event involving sudden increased risk of COVID-19 infection | n (%) | 36  (25%) | 32 (22.2%) | 68 (47.2%) |
| A traumatic or tragic event where a patient reminded you of yourself, a family member or friend | n (%) | 26  (18.1%) | 33 (22.9%) | 59 (41.0%) |
| Event involving extremely distressed/grieving relatives of patients | n (%) | 41  (28.5%) | 46 (31.9%) | 87 (60.4%) |
| Being faced with suicide / suicide attempt | n (%) | 5  (3.5%) | 7  (4.7%) | 12 (8.3%) |
| Other (yes/no) | n (%) | 12  (8.3%) | 16 (11.1%) | 28 (19.4%) |
| **Content of other *** |  |  |  |  |
| Critical clinical situations at work with overwhelming workload. Inability to provide aid as needed | n (%) | 5  (3.5%) | 9  (6.3%) | 14 (9.7%) |
| Witnessing patients mentally and/or physically suffer | n (%) | 8  (5.6%) | 6  (5.6%) | 14 (9.7%) |
| Accident at work | n (%) | 1  (0.7%) | 1  (0.7%) | 2  (1.4%) |
| Unclear work responsibilities and management | n (%) | 3  (2.1%) | 1  (0.7%) | 4  (2.8%) |
| Earlier traumatic event before COVID-19 | n (%) | 0 | 1  (0.7%) | 1  (0.7%) |
| Stressors outside workplace possibly related to COVID-19 | n (%) | 2  (1.4%) | 1  (0.7%) | 3  (2.1%) |

*Note.* *Categories of “other” was rated by two-independent coders based on participants’ descriptions. Cohen’s kappa indicated substantial agreement (κ = .80).

Table S9. Number of prior psychological traumas (LEC-5) per category (baseline)

|  | **Statistics** | **Control  n=71** | **Intervention  n=73** | **Total n=144** |
| --- | --- | --- | --- | --- |
| **Number of prior psychological trauma per category (LEC-5)** | n | 71 | 73 | 144 |
| Natural disaster (e.g. flood, hurricane, tornado, earthquake) | n (%) | 5 (3.5%) | 11 (7.6%) | 16 (11.1%) |
| Fire or explosion | n (%) | 22 (15.3%) | 24 (16.7%) | 46 (31.9%) |
| Transportation accident (e.g. car accident, boat accident, train wreck, plane crash) | n (%) | 29 (20.1%) | 28 (19.4%) | 57 (39.6%) |
| Serious accident at work, home or during recreational activity | n (%) | 29 (20.1%) | 28 (19.4%) | 57 (39.6%) |
| Exposure to toxic substance (e.g. dangerous chemicals, radiation) | n (%) | 11 (7.6%) | 7 (4.9%) | 18 (12.5%) |
| Physical assault (e.g. being attacked, hit, slapped, kicked, beaten up) | n (%) | 33 (22.9%) | 38 (26.4%) | 71 (49.3%) |
| Assault with a weapon (e.g. being shot, stabbed, threatened with a knife, gun, bomb) | n (%) | 13 (9.0%) | 19 (13.2%) | 32 (22.2%) |
| Sexual assault (rape, attempted rape, made to perform any type of sexual act through force or threat of harm) | n (%) | 15 (10.4%) | 17 (11.8%) | 32 (22.2%) |
| Other unwanted or uncomfortable sexual experience | n (%) | 26 (18.1%) | 23 (16%) | 49 (34.0%) |
| Combat or exposure to a war zone (in the military or as a civilian) | n (%) | 4 (2.8%) | 8 (5.6%) | 12 (8.3%) |
| Captivity (e.g. being kidnapped, abducted, held hostage, prisoner of war) | n (%) | 3 (2.1%) | 7 (4.9%) | 10 (6.9%) |
| Life-threatening illness or injury | n (%) | 39 (27.1%) | 47 (32.6%) | 86 (59.7%) |
| Severe human suffering | n (%) | 49 (34.0%) | 60 (41.7%) | 109 (75.7%) |
| Sudden violent death (e.g. homicide, suicide) | n (%) | 29 (20.1%) | 31 (21.5%) | 60 (41.7%) |
| Sudden accidental death | n (%) | 29 (20.1%) | 31 (21.5%) | 60 (41.7%) |
| Serious injury, harm or death respondent caused to someone else | n (%) | 3 (2.1%) | 4 (2.8%) | 7 (4.9%) |
| Any other very stressful event or experience | n (%) | 40 (27.8%) | 45 (31.3%) | 85 (59.0%) |

*Note. LEC-5* Life Events Checklist for DSM-5. Percentages here are calculated from the total (144).

Table S10. Coping categories based on free text responses assessed at baseline.

|  | **Statistics** | **Control  n=71** | **Intervention  n=73** | **Total n=144** |
| --- | --- | --- | --- | --- |
| **Coping** |  |  |  |  |
| Situations or factors during the COVID-19 pandemic that made it difficult to cope | n | 71 | 73 | 144 |
| Lack of knowledge about Covid-19, e.g. transmission and  treatment |  | 17  (11.8%) | 26  (18.1%) | 43 (29.9%) |
| Additional workload and/or stress due to patient flow, lack of staff, resources and/or time |  | 23  (16.0%) | 21  (14.6%) | 44 (30.6%) |
| Inability to provide aid as needed |  | 21  (14.6%) | 24  (16.7%) | 45 (31.3%) |
| Unclear directives and lack of support by management |  | 11  (7.6%) | 11  (7.6%) | 22 (15.3%) |
| Witnessing deaths and mental and/or physical suffering of  patients and their relatives |  | 27  (18.8%) | 26  (18.1%) | 53 (36.8%) |
| Stigma or lack of support from general public and family  members |  | 4  (2.8%) | 3  (2.1%) | 7 (4.9%) |
| Concerns for family members and/or colleagues |  | 1  (0.7%) | 6  (4.2%) | 7 (4.9%) |
| Concerns for own health and safety |  | 5  (3.5%) | 5  (3.5%) | 10 (6.9%) |
| Broken collaboration, e.g. bad communication between  colleagues |  | 3  (2.1%) | 4  (2.8%) | 7 (4.9%) |
| Other |  | 7  (4.9%) | 13  (9.0%) | 20 (13.9) |
| Factors during the COVID-19 pandemic that made it easier to cope | n | 71 | 73 | 144 |
| Support from loved ones |  | 42  (29.2%) | 36  (25.0%) | 78 (54.2%) |
| Support from colleagues, i.e. collaboration |  | 24  (16.7%) | 25  (17.4%) | 49 (34.0%) |
| Share mutual work experiences |  | 14  (9.7%) | 16  (11.1%) | 30 (20.8%) |
| Earlier life or work experience |  | 15  (10.4%) | 11  (7.6%) | 26 (18.1%) |
| Education and information |  | 13  (9.0%) | 6  (4.2%) | 19 (13.2%) |
| Infection control measures, i.e. vaccines, restrictions |  | 5  (3.5%) | 12  (8.3%) | 17 (11.8%) |
| Activities apart from work, i.e. sport, yoga |  | 13  (9.0%) | 15  (10.4%) | 28 (19.4%) |
| Religion |  | 1  (0.7%) | 0 | 1 (0.7%) |
| Humor |  | 2  (1.4%) | 1  (0.7%) | 3 (2.1%) |
| Avoidance and/or distancing, i.e. working on autopilot |  | 2  (1.4%) | 4  (2.8%) | 6 (4.2%) |
| Seeking mental and physical health support |  | 2  (1.4%) | 0 | 2 (1.4%) |
| Secure home |  | 1  (0.7%) | 0 | 1 (0.7%) |
| Ethical and professional duty and/or pride in their role |  | 5  (3.5%) | 3  (2.1%) | 8 (5.6%) |
| Support from management, i.e., clear directives, crisis support |  | 8  (5.6%) | 5  (3.5%) | 13 (9.0%) |
| Sufficient resources at workplace, i.e. safety equipment, staffing |  | 7  (4.9%) | 1  (0.7%) | 8 (5.6%) |
| Other |  | 4  (2.8%) | 8  (5.6%) | 12 (8.3%) |
| No coping strategy applied |  | 11  (7.6%) | 11  (7.6%) | 22 (15.3%) |

*Note.* Categories of Coping was rated by two-independent coders based on participants’ descriptions. Cohen’s kappa indicated substantial agreement (κ_situations_ = .65 and κ_factors_ = .75).

Percentages here are calculated from the total (144).

Table S11. Sensitivity analysis Primary endpoint analysed with non-parametric Wilcoxon’s rank sum test.

| Outcome | Population | n | Observed Median (IQR) group A | Observed Median (IQR) group B | p-value* |
| --- | --- | --- | --- | --- | --- |
| Number of intrusive memories of traumatic event(s) at week 5 | ITT | 118 | 5 (1-17) | 1 (0-3) | <.0001 |
| Number of intrusive memories of traumatic event(s) at week 5 | PP | 115 | 6 (1-18) | 1 (0-3) | <.0001 |

*Non-parametric test to compare number of intrusive memories at week 5 between treatment groups

**Results are based on complete case (all number of intrusive memories per day reported at week 5) and only total number of IM at week 5 reported

Table S12. Primary outcome missing at random (MAR).

| Outcome^1^ | Population | n | Observed Median (IQR) group Control^1^ | Observed Median (IQR) group Intervention^1^ | Observed Mean (SD) group Control^1^ | Observed Mean (SD) group Intervention^1^ | Reference group | Estimated Incidence Rate Ratio (95% CI)² | p-value |
| --- | --- | --- | --- | --- | --- | --- | --- | --- | --- |
| Number of intrusive memories of traumatic event(s) at week 5 | ITT | 144 | 5 (1-17) | 1 (0-3) | 12.4 (17.5) | 3.4 (8.6) | Control | 0.30 (0.17-0.53) | <0.0001 |
| Number of intrusive memories of traumatic event(s) at week 5 | PP | 127 | 6 (1-18) | 1 (0-3) | 12.8 (17.7) | 3.5 (8.7) | Control | 0.29 (0.16-0.53) | <0.0001 |
| Number of intrusive memories of traumatic event(s) at week 1 | ITT | 144 | 11 (3-21) | 5 (2-11) | 14.8 (14.3) | 10.7 (20.4) | Control | 0.53 (0.41-0.70) | <0.0001 |
| Number of intrusive memories of traumatic event(s) at week 1 | PP | 127 | 11 (4-22) | 4 (2-10) | 15.2 (14.3) | 8.1 (11.0) | Control | 0.52 (0.40-0.67) | <0.0001 |

*Note.* ^1^ Based on complete data, ITT Control arm n=58, ITT Intervention arm n=60; PP Control arm n=56, PP Intervention arm n=59.

² Results of Quasi-Poisson regression model with overdispersion parameter based on imputed data. The baseline number of intrusive memories (at day-7 to -1) is included as a covariate.

Table S13. Sensitivity analysis primary outcome missing not at random (MNAR).

| Outcome | Study group | Mean observed primary outcome | Mean imputed primary outcome | Differences between intervention and control group | Shift constant | Estimated Incidence Rate Ratio (95% CI)^1^ | p-value |
| --- | --- | --- | --- | --- | --- | --- | --- |
| IM at Week 1 | Control | 14.85 | 14.55 | Intervention vs Control | 0 | 0.52 (0.39-0.70) | <0.0001 |
|  | Intervention | 10.69 | 9.17 |  | . |  | . |
| IM at Week 1 | Control | 14.85 | 14.55 | Intervention vs Control | 1 | 0.52 (0.39-0.70) | <0.0001 |
|  | Intervention | 10.69 | 8.82 |  | . |  | . |
| IM at Week 1 | Control | 14.85 | 14.55 | Intervention vs Control | 2 | 0.53 (0.40-0.71) | <0.0001 |
|  | Intervention | 10.69 | 9.99 |  | . |  | . |
| IM at Week 1 | Control | 14.85 | 14.55 | Intervention vs Control | 3 | 0.53 (0.40-0.70) | <0.0001 |
|  | Intervention | 10.69 | 9.54 |  | . |  | . |
| IM at Week 1 | Control | 14.85 | 14.55 | Intervention vs Control | 4 | 0.54 (0.40-0.73) | <0.0001 |
|  | Intervention | 10.69 | 11.23 |  | . |  | . |
| IM at Week 1 | Control | 14.85 | 14.55 | Intervention vs Control | 5 | 0.54 (0.40-0.73) | <0.0001 |
|  | Intervention | 10.69 | 11.47 |  | . |  | . |
| IM at Week 1 | Control | 14.85 | 14.55 | Intervention vs Control | 10 | 0.58 (0.43-0.78) | 0.0003 |
|  | Intervention | 10.69 | 15.54 |  | . |  | . |
| IM at Week 1 | Control | 14.85 | 14.55 | Intervention vs Control | 15 | 0.61 (0.45-0.82) | 0.0013 |
|  | Intervention | 10.69 | 19.2 |  | . |  | . |
| IM at Week 1 | Control | 14.85 | 14.55 | Intervention vs Control | 20 | 0.65 (0.47-0.89) | 0.0067 |
|  | Intervention | 10.69 | 23.67 |  | . |  | . |
| IM at Week 1 | Control | 14.85 | 14.55 | Intervention vs Control | 21 | 0.66 (0.48-0.91) | 0.0121 |
|  | Intervention | 10.69 | 25.67 |  | . |  | . |
| IM at Week 1 | Control | 14.85 | 14.55 | Intervention vs Control | 30 | 0.72 (0.52-1.00) | 0.0498 |
|  | Intervention | 10.69 | 31.82 |  | . |  | . |
| IM at Week 1 | Control | 14.85 | 14.55 | Intervention vs Control | 30 | 0.72 (0.52-1.00) | 0.0498 |
|  | Intervention | 10.69 | 31.82 |  | . |  | . |
| IM at Week 1 | Control | 14.85 | 14.55 | Intervention vs Control | 31 | 0.72 (0.52-1.00) | 0.0484 |
|  | Intervention | 10.69 | 32.35 |  | . |  | . |
| IM at Week 1 | Control | 14.85 | 14.55 | Intervention vs Control | 31.5 | 0.73 (0.53-1.01) | 0.0578 |
|  | Intervention | 10.69 | **33.3** |  | . |  | . |
| IM at Week 1 | Control | 14.85 | 14.55 | Intervention vs Control | 32 | 0.74 (0.53-1.03) | 0.0720 |
|  | Intervention | 10.69 | 34.36 |  | . |  | . |
| IM at Week 1 | Control | 14.85 | 14.55 | Intervention vs Control | 34 | 0.73 (0.52-1.01) | 0.0601 |
|  | Intervention | 10.69 | 33.09 |  | . |  | . |
| IM at Week 5 | Control | 12.41 | 5.75 | Intervention vs Control | 0 | 0.28 (0.15-0.56) | 0.0003 |
|  | Intervention | 3.43 | 8.17 |  | . |  | . |
| IM at Week 5 | Control | 12.41 | 5.75 | Intervention vs Control | 1 | 0.28 (0.15-0.55) | 0.0002 |
|  | Intervention | 3.43 | 7.99 |  | . |  | . |
| IM at Week 5 | Control | 12.41 | 5.75 | Intervention vs Control | 2 | 0.30 (0.16-0.57) | 0.0002 |
|  | Intervention | 3.43 | 8.86 |  | . |  | . |
| IM at Week 5 | Control | 12.41 | 5.75 | Intervention vs Control | 3 | 0.30 (0.16-0.58) | 0.0004 |
|  | Intervention | 3.43 | 9.47 |  | . |  | . |
| IM at Week 5 | Control | 12.41 | 5.75 | Intervention vs Control | 4 | 0.31 (0.16-0.60) | 0.0005 |
|  | Intervention | 3.43 | 9.93 |  | . |  | . |
| IM at Week 5 | Control | 12.41 | 5.75 | Intervention vs Control | 5 | 0.32 (0.17-0.59) | 0.0003 |
|  | Intervention | 3.43 | 10.63 |  | . |  | . |
| IM at Week 5 | Control | 12.41 | 5.75 | Intervention vs Control | 10 | 0.39 (0.20-0.74) | 0.0039 |
|  | Intervention | 3.43 | 15.48 |  | . |  | . |
| IM at Week 5 | Control | 12.41 | 5.75 | Intervention vs Control | 15 | 0.46 (0.25-0.85) | 0.0138 |
|  | Intervention | 3.43 | 20.2 |  | . |  | . |
| IM at Week 5 | Control | 12.41 | 5.75 | Intervention vs Control | 20 | 0.52 (0.28-0.99) | 0.0460 |
|  | Intervention | 3.43 | 24.11 |  | . |  | . |
| IM at Week 5 | Control | 12.41 | 5.75 | Intervention vs Control | 21 | 0.54 (0.29-1.00) | 0.0515 |
|  | Intervention | 3.43 | 24.79 |  | . |  | . |
| IM at Week 5 | Control | 12.41 | 5.75 | Intervention vs Control | 22 | 0.55 (0.30-1.04) | 0.0661 |
|  | Intervention | 3.43 | 25.99 |  | . |  | . |

*Note*. Sensitivity analyses on ITT population. The imputed values for observations in the intervention group are adjusted using the shift parameter. (SHIFT option adds a constant to the imputed value). The baseline number of intrusive memories (at day-7 to -1) is included as a covariate ^1^ Estimated parameters presented using Quasi-Poisson regression model with overdispersion parameter.

Table S14. Sensitivity analysis primary outcome without outliers.

| Model | Outcome | Population | n | Observed Median (IQR) group Control | Observed Median (IQR) group Intervention | Observed Mean (SD) group Control | Observed Mean (SD) group Intervention | Reference group | Estimated Incidence Rate Ratio (95% CI) | p-value |
| --- | --- | --- | --- | --- | --- | --- | --- | --- | --- | --- |
| Quasi-Poisson regression | Number of intrusive memories of traumatic event(s) at week 5 | ITT | 142 | 4 (1-14) | 1 (0-4) | 9.9 (13.2) | 3.8 (7.3) | Control | 0.28 (0.17-0.45) | <0.0001 |
|  | Number of intrusive memories of traumatic event(s) at week 5 | PP | 125 | 5 (1-15) | 1 (0-3) | 10.6 (13.6) | 2.7 (6.3) | Control | 0.26 (0.16-0.42) | <0.0001 |
| Negative Binomial regression | Number of intrusive memories of traumatic event(s) at week 5 | ITT | 142 | 4 (1-14) | 1 (0-4) | 9.9 (13.2) | 3.8 (7.3) | Control | 0.36 (0.22-0.58) | <0.0001 |
|  | Number of intrusive memories of traumatic event(s) at week 5 | PP | 125 | 5 (1-15) | 1 (0-3) | 10.6 (13.6) | 2.7 (6.3) | Control | 0.26 (0.16-0.43) | <0.0001 |

*Note*: Based on imputed data. The baseline number of intrusive memories (at day-7 to -1) is included as a covariate.

Table S15. Sensitivity analysis primary outcome excluding diary missing data (complete diary data only).

| Dataset | Model | Outcome | n | Observed Median (IQR) group Control | Observed Median (IQR) group Intervention | Observed Mean (SD) group Control | Observed Mean (SD) group Intervention | Reference group | Estimated Incidence Rate Ratio (95% CI) | p-value |
| --- | --- | --- | --- | --- | --- | --- | --- | --- | --- | --- |
| Complete (all days reported) | Quasi-Poisson regression | Number of intrusive memories of traumatic event(s) at week 5 | 111 | 6 (1-19) | 1 (0-3) | 13.1 (18.0) | 3.6 (8.8) | Control | 0.27  (0.14-0.52) | 0.0001 |
| Complete (all days reported)±Total number of IM given |  | Number of intrusive memories of traumatic event(s) at week 5 | 118 | 5 (1-17) | 1 (0-3) | 12.4 (17.5) | 3.4 (8.6) | Control | 0.28  (0.15-0.53) | 0.0001 |
| Complete (all days reported) | Negative Binomial regression | Number of intrusive memories of traumatic event(s) at week 5 | 111 | 6 (1-19) | 1 (0-3) | 13.1 (18.0) | 3.6 (8.8) | Control | 0.23  (0.13-0.39) | <0.0001 |
| Complete (all days reported)±Total number of IM given |  | Number of intrusive memories of traumatic event(s) at week 5 | 118 | 5 (1-17) | 1 (0-3) | 12.4 (17.5) | 3.4 (8.6) | Control | 0.23  (0.14-0.40) | <0.0001 |

*Note*: The baseline number of intrusive memories (at day-7 to -1) is included as a covariate. Based on complete data, all days reported and total number of IM given n=118: Control arm n=58, Intervention arm n=60; all days reported n=111: Control arm n=54, Intervention arm n=57.

Table S16. Primary endpoint by gender.

|  | | **MEN** | | **WOMEN** | |
| --- | --- | --- | --- | --- | --- |
|  |  | **Control  n=9** | **Intervention  n=13** | **Control  n=48** | **Intervention  n=47** |
| Number of intrusive memories of traumatic event(s) at week 5 | n |  |  |  |  |
|  | Mean (s.d.) | 8.22 (9.01) | 4.77 (12.94) | 13.23 (18.84) | 3.06 (7.11) |
|  | Median (Q1-Q3) | 4 (3.5-10.5) | 0 (0-2.25) | 5 (1-19.75) | 1 (0-3) |
|  | Min-Max | 2-30 | 0-47 | 0-90 | 0-41 |

*Note.* n=1 in condition Control had gender category ‘other’

Table S17. Primary endpoint by age.

|  | | **Group 1**  **(age= 23-31)** | | **Group 2**  **(age= 32-41)** | | **Group 3**  **(age= 42-50)** | | **Group 4**  **(age= 51-65)** | |
| --- | --- | --- | --- | --- | --- | --- | --- | --- | --- |
|  |  | **Control n=11** | **Intervention  n=19** | **Control  n=17** | **Intervention  n=13** | **CONTROL  n=15** | **Intervention  n=14** | **CONTROL  n=15** | **Intervention  n=14** |
| Number of intrusive memories of traumatic event(s) at week 5 | n |  |  |  |  |  |  |  |  |
|  | Mean (s.d.) | 9.91 (15.44) | 2.53 (5.67) | 13.47 (22.06) | 3.92 (12.96) | 12.67 (15.99) | 1.36 (1.87) | 12.80 (16.21) | 6.29 (10.85) |
|  | Median (Q1-Q3) | 5 (1-11) | 1 (0-2) | 4 (1-20.5) | 0 (0-1) | 8 (2-15) | 0 (0-2.25) | 5 (0-26) | 3.50 (0-6) |
|  | Min-Max | 0-52 | 0-24 | 0-90 | 0-47 | 0-60 | 0-6 | 0-56 | 0-41 |

Table S18. Acceptance and feasibility measure (1 month): feedback about study participation and subsequent use of task by participant on their own.

|  | **Statistics** | **Control n=71** | **Intervention n=73** | **Total n=144** | **Mean Ratio** | **Treatment effect estimate (95% CI)** | **p-value** |
| --- | --- | --- | --- | --- | --- | --- | --- |
| Feedback questionnaire about participation | | | | | | | |
| How easy did you find it to do the task? (0-10) | n | 54 | 47 | 101 |  |  |  |
|  | Mean (s.d.) | 6.61 (2.51) | 7.70 (2.53) | 7.12 (2.56) | 1.17 | 2.37 (1.18-4.85)^a^ | 0.0163 |
|  | Median (Q1-Q3) | 7.0 (5.0-9.0) | 8.0 (6.0-10.0) | 8.0 (5.0-10.0) |  |  |  |
|  | Min-Max | 1.0-10.0 | 0.0-10.0 | 0.0-10.0 |  |  |  |
| How upsetting did you find it to do the task? (0-10) | n | 54 | 47 | 101 |  |  |  |
|  | Mean (s.d.) | 3.11 (3.18) | 1.55 (2.18) | 2.39 (2.85) | 0.50 | 0.37 (0.18-0.75)^a^ | 0.0067 |
|  | Median (Q1-Q3) | 2.0 (0.0-5.0) | 1.0 (0.0-2.0) | 1.0 (0.0-3.0) |  |  |  |
|  | Min-Max | 0.0-10.0 | 0.0-9.0 | 0.0-10.0 |  |  |  |
| How acceptable do you think the task was? (0-10) | n | 54 | 47 | 101 |  |  |  |
|  | Mean (s.d.) | 7.24 (3.06) | 8.70 (1.49) | 7.92 (2.56) | 1.20 | 2.12 (1.05-4.36)^a^ | 0.0382 |
|  | Median (Q1-Q3) | 8.0 (5.0-10.0) | 9.0 (8.0-10.0) | 9.0 (7.0-10.0) |  |  |  |
|  | Min-Max | 0.0-10.0 | 4.0-10.0 | 0.0-10.0 |  |  |  |
| If a friend or colleague had experienced a similar event, how likely is it that you would recommend them to do this task? (0-10) | n | 54 | 47 | 101 |  |  |  |
|  | Mean (s.d.) | 5.09 (3.37) | 8.49 (1.65) | 6.67 (3.19) | 1.67 | 7.85 (3.64-17.63)^a^ | <0.0001 |
|  | Median (Q1-Q3) | 5.5 (2.0-8.0) | 9.0 (7.0-10.0) | 7.0 (5.0-10.0) |  |  |  |
|  | Min-Max | 0.0-10.0 | 4.0-10.0 | 0.0-10.0 |  |  |  |
| Since you did the task for the first time, have you received any other treatment due to traumatic event, e.g. medication or psychological treatment? | Yes (%) | 10 (18.5) | 6 (12.8) | 16 (15.8) |  | 0.64 (0.20-1.89)^b^ | 0.4320 |
|  | No (%) | 44 (81.5) | 41 (87.2) | 85 (84.2) |  |  |  |
| Have you used the task on your own? | Yes (%) | 16 (29.6) | 38 (80.9) | 54 (53.5) |  | 10.03 (4.10-26.74)^b^ | <0.0001 |
|  | No (%) | 38 (70.4) | 9 (19.1) | 47 (46.5) |  |  |  |
| Have you told others about the task (e.g. friend or colleagues)? | Yes (%) | 30 (55.6) | 38 (80.9) | 68 (67.3) |  | 3.38 (1.41-8.69)^b^ | 0.0083 |
|  | No (%) | 24 (44.4) | 9 (19.1) | 33 (32.7) |  |  |  |
| Other comments | Yes (%) | 7 (13.0) | 2 (4.3) | 9 (8.9) |  | 0.30 (0.04-1.31)^b^ | 0.1443 |
|  | No (%) | 47 (87.0) | 45 (95.7) | 92 (91.1) |  |  |  |

*Note.* ^a^ Odds Ratio (OR) – Estimated using proportional odds logistic regression; ^b^ Odds Ratio (OR) – Estimated using binomial logistic regression.

Table S19. Assessments related to procedure: number of hotspots, days/nights worked in diary completion weeks, booster sessions, additional traumatic events during the study.

|  | **Statistics** | **Control n=71** | **Intervention n=73** | **Total**  **n=144** |
| --- | --- | --- | --- | --- |
| Total number of hotspots | n |  | 71 |  |
| Hotspots per participants | Mean (s.d.) | n.a. | 5.06 (3.09) |  |
|  | Median (Q1-Q3) | n.a. | 5 (3-6) |  |
|  | Min-Max | n.a. | 1,16 |  |
| Diary related information | | | | |
| Number of days/nights at work during intrusion diary weeks |  |  |  |  |
| Week 0 (day) | n | 70 | 68 | 138 |
|  | Mean (s.d.) | 3.50 (1.94) | 3.76 (1.94) | 3.63 (1.94) |
|  | Median (Q1-Q3) | 4 (2-5) | 4 (3-5) | 4 (2-5) |
|  | Min-Max | 0, 7 | 0, 7 | 0, 7 |
| Week 0 (night) | n | 70 | 68 | 138 |
|  | Mean (s.d.) | 0.59 (1.30) | 0.68 (1.44) | 0.6 (1.37) |
|  | Median (Q1-Q3) | 0 (0-1) | 0 (-) | 0 (0-0.25) |
|  | Min-Max | 0, 7 | 0, 7 | 0, 7 |
| Week 1 (day) | n | 60 | 57 | 117 |
|  | Mean (s.d.) | 3.38 (1.92) | 3.65 (1.91) | 3.51 (1.92) |
|  | Median (Q1-Q3) | 4 (2-5) | 4 (2-5) | 4 (2-5) |
|  | Min-Max | 0, 7 | 0, 7 | 0, 7 |
| Week 1 (night) | n | 60 | 57 | 117 |
|  | Mean (s.d.) | 0.45 (1.05) | 1.00 (1.54) | 0.72 (1.77) |
|  | Median (Q1-Q3) | 0 (-) | 0 (0-2) | 0 (0-1) |
|  | Min-Max | 0, 5 | 0, 5 | 0, 5 |
| Week 5 (day) | n | 59 | 55 | 114 |
|  | Mean (s.d.) | 3.63 (2.15) | 3.82 (2.00) | 3.72 (2.07) |
|  | Median (Q1-Q3) | 4 (2-5) | 5 (3-5) | 4 (2-5) |
|  | Min-Max | 0, 7 | 0, 7 | 0, 7 |
| Week 5 (night) | n | 59 | 55 | 114 |
|  | Mean (s.d.) | 0.64 (1.40) | 0.71 (1.32) | 0.68 (1.83) |
|  | Median (Q1-Q3) | 0 (0-1) | 0 (0-2) | 0 (0-1) |
|  | Min-Max | 0, 7 | 0, 5 | 0, 7 |
| Number of booster sessions delivered by a researcher | n |  | 90 |  |
|  | Mean (s.d.) |  | 1.23  (1.18) |  |
|  | Median (Q1-Q3) |  | 1 (1-2) |  |
|  | Min-Max |  | 0, 7 |  |
| Type of booster sessions delivered by a researcher |  |  |  |  |
| Full researcher-guided booster | n (%) |  | 8 (8.7%) |  |
| Telephone contact with researcher | n (%) |  | 37 (40.2%) |  |
| Booster by SMS contact with researcher | n (%) |  | 45 (50%) |  |
| Additional work-related trauma since the last contact |  |  |  |  |
| Week 1 | n | 10 | 3 | 13 |
|  | Mean (s.d.) | 2.10 (0.88) | 2.33 (2.31) | 2.15 (1.21) |
|  | Median (Q1-Q3) | 2 (1.75-2.25) | 1 (1 – n.a.) | 2 (1-2.5) |
|  | Min-Max | 1, 4 | 1, 5 | 1, 5 |
| 1 month | n | 6 | 6 | 12 |
|  | Mean (s.d.) | 2.83 (3.60) | 2.33 (1.03) | 2.58 (2.54) |
|  | Median (Q1-Q3) | 1 (1-4.75) | 2 (1.75-3.25) | 2 (1-3) |
|  | Min-Max | 1, 10 | 1, 4 | 1, 10 |
| 3 month | n | 5 | 6 | 11 |
|  | Mean (s.d.) | 3.2 (1.64) | 6.17 (7.08) | 4.82 (5.34) |
|  | Median (Q1-Q3) | 3 (2-4.5) | 3.5(1.75-10.25) | 3 (2-6) |
|  | Min-Max | 2, 6 | 1, 20 | 1, 20 |
| 6 month | n | 6 | 8 | 14 |
|  | Mean (s.d.) | 4.67 (4.18) | 8.13 (10.84) | 6.64 (8.55) |
|  | Median (Q1-Q3) | 2.5 (1.75-10) | 3 (1.25-16.25) | 2.5 (1.75-10) |
|  | Min-Max | 1, 10 | 1, 30 | 1, 30 |
| Additional work-unrelated trauma since the last contact |  |  |  |  |
| Week 1 | n | 10 | 3 | 13 |
|  | Mean (s.d.) | 1.20 (0.42) | 1.33 (0.58) | 1.23 (0.44) |
|  | Median (Q1-Q3) | 1 (1-1.25) | 1 (1- n.a.) | 1 (1-1.5) |
|  | Min-Max | 1, 2 | 1, 2 | 1, 2 |
| 1 month | n | 10 | 7 | 17 |
|  | Mean (s.d.) | 1.80 (1.48) | 1.29 (0.76) | 1.59 (1.23) |
|  | Median (Q1-Q3) | 1 (1-2.5) | 1 (1-1) | 1 (1-1.5) |
|  | Min-Max | 1, 5 | 1, 3 | 1, 5 |
| 3 month | n | 7 | 4 | 11 |
|  | Mean (s.d.) | 1.71 (1.50) | 1.5 (0.58) | 1.64 (1.21) |
|  | Median (Q1-Q3) | 1 (1-2) | 1.5 (1-2) | 1 (1-2) |
|  | Min-Max | 1, 5 | 1, 2 | 1, 5 |
| 6 month | n | 11 | 10 | 21 |
|  | Mean (s.d.) | 5.18 (11.61) | 3.00 (5.98) | 4.14 (9.20) |
|  | Median (Q1-Q3) | 1 (1-2) | 1 (1-1.25) | 1 (1-2) |
|  | Min-Max | 1, 40 | 1, 20 | 1, 40 |

*Note.* Not possible to determine IQR, denoted (-) in the table.

Table S20. Time since traumatic events leading to intrusive memories.

| Time since traumatic event (s) leading to IM  (multiple choice question) | Intrusive memories at week 5 | |
| --- | --- | --- |
|  | Control  Mean / Median /min-max/n | Intervention  Mean / Median /min-max/n |
| Within 24 hours, n=13 | 21.2 / 6.5 / 0-60/ n=6 | 0 /0 /0-0 /n=1 |
| Within a month, n=14 | 11.9 / 6.5 / 1-52 /n=8 | 1.0 / 0.0 / 0-4 /n=4 |
| Between 1-3 months, n=109 | 12.0 / 4.5 / 0-90 /n=44 | 3.8 / 0.0 / 0-47 /n=48 |
| More than 3 months ago, n=63 | 15.2 / 9.5 / 0-60 /n=26 | 5.0 / 1.0 / 0-47 /n=27 |

Procedure-related changes during the study

| **When?** | **What?** |
| --- | --- |
| 12 November 2020 | - Added items assessing examples of negative/positive impact of intrusive memories on functioning to end of week 5 diary (from P002) |
| 21 December 2020 | - Started also including participants working at elderly care and ambulance service - Started taking verbal informed consent in pandemic |
| 13 January 2021 | - Added extra sentence to screening about the traumatic event(s) should have occurred since the start of the COVID-19 pandemic (from P008) |
| 22 March 2021 | - Added WHODAS 2.0 to 6 month follow up (from P005) |
| 25 March 2021 | - Added video to intervention guided session explaining what happens in the brain when playing Tetris summarising intervention rationale and suggestion to repeat the intervention as necessary |
| 22 April 2021 | - Added items assessing how many (additional) work-related and non-work-related traumatic events participants had experienced at baseline (from P029), 1-week (from P023), 1 month (from P018), 3 month (from P011) and 6 month (from P005) follow-ups |
| 07 October 2021 | - Added Concentration and Memory difficulties questionnaire to 6 month follow up (from P021) |
| 09 June 2022 | - Added summary of validated diary data to ‘Kommentarer’ in each diary site (only visible to researchers) for use with trial monitor |
| 16 June 2022 | - Updated SMART-TRIAL settings for items in D7N to non-mandatory after last diary entry by last participant |
